# High Expression of PSRC1 Predicts Poor Prognosis in Lung Adenocarcinoma

**DOI:** 10.7150/jca.88635

**Published:** 2023-10-07

**Authors:** Rui Han, Youhong Guan, Min Tang, Min Li, Binbin Zhang, Guanghe Fei, Sijing Zhou, Ran Wang

**Affiliations:** 1 Department of respiratory and critical care medicine, the first affiliated hospital of Anhui medical university, Hefei 230022, China.; 2 Department of infectious disease, Hefei second people's hospital, Hefei 230001, China.; 3 Department of oncology, the first affiliated hospital of Anhui medical university, Hefei 230022, China.; 4 Department of Occupational Disease, Hefei third clinical college of Anhui Medical University, Hefei 230022, China.

**Keywords:** PSRC1, lung adenocarcinoma, prognosis, immunohistochemistry, bioinformatics

## Abstract

**Background:** The incidence of lung cancer is increasing annually, but the mechanism of its occurrence and development requires further study. This study aimed to investigate the biological function and prognostic value of proline- and serine-rich coiled-coil 1 (PSRC1) in lung cancer.

**Methods:** We used data from The Cancer Genome Atlas (TCGA) to analyze the association between clinical features and PSRC1 expression in non-small cell carcinoma. The relationship between PSRC1 expression and prognosis in lung adenocarcinoma (LUAD) and lung squamous cell carcinoma (LUSC) was analyzed using Kaplan-Meier curves. The function of PSRC1 was identified using enrichment analysis, and the relationship between PSRC1 expression and immune cell infiltration was studied. In addition, the expression of PSRC1 in 150 patients with non-small cell carcinoma was detected using immunohistochemistry, and its clinical significance was analyzed.

**Results:** It was found that the expression level of PSRC1 was higher in LUAD and LUSC tumor tissues than in normal tissues, and the results were confirmed by immunohistochemistry in 150 patients. TCGA data showed that high PSRC1 expression in LUAD was associated with poorer overall survival (*p* = 0.003) and progression-free interval (*p* = 0.012). Multivariable analysis showed that PSRC1 was an independent risk factor for LUAD. Functional enrichment analysis showed that PSRC1 is related to tumor development.

**Conclusion:** High PSRC1 expression is significantly associated with LUAD survival and may be a promising prognostic biomarker.

## Introduction

Lung cancer is the most commonly diagnosed cancer worldwide and the leading cause of cancer-related deaths 1, 2. Lung cancer includes small cell carcinoma and non-small cell carcinoma (NSCLC), which can be further divided include lung adenocarcinoma (LUAD) and lung squamous cell carcinoma (LUSC) 3. Before the 1990s, LUSC accounted for a relatively high proportion of men with NSCLC; however, the incidence of adenocarcinoma gradually surpassed that of squamous cell carcinoma, with LUAD becoming the dominant histological subtype of NSCLC 4, 5. According to statistics, lung cancer accounted for approximately one in nine cancers (11.4%) and one in six deaths (18.0%) in 2020, with an estimated 2.2 million new cancer cases and 1.8 million deaths worldwide^6^. Despite recent advances in the diagnosis and treatment of lung cancer, the prognosis of advanced lung cancer remains poor, with multiple targeted therapies and immune-checkpoint inhibitor-related drugs available 7, 8. Studies have shown that lung cancer is a complex disease involving genetic and epigenetic changes 9, and molecular biological detection plays an important role in improving the early diagnosis rate and prognosis of NSCLC 10-13. Therefore, revealing the intrinsic mechanism of NSCLC, seeking new potential targets, and identifying oncogenes associated with NSCLC prognosis are helpful in the development of new molecular biomarkers necessary for effective diagnostic and therapeutic strategies. With the development of gene chips and high-throughput second-generation sequencing technologies, an increasing amount of genetic data is being stored in public databases for researchers to mine. Therefore, a combination of gene expression data with bioinformatics methods can be used to determine the expression of differentially expressed genes (DEGs) in response to the occurrence and development of NSCLC, and to identify potential targets for treatment 14.

Proline- and serine-rich coiled-coil 1 (PSRC1) is also known as DDA3. PSRC1 is required for normal progression of mitosis, and is involved in a variety of signaling pathways 15, 16. PSRC1 is a microtubule (MT) -associated cancer protein regulated by the downregulation of p53 transcription. Its promoter contains a putative p53-binding motif responsible for p53-mediated gene suppression 17. Further experiments showed that PSRC1 has a unique domain. The C-terminal domain has the ability to bind to the mitotic spindle, while the regulatory N-terminal domain controls the C-terminal domain to bind to microtubules and determines the cellular activity of the PSRC1 protein 16. Studies have shown that PSRC1 is overexpressed in a variety of cancers, including, colorectal cancer 18, hepatocellular carcinoma 19, and oral squamous cell carcinoma 20; and is therefore a potential biomarker and therapeutic target. PSRC1 contains hot spot mutations in colorectal cancer and could potentially be used to develop personalized tumor analysis and therapy 18. PSRC1 hypomethylation may lead to PSRC1 overexpression and tumor progression in hepatocellular carcinoma 19. PSRC1 can be used as a prognostic predictor for oral squamous cell carcinoma without lymph node metastasis 20.

However, there are few studies on PSRC1 in NSCLC. Therefore, this study aimed to investigate the correlation between PSRC1 expression, and clinicopathological features and prognosis of NSCLC; and to clarify the biological role of PSRC1 in NSCLC.

We compared the expression of PSRC1 in LUAD and LUSC in both tumor and normal tissues using the dataset from The Cancer Genome Atlas (TCGA) database, and evaluated the correlation between PSRC1 expression levels and clinicopathological features. Immunohistochemical methods were used to detect differences in the expression of PSRC1 between tumor and normal tissues to verify the prognostic value of PSRC1 in NSCLC. Gene set enrichment analysis (GSEA) was used to identify the biological pathways associated with PSRC1. GSEA and immune-associated infiltration analysis revealed the biological influence of PSRC1 in LUAD and its potential mechanisms of action. Our results strongly support PSRC1 as a biomarker for predicting the prognosis and treatment outcomes of LUAD, but not LUSC. In summary, PSRC1 is a promising prognostic biomarker for LUAD.

## Material and Methods

### Bioinformatics Analysis Based on TCGA Database

We obtained gene expression data, corresponding clinical information, and survival data for TCGA-LUAD (594 samples, workflow type: HTSeq-TPM) and TCGA-LUSC (551 samples, workflow type: HTSeq-TPM) from TCGA. The LUAD dataset included 535 cases of tumors and 59 cases of normal tissue, whereas the LUSC dataset included 502 cases of tumors and 49 cases of normal tissue (https://cancergenome.nih.gov; Table S1). A total of 517 LUAD patients and 496 LUSC patients had complete survival data. We considered the median expression level as the critical value and divided the cases into two groups: the low and the high expression group for PSRC1. Gene expression data (HTSeq-Counts and HTSeq-FPKM), phenotypic data and detailed clinicopathological information of TCGA-LUAD were obtained by browser. Sequence data were retrieved using the Illumina HiSeq_RNA_Seq platform. HTSeq-FPKM gene expression data were converted to TPM (transcripts per million readings) for subsequent analysis. Since TPM produces results that are more similar to those of the microarray method, it is beneficial for comparison between samples 21.

### Sample Collection

We collected 150 samples from patients who had been initially diagnosed with NSCLC. Two pathologists diagnosed lung cancer based on pathological findings, and determined the histological type and staging based on the eighth version of the tumor, node, and metastasis (TNM) staging system. There were 90 and 60 patients with LUAD and LUSC, respectively. Samples were collected from October 2019 to October 2021, and follow-up data were available until December 30, 2022, or the date of death. Tumor tissue samples and paired adjacent normal tissues (normal lung tissue > 5 cm from the tumor margin) were collected from 90 patients diagnosed with LUAD and 60 patients diagnosed with LUSC. Samples were formalin-fixed and paraffin-embedded for immunohistochemical analysis. None of the enrolled patients had a history of other malignant tumors, immune dysfunction, preoperative radiotherapy, or chemotherapy. The following clinical and pathological information was obtained from the enrolled patients: age, sex, surgical modalities, smoking history, tumor size, pathological type, pathological stage, T, N, and M stage; tumor differentiation, postoperative treatment, follow-up, tumor recurrence and progression, overall survival (OS), and progression-free interval (PFI) (Table S2). Sample exclusion criteria were as follows :(1) combined with other malignant tumors; (2) complicated with cardiovascular diseases; (3) the clinical baseline data missing.

### Immunohistochemical Analysis of PSRC1 Expression

The procedure was similar to what was previously described 22. Paraffin-embedded sections were dewaxed in xylene and ethanol, and antigen repair was performed in a citrate buffer solution (pH 6.0). Sections were incubated overnight with anti-PSRC1 antibody (GTX128047:GeneTex; 1:500 dilution) at 4°C and washed with phosphate-buffered saline (PBS). Then, the sections were incubated with HRP-conjugated goat anti-rabbit IgG (ab205718; Abcam) at 37°C for 2 h. Finally, 3, 3-diaminobenzidine tetra hydrochloride (DAB) staining, hematoxylin reverse staining, dehydration, neutral resin sealing, examination, and evaluation were performed. PSRC1 expression was analyzed by immunohistochemistry in tumor and normal tissue samples from 90 patients with LUAD and 60 patients with LUSC. Cytoplasmic staining of the tumor was positive for PSRC1 expression. Two independent pathologists evaluated PSRC1 expression under 400x optical microscopy using a semi-quantitative scoring system, while examining positive and negative controls in parallel 23. The following categories were defined for the evaluation: (i) staining intensity: 0 (negative staining), 1 (mild staining), 2 (moderate staining), 3 (strong staining); (ii) percentage of immunopositive cells (5 fields were randomly selected at 400x magnification per slide): 0 (0%), 1 (1-25%), 2 (26-50%), 3 (51-75%), 4 (76-100%). The percentage of stained cells was multiplied by the fraction of stained cells to obtain the final score (score range: 0-12 points). We considered scores within the 0-2 range as indicating negative expression, within the 3-6 range as indicating low PSRC1 expression, and within the 7-12 range as indicating high PSRC1 expression.

### Enrichment Analyses

We used a median Z-score to divide patients with LUAD into a low- and a high-expression group. DEGs were analyzed using R software with a log fold change (log FC) >1 and an adjusted *p* value <0.05. Volcano and heat maps were created to visualize the analysis results. The core modules and hub genes associated with PSRC1 were identified by weighted gene co-expression network analysis (WGCNA)^24^. We explored the biological function of PSRC1 in LUAD using the R clusterProfiler package for Gene Ontology (GO) terms and Kyoto Encyclopedia of Genes and Genomes (KEGG) analysis 25. GSEA has the advantage of grouping gene sets according to common biological functions, chromosomal regulatory mechanisms, or locations. GSEA was performed using the R package "clusterProfiler" with the following parameters: nPerm = 1000, minGSSize = 10, maxGSSize = 1000, *p* value cutoff = 0.05 26. The enrichment results were considered statistically significant when the false discovery rate (FDR) was <0.25 and the adjusted *p* value was <0.05.

### Immune Infiltrate Analysis

Immunoinfiltration analysis of LUAD was performed using single-sample gene set enrichment analysis (ssGSEA, GSV A package). The ssGSEA algorithm is based on the expression information of specific marker genes in immune cells, which was derived from studies by Bindea et al. 27. We also evaluated the relationship between PSRC1 expression and the abundance of 24 immune cell types. Spearman and Wilcoxon rank-sum tests were used, and *p* < 0.05 was considered statistically significant.

### Statistical Analysis

Kaplan-Meier survival curves were used to estimate survival between patients with high and low gene expression levels and the log-rank test was used to compare differences in survival 28. Univariable and multivariable cox regression analyses were used to determine prognostic factors for LUAD. Factors with statistical significance (p < 0.05) in the univariable analysis were included in the multivariable analysis to determine the independent risk factors. All statistical analyses and plots were performed using the R software (version 4.1.2). Combined with the expression value of PSRC1 and clinical variables, a nomogram was constructed to predict the 1-, 3-, and 5-year OS.

Based on the optimal multivariate cox regression analysis to construct the nomogram, 1 year, 3 years and 5 years survival probability. R package rms (https://cran.r-project.org/web/packages/rms/index.html) is used to generate the column chart. Concordance index (C-index) and calibration plots are often used to evaluate the quality of nomogram models. Using Hmisc R package (https://cran.r-project.org/web/packages/Hmisc/index.html) evaluation C-index and calibration diagram. In this study, the C-index was used to determine the discriminative power of 1000 bootstrap replicates. With the increase of C-index, prediction accuracy increases. The calibration curve was evaluated visually by mapping the nomogram prediction to the observed probability, with the 45° line indicating the best predicted value.

## Results

### High PSRC1 Expression in LUAD and LUSC

**Figure 1:**
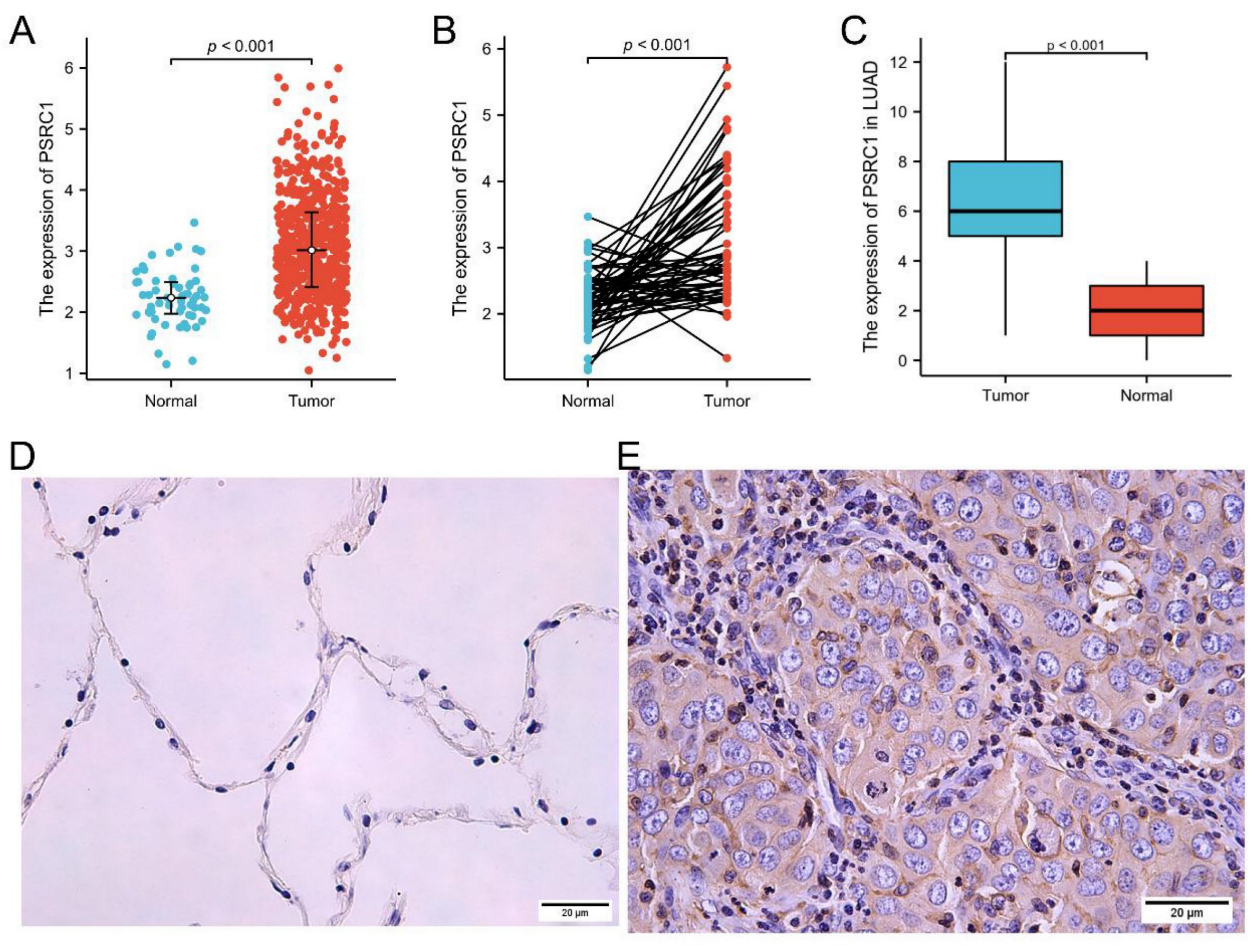
PSRC1 expression in LUAD. (A)PSRC1 expression levels in LUAD and normal tissues in TCGA. (B)PSRC1 expression levels in LUAD and matched normal tissues in TCGA. (C)PSRC1 expression of IHC on 90 patients with LUAD tissues and 90 adjacent normal lung tissues. Representative IHC images of PSRC1 expression in normal tissues (D) and LUAD tissues (E). Abbreviations: PSRC1, Proline and serine rich coiled-coil 1; LUAD, lung adenocarcinoma; TCGA, The Cancer Genome Atlas; IHC, immunohistochemistry.

From the TCGA dataset, 535 patients with LUAD and 502 patients with LUSC met the required clinical characteristics. To detect PSRC1 expression in LUAD and LUSC patients, we compared its expression level in tumor tissues with that in normal lung tissues. The results showed that the expression level of PSRC1 in LUAD and LUSC tissues was significantly higher than that in normal tissues (*p* < 0.001; Figure 1A and S1A), and that in the tumor tissue was higher than that in the paired normal tissue (*p* < 0.001; Figure 1B and S1B). To verify the differences in PSRC1 expression, immunohistochemical analyses were performed on normal and tumor tissues from 90 patients with LUAD and 60 patients with LUSC. The results were validated in LUAD, LUSC, and paired normal lung tissues (*p* < 0.001; Figure 1C and S1C). Representative immunohistochemical images are shown in Figure 1D, 1E, S1D and S1E.

### Expression of PSRC1 in relation to clinicopathological features of LUAD and LUSC from the TCGA dataset

**Figure 2:**
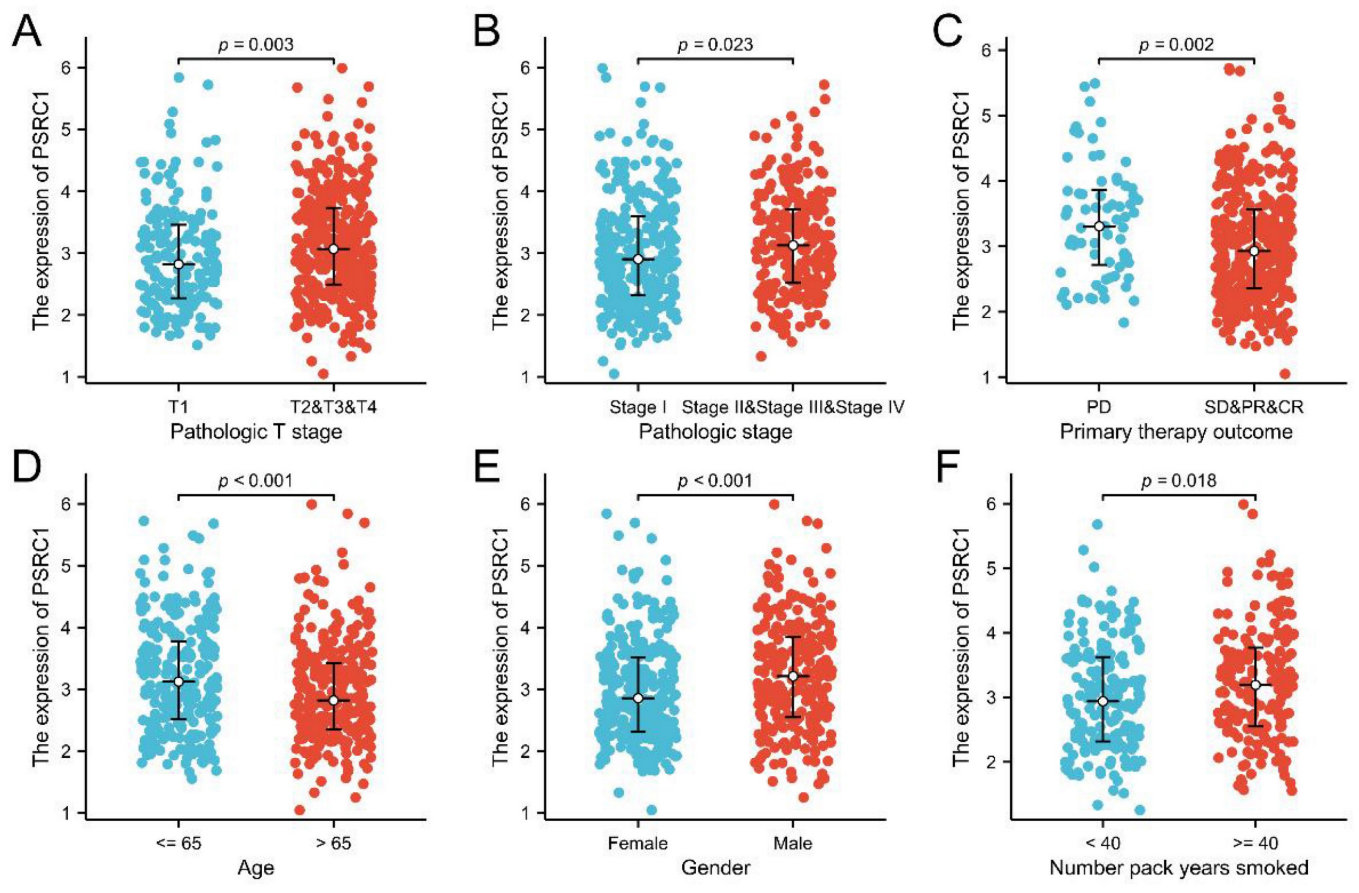
Correlation between PSRC1 expression and clinicopathologic features in patients with LUAD in TCGA database (*p* < 0.05). The correlation between PSRC1 expression and pathologic T stage (A), pathologic stage (B), primary therapy outcome (C), age (D), gender (E) and number pack year smoked (F) of patients with LUAD. Abbreviations: PSRC1, Proline and serine rich coiled-coil 1; LUAD, lung adenocarcinoma; TCGA, The Cancer Genome Atlas.

The expression of PSRC1 was different according to age, sex, smoking history, T stage, and pathological stage group in LUAD patients (Figure 2). The analysis showed that the expression of PSRC1 in LUAD patients at T2/T3/T4 was higher than that in patients at T1 (*p* = 0.003; Figure 2A), the expression of PSRC1 in LUAD patients with a high pathological stage was higher than in those with a low pathological stage (*p* = 0.023; Figure 2B), and the expression of PSRC1 in LUAD patients with improved or stable primary therapy outcome was lower than that in those with tumor progression (*p* = 0.002; Figure 2C). Older LUAD patients exhibited lower expression compared to patients under 65 years (*p* < 0.001; Figure 2D), and male LUAD patients showed higher expression than female LUAD patients (*p* < 0.001; Figure 2E). PSRC1 expression in LUAD patients with long smoking duration was higher than that in LUAD patients with short smoking duration (*p* = 0.018; Figure 2F).

In LUSC, high PSRC1 expression was not correlated with clinical features, as shown in Figure S2A-H.

### High expression of PSRC1 was an independent risk factor for poor prognosis in LUAD

**Figure 3:**
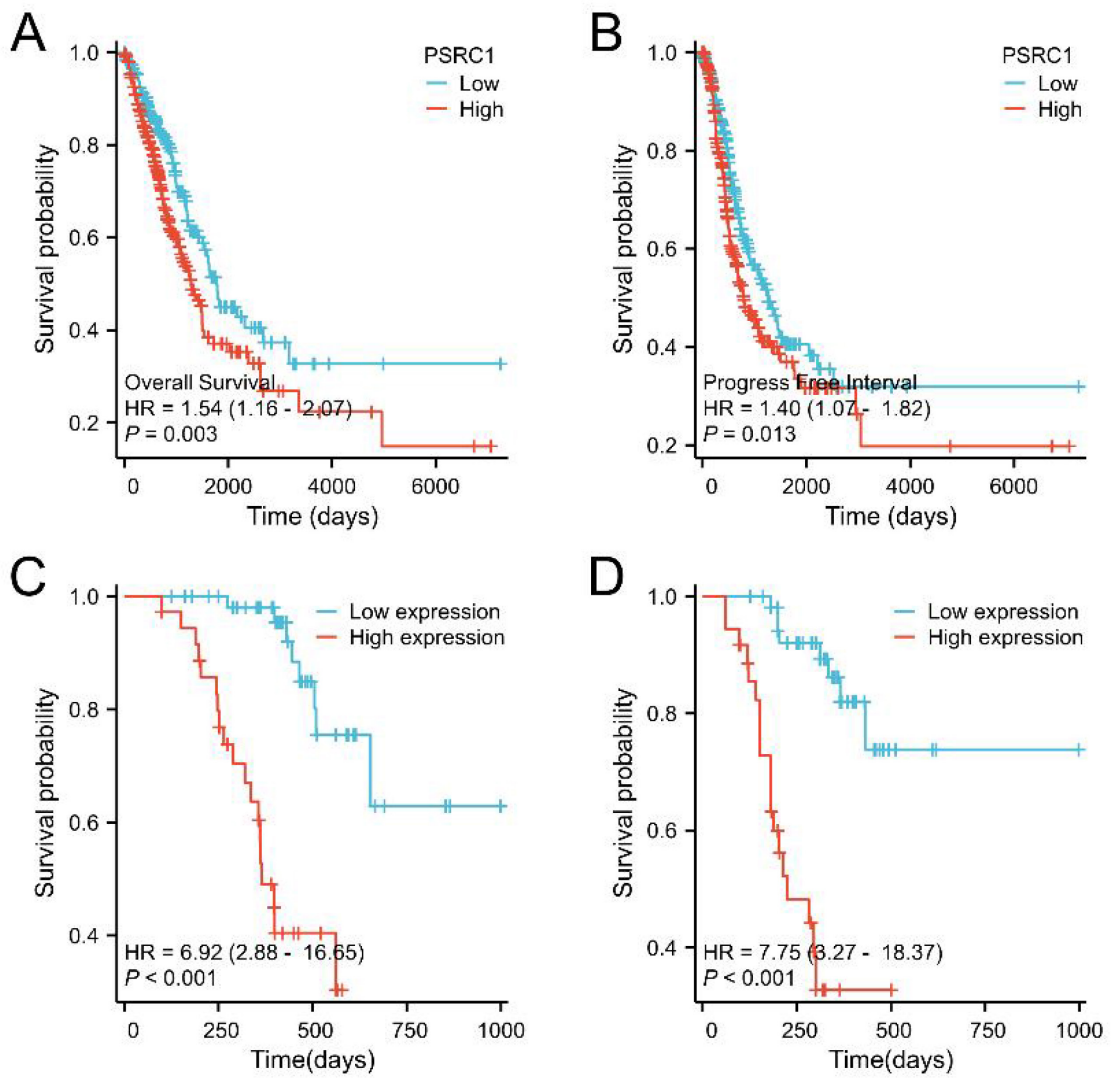
Kaplan-Meier curve for OS in LUAD. (A) Overall survival and (B) progress free interval. Kaplan-Meier survival curves of 90 LUAD patients with high and low PSRC1-expressing tumors (C) Overall survival and (D) progression free interval.

Kaplan-Meier analysis was used to estimate survival rates in LUAD and LUSC patients. Figure 3A, 3B, S3A and S3B included patient death and disease recurrence as the end point events, respectively, and the two survival curves were compared using the log-rank test. The cox proportional hazards regression model was used to analyze the influencing factors of OS and PFI in figure 3C, 3D, S3C and S3D. The results showed that the survival time of LUAD tumor patients with high PSRC1 expression was shorter than that of patients with low PSRC1 expression (OS, *p* = 0.003, Figure 3A; and PFI, *p* = 0.013, Figure 3B). This conclusion is consistent with the results obtained in 90 patients with LUAD (OS, Figure 3C; PFI, Figure 3D; *p* < 0.001). However, no significant correlation was observed in the TCGA LUSC dataset (Figure S3A and B; *p* > 0.05) or in the 60 LUSC patients (Figure S3C and D; *p* > 0.05).

We performed a further subgroup analysis and found that high expression of PSRC1 significantly decreased OS at the LUAD T1/T2 stage (*p* = 0.013), N0 stage (*p* = 0.03), M0 stage (*p* = 0.012), and pathological stages I/II/III (*p* = 0.005); in patients aged > 65 years (*p* = 0.003), and in female patients (*p* = 0.006; Figure S4A-F). These results confirmed that high PSRC1 expression is associated with poor prognosis in patients with LUAD.

Since no correlation between PSRC1 expression and LUSC prognosis was observed in the TCGA dataset and the clinical data of 60 LUSC cases, univariable and multivariable cox regression and enrichment analyses were conducted only for LUAD cases. In TCGA, univariable cox regression analysis showed that the expression of PSRC1, TNM stage, pathological stage, and primary therapy outcome were correlated with OS, while PSRC1 expression, T stage, N stage, and primary therapy outcome were correlated with PFI. Multivariable analysis showed that PSRC1 expression, T stage, N stage, and primary therapy outcome were independent risk factors for OS in LUAD patients, while PSRC1 expression, T stage, and primary therapy outcome were independent risk factors for PFI (Table 1). Univariable and multivariable cox analyses were performed on a cohort of 90 patients with LUAD. Univariable analysis revealed that N stage, differentiation, and PSRC1 expression were prognostic factors for OS. The univariable analysis results for PFI were similar to those for OS (Table 2). Multivariable analysis was conducted using variables with *p* values <0.05 in univariable analysis, and the results showed that high expression of PSRC1 was an independent risk factor for OS and PFI in patients with LUAD. The results are shown in Table 2.

### Construction of a Nomogram

**Figure 4:**
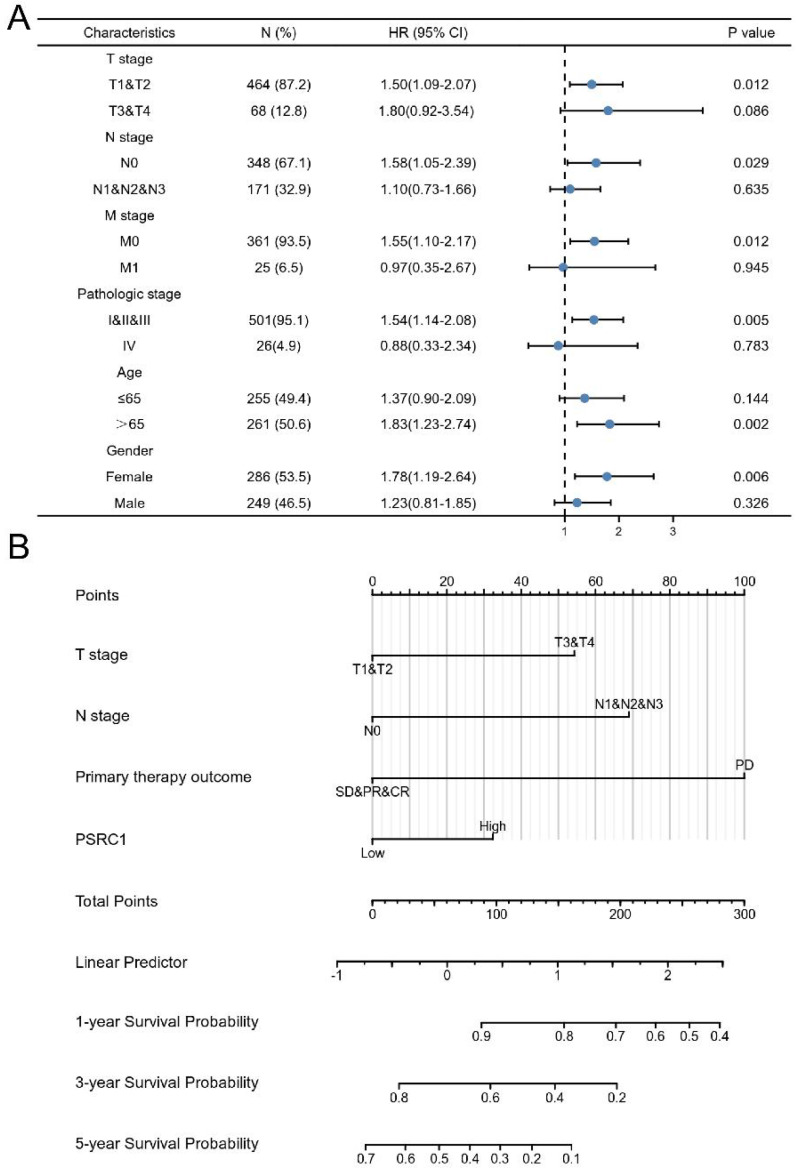
Relationship between PSRC1 expression and different clinicopathological factors and overall survival. (A) Analysis of PSRC1 expression on survival in different clinical subgroups of LUAD patients. (B) Nomogram for predicting probability of patients with 1-, 3- and 5-year OS.

We constructed a prognostic nomogram by weighing the TNM stage, primary treatment outcome, age, sex, and PSRC1 expression. Figure 4A shows a forest map based on the survival analysis of different clinical subgroups of LUAD patients with PSRC1 expression. The prognosis map includes clinical features independently associated with survival in the multivariable analysis (i.e., high PSRC1 expression, T stage, N stage, and primary treatment outcomes; Figure 4B). These results also confirm that high PSRC1 expression is associated with poor prognosis. Nomogram discrimination was evaluated using c-index. In our prognosis chart, LUAD's c-index of LUAD was 0.815(95%CI:0.771-0.860). As shown in the calibration diagram (Figure S5), the predicted values were in good agreement with the observed values.

### Correlation and Enrichment Analyses

**Figure 5:**
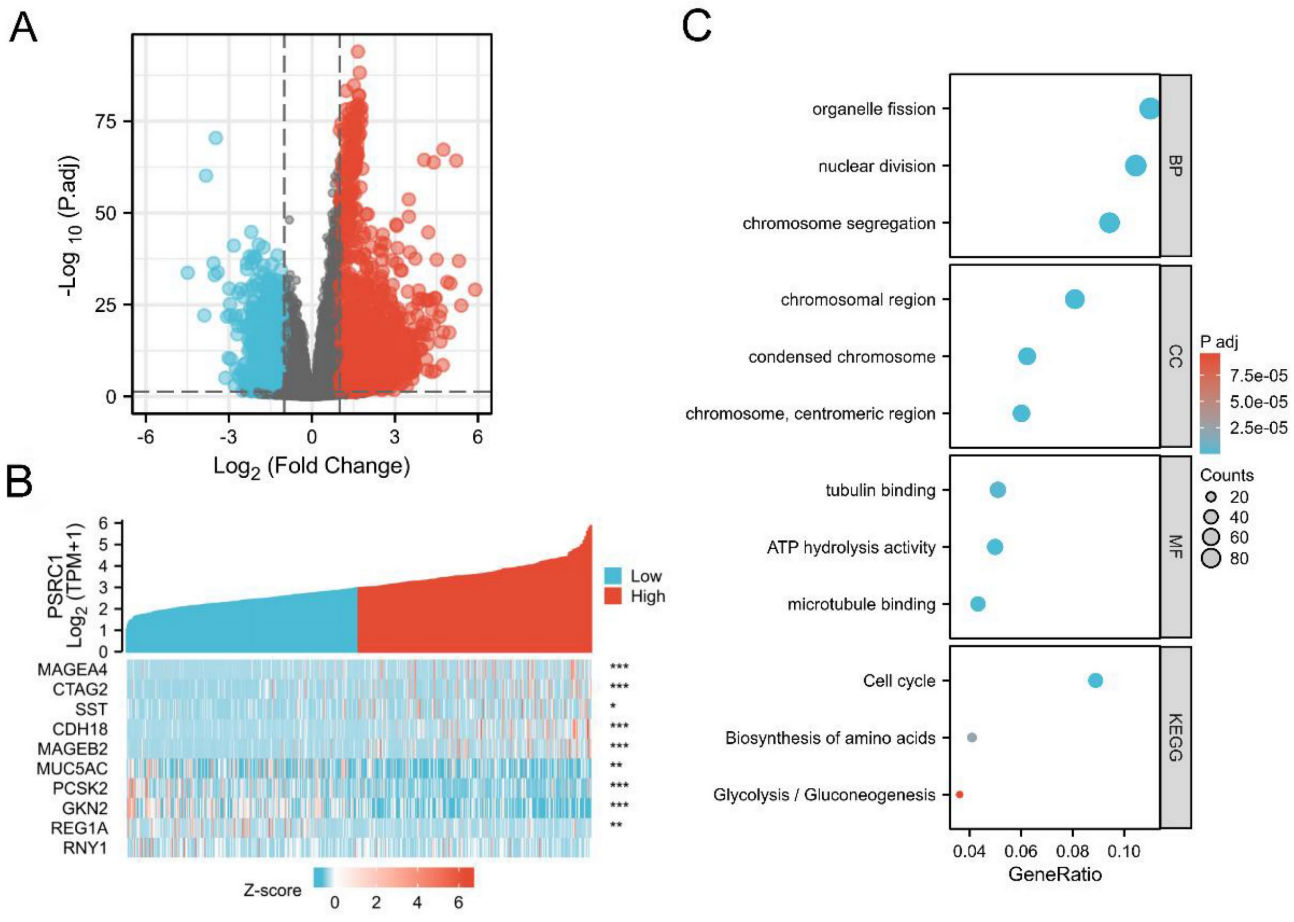
Differentially expressed genes in LUAD patients with high and low PSRC1 expression. (A) Volcano map of differentially expressed genes based on PSRC1 expression. (B) Heat maps of the first five up-regulated and the first five down-regulated genes were selected according to the expression status of PSRC1. (Pearson). (C) GO/KEGG enrichment analysis of PSRC1 expression-related genes. *p < 0.05; ** p < 0.01; *** p < 0.001. Abbreviations: PSRC1, Proline and serine rich coiled-coil 1; LUAD, lung adenocarcinoma; GO, Gene Ontology.

We used TCGA data to analyze the correlation between PSRC1 and other genes in LUAD to predict the function of PSRC1. We used R (| logFC | > 1, adjusted *p* values < 0.05) analysis of the TCGA dataset (Figure 5). PSRC1 between the high- and low-expression groups identified 3703 DEGs (2200 upregulated and 1503 downregulated). Volcano and heat maps were created to visualize the results (Figure 5A and B). The brown core module associated with PSRC1 was identified by WGCNA, so we performed enrichment analysis with the brown module using the clusterProfiler R package (Figure S6). Figure 5C shows the most important GO terms for biological processes, cellular components, and molecular functions. In LUAD, GO analysis revealed 326 GO terms associated with the biological processes, indicating that PSRC1 is involved in many biological processes such as organelle fission, nuclear division, and chromosome segregation. 44 GO terms associated with the cell component category "chromosome". And there are 21 GO terms related to molecular functions, such as tubulin binding, ATP hydrolysis activity and microtubule binding. According to KEGG functional enrichment analysis, related genes were involved in cell cycle, biosynthesis of amino acids and glycolysis/gluconeogenesis.

**Figure 6:**
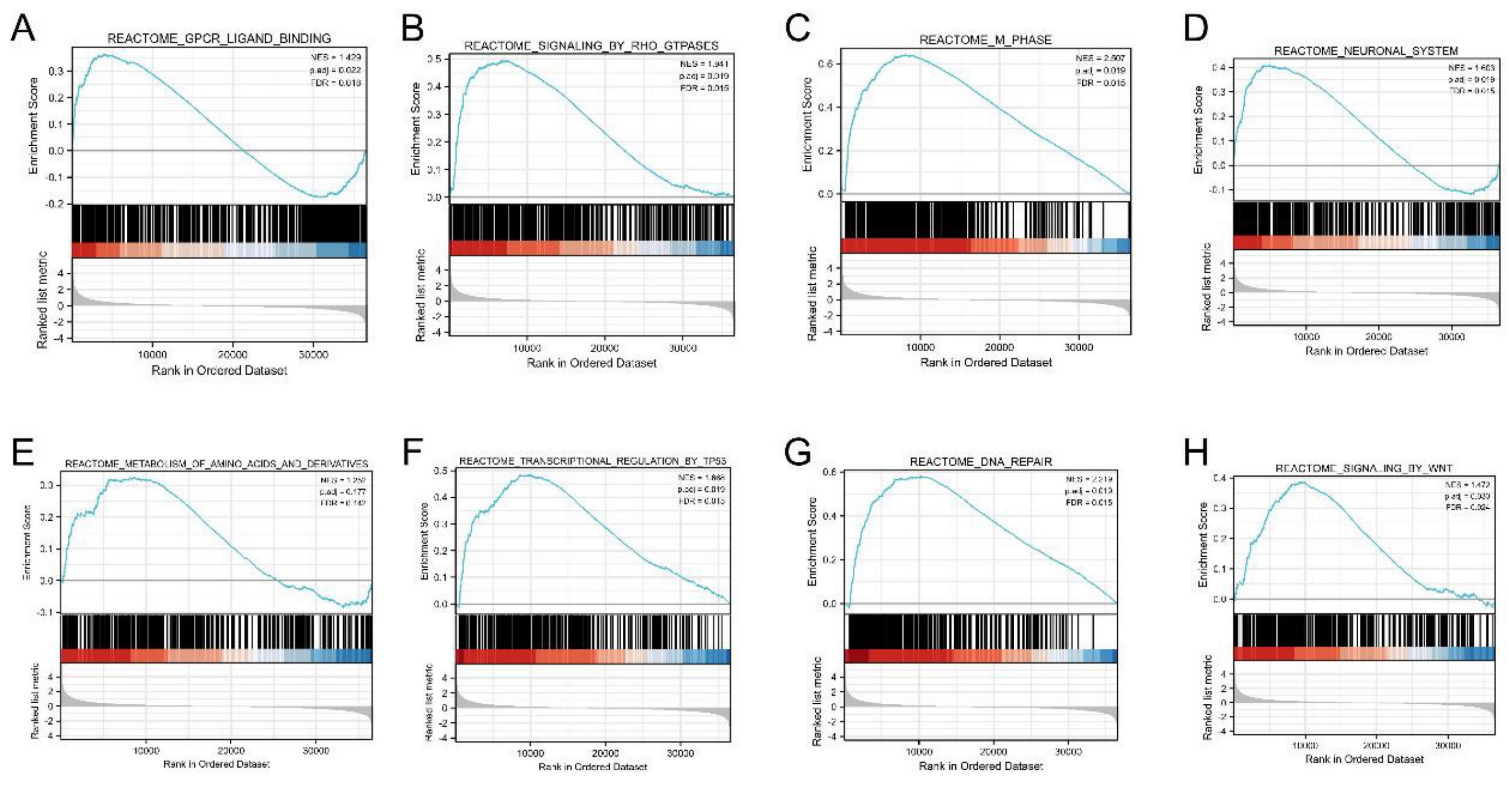
Gene set enrichment analysis (GSEA) enrichment plot. (A) GPCR ligand binding, (B) signaling by RHO GTPases, (C) M phase, (D) neuronal system, (E) transcriptional regulation by Tp53, (F) DNA repair, (G) signaling by WNT, (H) metabolism of amino acids and derivatives.

We performed GSEA to determine the signaling pathway activated in LUAD, and adjusted *p* values < 0.05 and FDR < 0.25 were considered significant. The results showed that the following eight pathways were significantly different between the two groups: G-protein couple receptor (GPCR) ligand binding, signaling by RHO GTPases, M phase, neuronal system, transcriptional regulation by Tp53, DNA repair, signaling by WNT, and metabolism of amino acids and derivatives (Figure 6A-H).

### Correlation between immune cell infiltration and PSRC1 in LUAD

**Figure 7:**
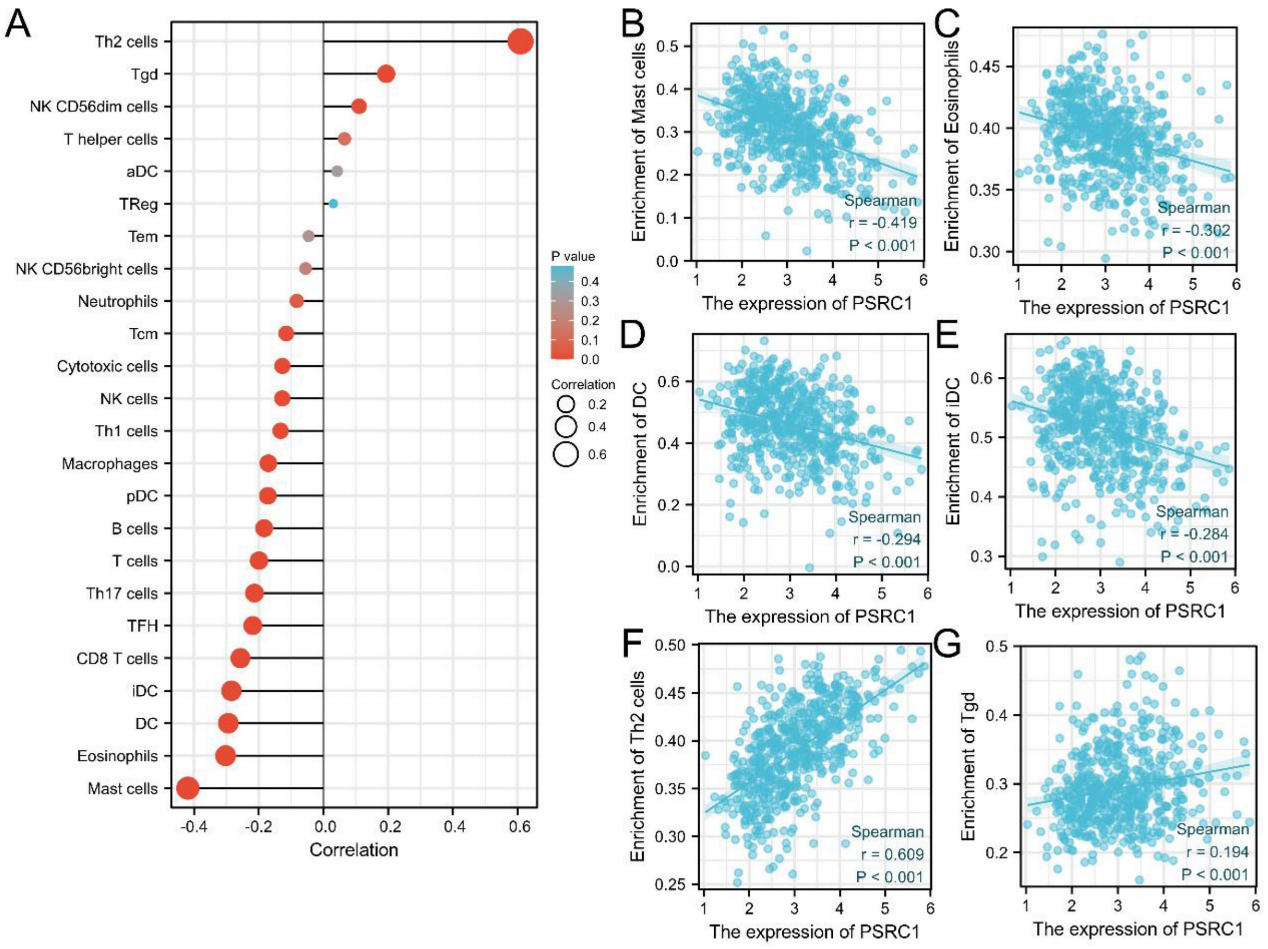
Correlation between immune cell infiltration and PSRC1 in LUAD. (A) Correlation between PSRC1 expression level in LUAD and infiltration of 24 immune cells. Relationship between PSRC1 expression and infiltration of (B) mast cells, (C) eosinophils, (D) dendritic cells (DC), (E) immature dendritic cells (iDC), (F) type 2 T-helper (Th2) cells and (G) γδ T (Tgd) cells.

The survival of cancer patients is associated with immune cell infiltration. Spearman's r was used to analyze the correlation between PSRC1 expression levels in LUAD and infiltration of 24 immune cell types (Figure 7A). The results showed that the expression of PSRC1 was negatively correlated with mast cell, eosinophil, dendritic cell (DC), and immature dendritic cell (iDC) infiltration (*p* < 0.001; Figure 7B-E), and positively correlated with type 2 T-helper (Th2) and γδ T (Tgd) cell infiltration (*p* < 0.001; Figure 7F-G).

## Discussion

Lung cancer, a complex disease involving genetic and epigenetic changes, is the leading cause of cancer-related deaths worldwide. Lung adenocarcinoma is the most important subtype of lung cancer, and its incidence is on the rise 5. Personalized, biomarker-driven therapy and screening in high-risk groups have led to improvements in LUAD's survival rate 28. Lung cancer has a 5-year survival rate of 10-20%, depending on the stage and region 6. Treatment for LUAD usually involves a combination of surgery, chemotherapy, and/or radiation; and in recent years the development of various chemotherapies, advances in targeted therapy, and immunotherapy have provided hope for the treatment of lung cancer 29-31. However, owing to the lack of early diagnosis, most lung cancers are only detected in advanced stages of local tumor invasion or distant metastasis. A comprehensive study by the Tyrol Registry has shown that most patients with NSCLC are already in an advanced stage at the time of initial diagnosis and have multiple comorbidities, which limits treatment options 32. Therefore, it is critical to identify biomarkers for tumorigenesis and poor prognosis in these patients.

PSRC1 is a novel microtubule-associated protein that encodes a serine- and proline-rich protein containing a coiled-coil region and six putative SH3-domain-binding motifs, PXXP 33. PSRC1 interacts with the MT depolymerase Kif2a in a microtubule-dependent manner, recruiting Kif2a and ANKRD53 to the mitotic spindle and spindle pole 34. It was reported that PSRC1, as a p53 target gene, interacts physically and functionally with microtubules and their associated proteins EB3 and APC2, and enhances the expression of β-catenin-dependent genes, which provides the basis for the functional connection between the p53 and the β-catenin pathways, and may be involved in the role of p53 in microtubule-dependent activities 33. PSRC1 may also be involved in regulating neurite formation and elongation 35. Proper regulation of mitotic spindle dynamics is key to successful cell division, and PSRC1 plays a role in this regulation. It was found that the suppressor of Cullin 5-interacting suppressor of the cytokine signaling box protein ASB7 ubiquitinates the regulator PSRC1 for proteasomal degradation. ASB7 may play a critical role in regulating spindle dynamics and genome integrity by controlling PSRC1 expression 36.

Previous studies have demonstrated that PSRC1 is a biomarker of cardiovascular disease 37-39, and there is increasing evidence that PSRC1 is involved in the occurrence and progression of multiple cancers. PSRC1 is regulated by the downregulation of p53 transcription and is responsible for p53-mediated gene inhibition, which plays an important role in human tumors 17. As reported by Wei et al., PSRC1 is involved in the prognosis of patients with hepatocellular carcinoma 40.

Our study suggests that PSRC1 is a potential therapeutic target and prognostic predictor. Bioinformatics analysis was performed using RNA-seq data from TCGA to assess the prognostic value of PSRC1 in LUAD and LUSC. Survival analysis revealed the prognostic value of PSRC1 in patients with LUAD. High PSRC1 expression was associated with poor OS and PFI in patients with LUAD, but this was not the case for LUSC. The results were confirmed by immunohistochemistry of the clinical samples. In addition, we also found significant differences in the expression levels of PSRC1 at different TNM stages (T1/T2, N0, M0), pathological stages (I/II/III), in advanced age (> 65 years), and in female patients, which may influence the prognosis of LUAD patients. These results suggest that PSRC1 acts as an oncogene in LUAD. To date, no studies have reported the role of PSRC1 in the progression of LUAD, and therefore these new results may provide a new avenue for exploring new treatment options for this particular type of cancer.

Since our results showed that PSRC1 expression had no significant effect on the prognosis of LUSC patients, functional enrichment and immunoinfiltration analyses were performed only for LUAD patients. Campbell et al. examined the exome sequences and copy number profiles of 1,144 types of lung cancer genes and found that mutated genes and somatic cell copy number changes were significantly different between the two types of lung cancer 41. This is consistent with gene expression studies showing that oncogenic drivers may differ to a large extent between lung adenocarcinoma and lung squamous cell carcinoma, and somatic alterations may have different oncogenic potential in different cellular environments 42. These differences may explain the different sensitivities of LUAD and LUSC to immunotherapy and targeted therapy 43.

Enrichment analysis indicated that PSRC1 expression is related to various biological processes, such as organelle fission, nuclear division, and chromosome segregation, and plays an important role in the cell cycle. Downregulation of PSRC1 controls mitosis by increasing the frequency of misaligned chromosomes in metaphase cells and the metaphase delay, and decreasing the metaphase intercentromere tension and the anaphase chromosome separation rate 15. Our results suggest that high PSRC1 expression may accelerate mitosis, promote cell migration and proliferation, and lead to tumor development, which is consistent with previous reports in other types of cancer 19, 44. Tumor genomes are usually abnormal due to mutagenesis damage and incomplete DNA repair. DNA repair has recently received considerable attention as a therapeutic target 45. GSEA showed enrichment in the expression of the PSRC1 gene, which also suggests PSRC1 as a new target for anticancer therapy in lung cancer.

It is well known that the immune system represents an important barrier to the formation and development of tumors, and escaping immune destruction is one of the hallmarks of cancer 46. Tumors use tumor-induced damage to antigen presentation, activation of negative co-stimulatory signals, and immunosuppression to thwart immune responses, and cell populations such as regulatory T cells, natural killer T cells, and different subpopulations of immature and mature dendritic cells may contribute to this immunosuppressive network 47. The ability of of NSCLC to escape immune vigilance is determined by the level of immune invasion and the existence of an immune escape mechanism; the invasiveness of tumors is related to the immune regulatory function of the body 48. Immunotherapy aims to promote the activity of cytotoxic T lymphocytes in tumor, T cell immune infiltrating level is related to the curative effect of immunotherapy, so is the current focus in the study of cancer immunotherapy 49. Studies have shown that high T cell expression is associated with better OS in many types of cancer, including breast cancer, lung cancer, and melanoma 50. Dendritic cells are professional antigen-presenting cells that influence anti-tumor immune responses by displaying a wide range of dysfunctional states in the tumor microenvironment. DC-based therapy can restore the function of DC in the tumor microenvironment, thus showing promise in tumor therapy 51. Our results showed that PSRC1 expression level was correlated with immune infiltration, including levels of T cells, mast cells and dendritic cells. These results suggest that the expression level of PSRC1 may indicate the level of immune infiltration and provide a reference for the application of immunotherapy in LUAD.

We further investigated the relationship between PSRC1 expression and immune cell infiltration in LUAD patients. The results showed that PSRC1 expression was negatively correlated with mast cell, eosinophil, DC, and iDC infiltration, and positively correlated with Th2 and Tgd cell infiltration. Studies have shown that mast cells and eosinophils may have therapeutic targets and may also improve cancer immunotherapy 52, 53. In fact, NSCLC tumors are infiltrated by T cells 54, and human primary NSCLC is Th2-skewed and contains many tumor-promoting Treg cells 55. Some symbiotic microbiota can promote lung cancer development through Tgd cells 56. In summary, PSRC1 may alter the prognosis of patients with LUAD by influencing immune cell infiltration.

Our study has some limitations. First, our dataset is derived from a single medical institution, and the number of clinical samples is relatively small. Therefore, studies involving a larger number of samples are still needed to verify the results. Second, our results mainly focused on LUAD. Bioinformatics analysis of the online dataset revealed the relationship between PSRC1 expression and immune cell infiltration, but no significant correlation was found for LUSC. Therefore, more experiments with larger sample sizes are needed to study the specific mechanism of PSRC1 in the occurrence and development of NSCLC.

## Conclusion

Through bioinformatics analysis based on online datasets, our study showed that PSRC1 expression in tumor tissues was higher than that in normal tissues in both LUAD and LUSC. By collecting clinical samples, we focused on immunohistochemical and survival analyses of NSCLC. The results showed that high PSRC1 expression was significantly associated with poor prognosis in LUAD, but no correlation was observed in the case of LUSC. Therefore, PSRC1 may be a promising prognostic biomarker that could serve as a valuable therapeutic target for LUAD patients.

## Supplementary Material

Supplementary figures and tables.

## Figures and Tables

**Table 1:** Correlations Between PSRC1 Expression and Overall Survival in Patients with Lung Adenocarcinoma

Characteristics	Total (N)	OS				PFI			
		Univariable analysis		Multivariable analysis		Univariable analysis		Multivariable analysis	
		Hazard ratio (95% CI)	P value	Hazard ratio (95% CI)	P value	Hazard ratio (95% CI)	P value	Hazard ratio (95% CI)	P value
T stage	527	2.352 (1.614 - 3.426)	< 0.001	1.801 (1.056 - 3.073)	< 0.05	1.840 (1.268 - 2.670)	0.001	1.909 (1.286 - 2.833)	0.001
T1&T2	461								
T3&T4	66								
N stage	514	2.547 (1.904 - 3.407)	< 0.001	1.904 (1.278 - 2.836)	< 0.01	1.484 (1.131 - 1.948)	0.004	1.117 (0.832 - 1.501)	0.461
N0	345								
N1&N2&N3	169								
M stage	381	2.176 (1.272 - 3.722)	< 0.01	1.473 (0.692 - 3.138)	0.315	1.542 (0.872 - 2.728)	0.137		
M0	356								
M1	25								
Pathologic stage	522	2.338 (1.397 - 3.912)	< 0.01						
Stage I & Stage II & Stage III	496								
Stage IV	26								
Primary therapy outcome	442	0.272 (0.191 - 0.388)	< 0.001	0.246 (0.158 - 0.383)	< 0.001	0.165 (0.122 - 0.223)	< 0.001	0.176 (0.129 - 0.241)	< 0.001
PD	71								
SD&PR&CR	371								
Age	520	1.216 (0.910 - 1.625)	0.186			1.019 (0.781 - 1.330)	0.889		
<=65	257								
>65	263								
Gender	530	1.087 (0.816 - 1.448)	0.569			1.189 (0.914 - 1.547)	0.197		
Female	283								
Male	247								
Race	472	1.493 (0.913 - 2.440)	0.110	1.411 (0.670 - 2.970)	0.365	1.188 (0.792 - 1.781)	0.406		
Asian&Black or African American	63								
White	409								
PSRC1	530	1.545 (1.155 - 2.065)	< 0.01	1.770 (1.174 - 2.667)	< 0.01	1.400 (1.075 - 1.823)	0.013	1.334 (1.005 - 1.770)	0.046
Low	266								
High	264								

**Table 2:** Univariable and Multivariable Analyses of OS and PFI in 90 Patients with Lung Adenocarcinoma

Characteristics	Total(N)	OS				PFI			
		Univariable analysis		Multivariable analysis		Univariable analysis		Multivariable analysis	
		Hazard ratio (95% CI)	P value	Hazard ratio (95% CI)	P value	Hazard ratio (95% CI)	P value	Hazard ratio (95% CI)	P value
Age	90	0.795 (0.373-1.695)	0.553			0.832 (0.390-1.773)	0.634		
<=65	49								
>65	41								
Gender	90	1.254 (0.588-2.672)	0.558			1.366 (0.641-2.912)	0.419		
Male	50								
Female	40								
Smoker	90	1.113 (0.520-2.384)	0.783			1.089 (0.509-2.332)	0.826		
Yes	50								
No	40								
Pathologic stage	90	0.000 (0.000-Inf)	0.997			0.000 (0.000-Inf)	0.997		
I & II & III	87								
IV	3								
T stage	90	1.049 (0.361-3.053)	0.930			0.935 (0.320-2.730)	0.903		
T1&T2	78								
T3&T4	12								
N stage	90	0.209 (0.091-0.480)	<0.001	0.065 (0.008-0.537)	0.011	0.203 (0.088-0.466)	<0.001	0.195 (0.082-0.469)	<0.001
N0	54								
N1&N2	36								
M stage	90	0.000 (0.000-Inf)	0.997			0.000 (0.000-Inf)	0.997		
M0	87								
M1	3								
Differentiation	90	5.383 (2.123-13.647)	<0.001	3.262 (1.083-9.831)	0.036	4.927 (1.952-12.437)	<0.001	2.182 (0.745-6.396)	0.155
Well	41								
Moderate& Poor	49								
PSRC1 Expression	90	6.920 (2.877-16.645)	<0.001	4.264 (1.626-11.181)	0.003	7.752 (3.271-18.372)	<0.001	6.292 (2.279-17.370)	<0.001
Low expression	54								
High expression	36								

## Abbreviations

DEGs: differentially expressed genes
DC: dendritic cell
GSEA: Gene set enrichment analysis
GPCR: G-protein couple receptor
iDC: immature dendritic cell
KEGG: Kyoto Encyclopedia of Genes and Genomes
NSCLC: non-small cell carcinoma
LUAD: lung adenocarcinoma
LUSC: lung squamous cell carcinoma
MT: microtubule
PBS: phosphate-buffered saline
PFI: progression-free interval
PSRC1: Proline- and serine-rich coiled-coil 1
OS: overall survival
ssGSEA: single-sample gene set enrichment analysis
TCGA: The Cancer Genome Atlas
Th2: type 2 T-helper
TNM: tumor, node, and metastasis
