# Supplementary material for: High Expression of PSRC1 Predicts Poor Prognosis in Lung Adenocarcinoma
Source: J Cancer. 2023 Oct 7;14(17):3321–34. doi: 10.7150/jca.88635 (PMC10622992; doi:10.7150/jca.88635)
Supplement: Supplementary file 1 — Supplementary figures and tables. [file jcav14p3321s1.pdf]

Supplements

Figure S1. PSRC1 expression in LUSC. (A)PSRC1 expression levels in LUSC and normal tissues in TCGA. (B)PSRC1 expression levels in LUSC and matched normal tissues in TCGA. (C)PSRC1 expression of IHC on 60 patients with LUSC tissues and 60 adjacent normal lung tissues. (D) Representative IHC images of PSRC1 expression in normal tissues and (E) LUSC tissues.

PSRC1, Proline and serine rich coiled-coil 1; LUSC, lung squamous cell carcinoma; TCGA, The Cancer Genome Atlas; IHC, immunohistochemistry.

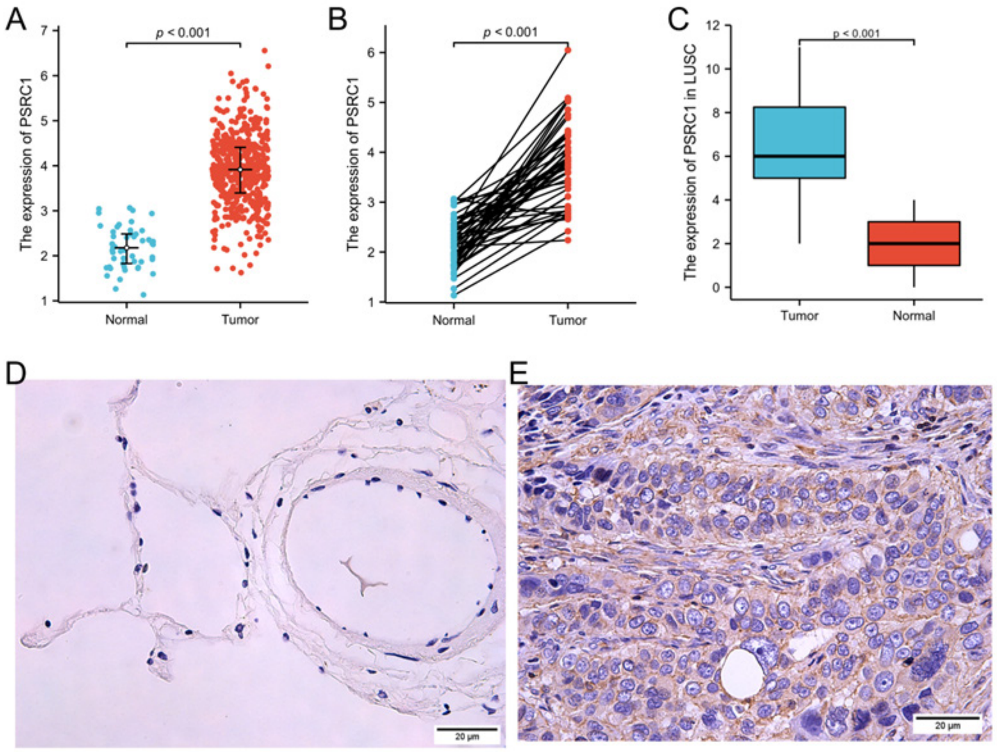

A
p < 0.001
The expression of PSRC1
Normal
Tumor
B
p < 0.001
The expression of PSRC1
Normal
Tumor
C
p < 0.001
The expression of PSRC1 in LUSC
Tumor
Normal
D
20 μm
E
20 μm

Figure S2. Association of PSRC1 expression with clinicopathologic characteristics in LUSC. (A) T stage, (B) N stage, (C) M stage, (D) pathologic stage, (E) gender, (F) race, (G) age and (H) smoker.

PSRC1, Proline and serine rich coiled-coil 1; LUSC, lung squamous cell carcinoma.

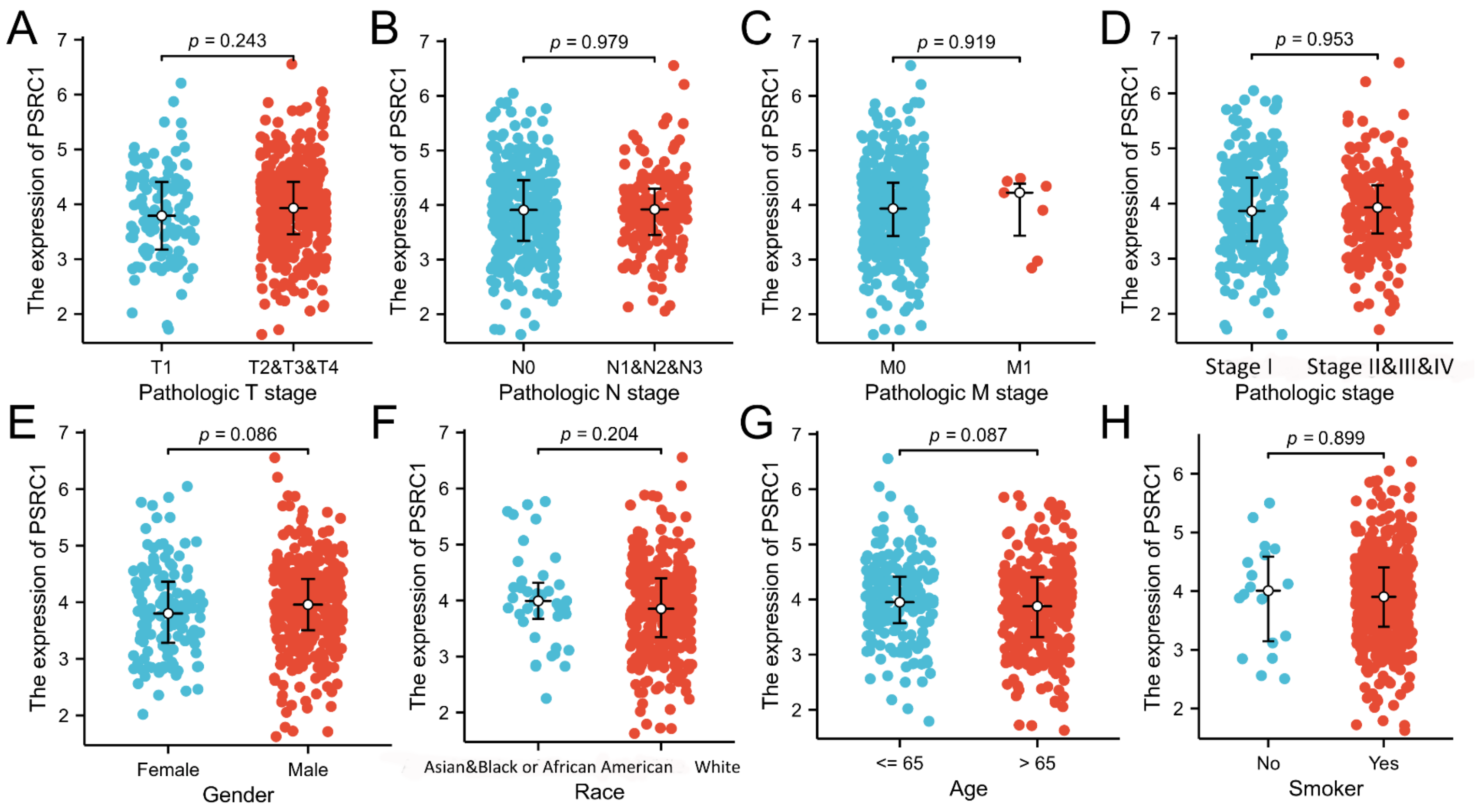

Figure S3. Kaplan-Meier survival curves comparing high and low PSRC1 expression in patients with LUSC in TCGA. (A) Overall survival. (B) progression free interval. Kaplan–Meier survival curves of 60 LUSC patients with high and low PSRC1-expressing tumors (C) Overall survival and (D) progression free interval.

PSRC1, Proline and serine rich coiled-coil 1; LUSC, lung squamous cell carcinoma; TCGA, The Cancer Genome Atlas.

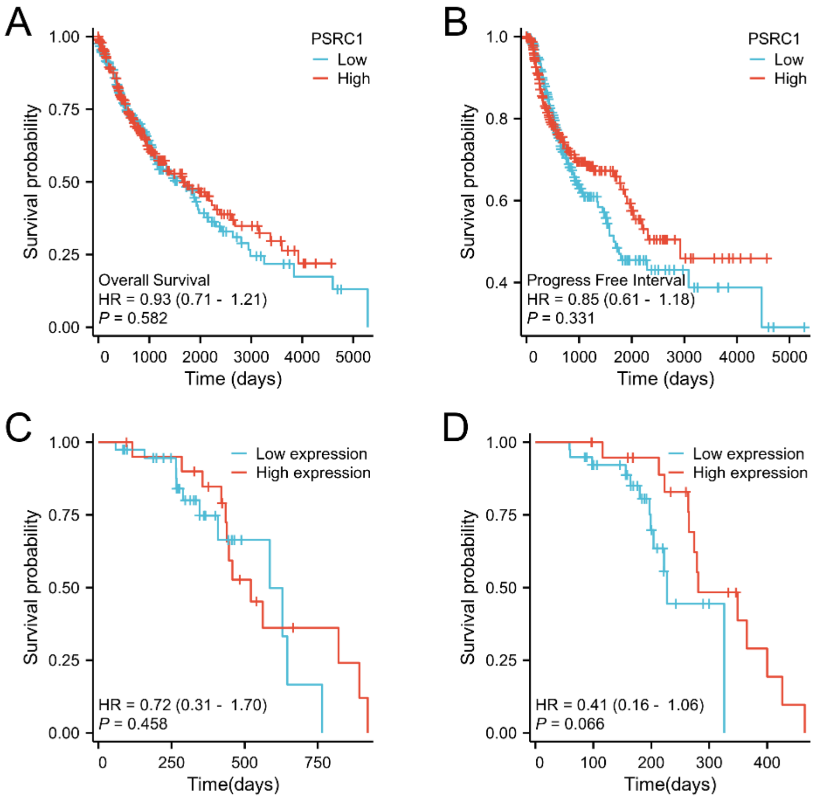

Figure S4. Kaplan–Meier curve for OS in LUAD. (A-F) Subgroup analysis for T1/T2 stage, N0 stage, M0 stage, pathological stage I/II/III stage, age > 65 years and female in LUAD.

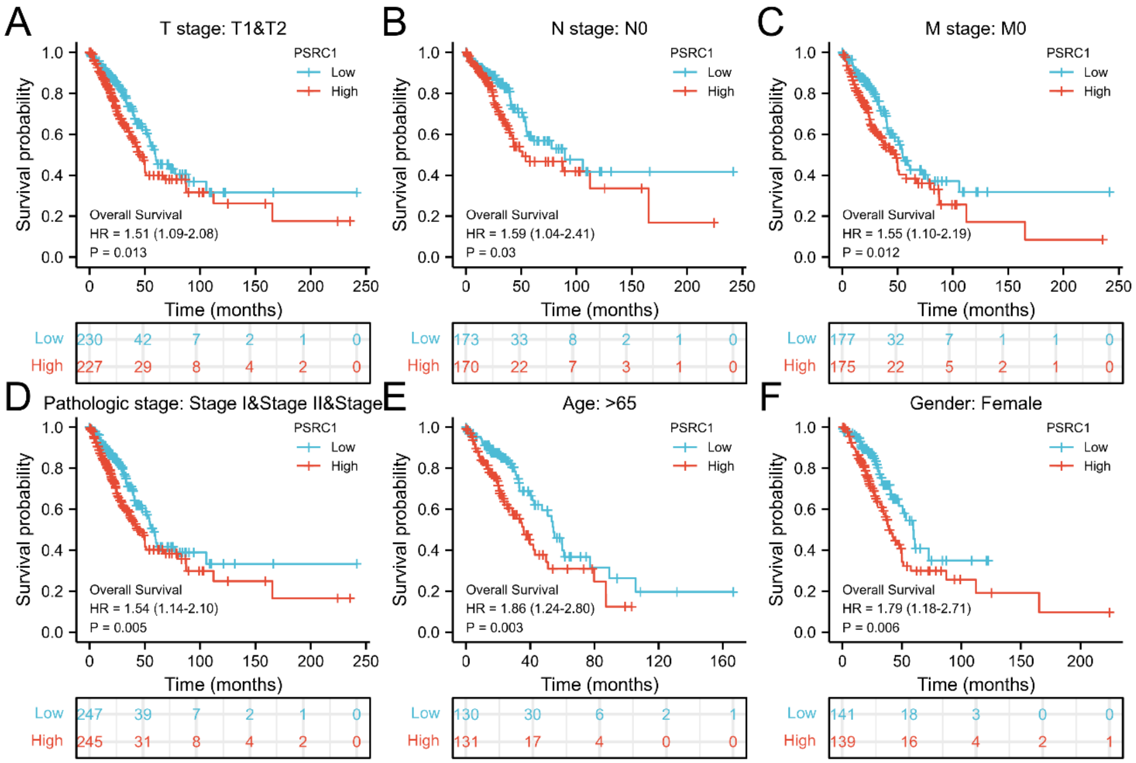

Figure S5. Calibration plot of the nomogram for predicting the likelihood of overall survival.

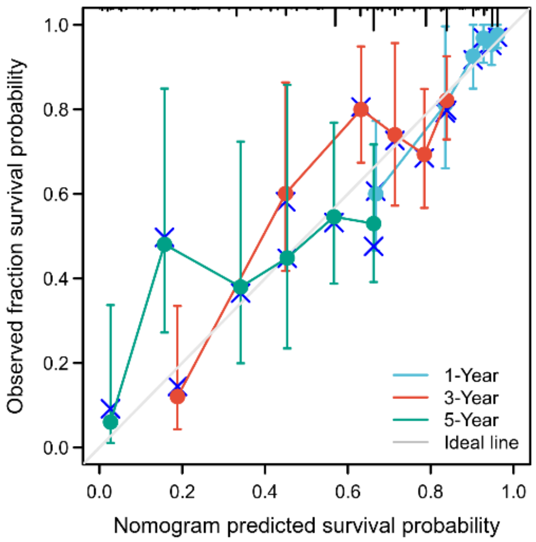

Figure S6. Heat map of module–trait correlations was obtained by WGCNA. Red represents positive correlations and blue represent negative correlations.

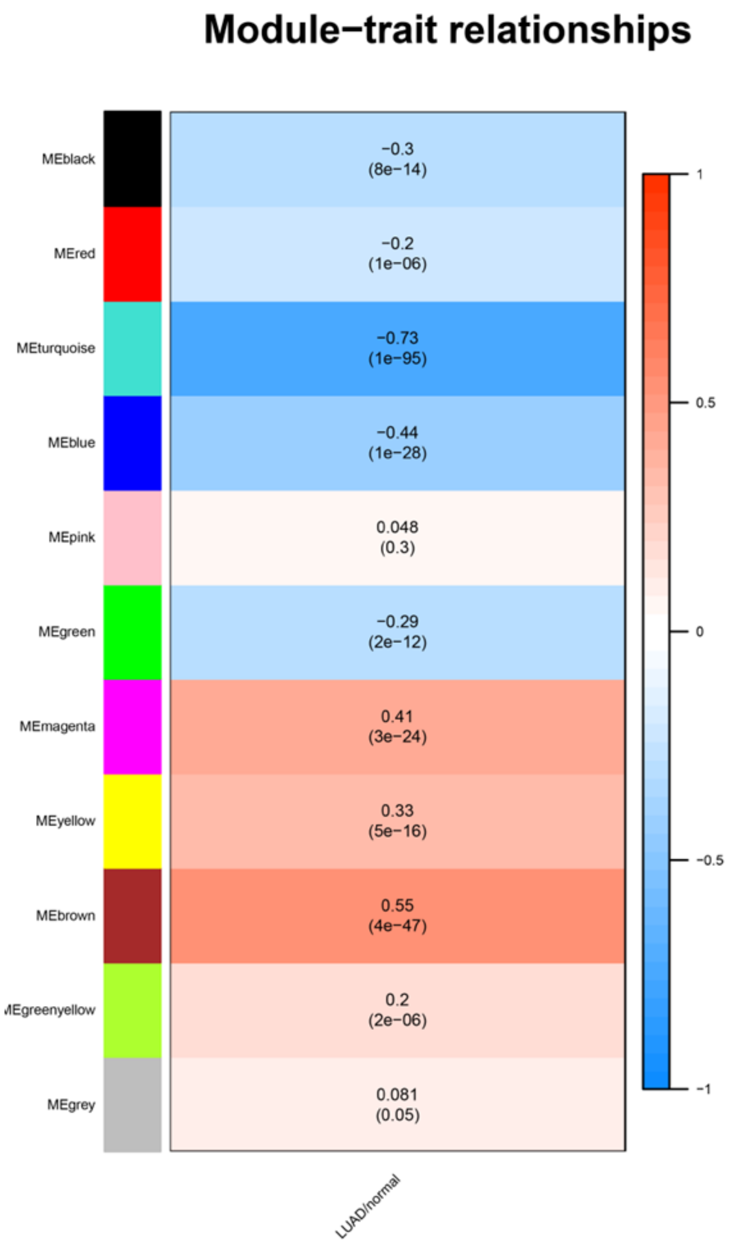
Module−trait relationships
MEblack
MEred
MEturquoise
MEblue
MEpink
MEgreen
MEmagenta
MEyellow
MEbrown
MEgreenyellow
MEgrey
−0.3
(8e−14)
−0.2
(1e−06)
−0.73
(1e−95)
−0.44
(1e−28)
0.048
(0.3)
−0.29
(2e−12)
0.41
(3e−24)
0.33
(5e−16)
0.55
(4e−47)
0.2
(2e−06)
0.081
(0.05)
1
0.5
0
−0.5
−1
LUAD/normal

Table S1. The raw data about LUAD and LUSC in TCGA database

RNAseq sample number	Status	Type	Residual tumor	Pathologic stage	M stage	N stage	T stage	Smoker	Vital status	Gender	Race	Age at initial pathologic diagnosis	OS
TCGA-05-4244-01A-01R-1107-07	Tumor	LUAD	RX	Stage IV	M1	N2	T2	Yes	Alive	MALE	Not Available	70	0
TCGA-05-4249-01A-01R-1107-07	Tumor	LUAD	R0	Stage IB	M0	N0	T2	Yes	Alive	MALE	Not Available	67	1523
TCGA-05-4250-01A-01R-1107-07	Tumor	LUAD	R2	Stage IIIA	M0	N1	T3	Yes	Dead	FEMALE	Not Available	79	121
TCGA-05-4382-01A-01R-1206-07	Tumor	LUAD	R0	Stage IB	M0	N0	T2	Yes	Alive	MALE	Not Available	68	607
TCGA-05-4384-01A-01R-1755-07	Tumor	LUAD	RX	Stage IIIA	M0	N2	T2	Yes	Alive	MALE	Not Available	66	426
TCGA-05-4389-01A-01R-1206-07	Tumor	LUAD	R0	Stage IA	M0	N0	T1	Yes	Alive	MALE	Not Available	70	1369

TCGA-05-4390-01A-02R-1755-07	Tumor	LUAD	R0	Stage IB	M0	N0	T2	Yes	Alive	FEMALE	Not Available	58	1126
TCGA-05-4395-01A-01R-1206-07	Tumor	LUAD	R0	Stage IIIB	M0	N2	T4	Yes	Dead	MALE	Not Available	76	0
TCGA-05-4396-01A-21R-1858-07	Tumor	LUAD	R0	Stage IIIB	M0	N1	T4	Yes	Dead	MALE	Not Available	76	303
TCGA-05-4397-01A-01R-1206-07	Tumor	LUAD	R0	Stage IIB	M0	N1	T2	Yes	Dead	MALE	Not Available	65	731
TCGA-05-4398-01A-01R-1206-07	Tumor	LUAD	R0	Stage IIIB	M0	N3	T4	Yes	Alive	FEMALE	Not Available	47	1431
TCGA-05-4402-01A-01R-1206-07	Tumor	LUAD	R2	Stage IV	M1	NX	T2	No	Dead	FEMALE	Not Available	57	244
TCGA-05-4403-01A-01R-1206-07	Tumor	LUAD	R0	Stage IB	M0	N0	T2	Yes	Alive	MALE	Not Available	76	578
TCGA-05-4405-01A-21R-1858-07	Tumor	LUAD	R0	Stage IB	M0	N0	T2	Yes	Alive	FEMALE	Not Available	74	610

TCGA-05-4410-01A-21R-1858-07	Tumor	LUAD	RX	Stage IB	M0	N0	T2	Yes	Alive	MALE	Not Available	62	0
TCGA-05-4415-01A-22R-1858-07	Tumor	LUAD	R0	Stage IIIB	M0	N2	T4	Yes	Dead	MALE	Not Available	57	91
TCGA-05-4417-01A-22R-1858-07	Tumor	LUAD	R0	Stage IB	M0	N0	T2	Yes	Alive	FEMALE	Not Available	51	455
TCGA-05-4418-01A-01R-1206-07	Tumor	LUAD	R0	Stage IIIA	M0	N2	T3	Yes	Dead	MALE	Not Available	69	274
TCGA-05-4420-01A-01R-1206-07	Tumor	LUAD	R0	Stage IB	M0	N0	T2	Yes	Alive	MALE	Not Available	41	912
TCGA-05-4422-01A-01R-1206-07	Tumor	LUAD	R0	Stage IB	M0	N0	T2	Yes	Alive	MALE	Not Available	68	365
TCGA-05-4424-01A-22R-1858-07	Tumor	LUAD	R1	Stage IIB	M0	N0	T3	Yes	Alive	MALE	Not Available	70	913
TCGA-05-4425-01A-01R-1755-07	Tumor	LUAD	R1	Stage IV	M1	N0	T2	Yes	Alive	FEMALE	Not Available	70	669

TCGA-05-4426-01A-01R-1206-07	Tumor	LUAD	R0	Stage IB	M0	N0	T2	Yes	Alive	MALE	Not Available	71	791
TCGA-05-4427-01A-21R-1858-07	Tumor	LUAD	R0	Stage IIB	M0	N1	T2	Yes	Alive	FEMALE	Not Available	65	791
TCGA-05-4430-01A-02R-1206-07	Tumor	LUAD	R0	Stage IB	M0	N0	T2	Yes	Alive	FEMALE	Not Available	59	761
TCGA-05-4432-01A-01R-1206-07	Tumor	LUAD	R0	Stage IIB	M0	N1	T2	Yes	Alive	MALE	Not Available	66	761
TCGA-05-4433-01A-22R-1858-07	Tumor	LUAD	R0	Stage IB	M0	N0	T2	Yes	Alive	MALE	Not Available	82	730
TCGA-05-4434-01A-01R-1206-07	Tumor	LUAD	R2	Stage IV	M1	N1	T4	Yes	Dead	FEMALE	Not Available	67	457
TCGA-05-5420-01A-01R-1628-07	Tumor	LUAD	R0	Stage IIIA	M0	N2	T2	Yes	Alive	MALE	Not Available	67	457
TCGA-05-5423-01A-01R-1628-07	Tumor	LUAD	R0	Stage IIB	M0	N1	T2	Yes	Alive	MALE	Not Available	65	151

TCGA-05-5425-01A-02R-1628-07	Tumor	LUAD	R0	Stage IIB	M0	N1	T2b	Yes	Alive	MALE	Not Available	68	882
TCGA-05-5428-01A-01R-1628-07	Tumor	LUAD	R0	Stage IIA	M0	N1	T1b	Yes	Alive	MALE	Not Available	57	670
TCGA-05-5429-01A-01R-1628-07	Tumor	LUAD	R0	Stage IIIA	M0	N2	T3	No	Alive	MALE	Not Available	60	275
TCGA-05-5715-01A-01R-1628-07	Tumor	LUAD	R0	Stage IB	M0	N0	T2a	No	Alive	FEMALE	Not Available	69	62
TCGA-35-3615-01A-01R-0946-07	Tumor	LUAD	R0	Stage IB	M0	N0	T2	No	Alive	MALE	WHITE	57	14
TCGA-35-4122-01A-01R-1107-07	Tumor	LUAD	R0	Stage IA	M0	N0	T1	Yes	Alive	MALE	WHITE	69	225
TCGA-35-4123-01A-01R-1107-07	Tumor	LUAD	R0	Stage IA	M0	N0	T1	Yes	Alive	MALE	WHITE	38	182
TCGA-35-5375-01A-01R-1628-07	Tumor	LUAD	R0	Stage IIIA	M0	N2	T2	Yes	Alive	MALE	WHITE	61	264

TCGA-38-4625-01A-01R-1206-07	Tumor	LUAD	R0	Stage IB	M0	N0	T2a	Yes	Alive	FEMALE	WHITE	66	2973
TCGA-38-4625-11A-01R-1758-07	Normal	LUAD	R0	Stage IB	M0	N0	T2a	Yes	Alive	FEMALE	WHITE	66	2973
TCGA-38-4626-01A-01R-1206-07	Tumor	LUAD	R0	Discrepancy	M0	N0	T2b	Yes	Alive	FEMALE	WHITE	57	3674
TCGA-38-4626-11A-01R-1758-07	Normal	LUAD	R0	Discrepancy	M0	N0	T2b	Yes	Alive	FEMALE	WHITE	57	3674
TCGA-38-4627-01A-01R-1206-07	Tumor	LUAD	R0	Stage IIA	M0	N1	T1b	Yes	Dead	FEMALE	WHITE	64	1147
TCGA-38-4627-11A-01R-1758-07	Normal	LUAD	R0	Stage IIA	M0	N1	T1b	Yes	Dead	FEMALE	WHITE	64	1147
TCGA-38-4628-01A-01R-1206-07	Tumor	LUAD	R0	Stage IIB	M0	N1	T2	No	Dead	FEMALE	WHITE	65	1492
TCGA-38-4629-01A-02R-1206-07	Tumor	LUAD	R0	Stage IIB	M0	N0	T3	Yes	Dead	MALE	WHITE	68	864

TCGA-38-4630-01A-01R-1206-07	Tumor	LUAD	R1	Stage IB	M0	N0	T2	No	Dead	FEMALE	WHITE	75	1073
TCGA-38-4631-01A-01R-1755-07	Tumor	LUAD	R0	Stage IB	M0	N0	T2	Yes	Dead	FEMALE	WHITE	72	354
TCGA-38-4632-01A-01R-1755-07	Tumor	LUAD	R0	Stage IV	M1	N1	T2	Yes	Dead	MALE	BLACK OR AFRICAN AMERICAN	42	1357
TCGA-38-4632-11A-01R-1755-07	Normal	LUAD	R0	Stage IV	M1	N1	T2	Yes	Dead	MALE	BLACK OR AFRICAN AMERICAN	42	1357
TCGA-38-6178-01A-11R-1755-07	Tumor	LUAD	R1	Stage IIIA	Not Available	N2	T2b	No	Alive	FEMALE	WHITE	70	448
TCGA-38-7271-01A-11R-2039-07	Tumor	LUAD	R0	Stage IA	M0	N0	T1	Yes	Dead	FEMALE	WHITE	72	800

TCGA-38-A44F-01A-11R- A24H-07	Tumor	LUAD	R0	Stage IB	M0	N0	T2a	Yes	Alive	MALE	WHITE	80	133
TCGA-44-2655-01A-01R-0946-07	Tumor	LUAD	R0	Stage IA	M0	N0	T1	Yes	Alive	FEMALE	WHITE	65	1324
TCGA-44-2655-11A-01R-1758-07	Normal	LUAD	R0	Stage IA	M0	N0	T1	Yes	Alive	FEMALE	WHITE	65	1324
TCGA-44-2656-01A-02R-0946-07	Tumor	LUAD	Not Available	Stage IB	M0	N0	T2	Yes	Alive	MALE	WHITE	59	1429
TCGA-44-2656-01A-02R- A278-07	Tumor	LUAD	Not Available	Stage IB	M0	N0	T2	Yes	Alive	MALE	WHITE	59	1429
TCGA-44-2656-01B-06R- A277-07	Tumor	LUAD	Not Available	Stage IB	M0	N0	T2	Yes	Alive	MALE	WHITE	59	1429
TCGA-44-2657-01A-01R-1107-07	Tumor	LUAD	R0	Stage IB	M0	NX	T2	Yes	Alive	FEMALE	WHITE	74	1351
TCGA-44-2657-11A-01R-1758-07	Normal	LUAD	R0	Stage IB	M0	NX	T2	Yes	Alive	FEMALE	WHITE	74	1351

TCGA-44-2659-01A-01R-0946-07	Tumor	LUAD	R0	Stage IIB	M0	N1	T1	Yes	Alive	FEMALE	WHITE	65	1367
TCGA-44-2661-01A-01R-1107-07	Tumor	LUAD	R0	Stage IA	M0	N0	T1	No	Alive	FEMALE	WHITE	69	1159
TCGA-44-2661-11A-01R-1758-07	Normal	LUAD	R0	Stage IA	M0	N0	T1	No	Alive	FEMALE	WHITE	69	1159
TCGA-44-2662-01A-01R-0946-07	Tumor	LUAD	R0	Stage IB	M0	N0	T2	Yes	Alive	MALE	WHITE	65	1280
TCGA-44-2662-01A-01R- A278-07	Tumor	LUAD	R0	Stage IB	M0	N0	T2	Yes	Alive	MALE	WHITE	65	1280
TCGA-44-2662-01B-02R- A277-07	Tumor	LUAD	R0	Stage IB	M0	N0	T2	Yes	Alive	MALE	WHITE	65	1280
TCGA-44-2662-11A-01R-1758-07	Normal	LUAD	R0	Stage IB	M0	N0	T2	Yes	Alive	MALE	WHITE	65	1280
TCGA-44-2665-01A-01R-0946-07	Tumor	LUAD	R0	Stage IIB	M0	N1	T2	No	Alive	FEMALE	WHITE	55	1301

TCGA-44-2665-11A-01R-1758-07	Normal	LUAD	R0	Stage IIB	M0	N1	T2	No	Alive	FEMALE	WHITE	55	1301
TCGA-44-2666-01A-01R-0946-07	Tumor	LUAD	R0	Stage IB	M0	N0	T2	Yes	Dead	MALE	WHITE	43	97
TCGA-44-2666-01A-01R- A278-07	Tumor	LUAD	R0	Stage IB	M0	N0	T2	Yes	Dead	MALE	WHITE	43	97
TCGA-44-2666-01B-02R- A277-07	Tumor	LUAD	R0	Stage IB	M0	N0	T2	Yes	Dead	MALE	WHITE	43	97
TCGA-44-2668-01A-01R-0946-07	Tumor	LUAD	R0	Stage IB	M0	N0	T2	Yes	Alive	MALE	WHITE	51	761
TCGA-44-2668-01A-01R- A278-07	Tumor	LUAD	R0	Stage IB	M0	N0	T2	Yes	Alive	MALE	WHITE	51	761
TCGA-44-2668-01B-02R- A277-07	Tumor	LUAD	R0	Stage IB	M0	N0	T2	Yes	Alive	MALE	WHITE	51	761
TCGA-44-2668-11A-01R-1758-07	Normal	LUAD	R0	Stage IB	M0	N0	T2	Yes	Alive	MALE	WHITE	51	761

TCGA-44-3396-01A-01R-1206-07	Tumor	LUAD	R0	Stage IIIA	M0	N2	T2	Yes	Alive	FEMALE	WHITE	74	1130
TCGA-44-3396-11A-01R-1758-07	Normal	LUAD	R0	Stage IIIA	M0	N2	T2	Yes	Alive	FEMALE	WHITE	74	1130
TCGA-44-3398-01A-01R-1107-07	Tumor	LUAD	R0	Stage IA	M0	N0	T1b	Yes	Alive	FEMALE	WHITE	77	1163
TCGA-44-3398-11B-01R-1758-07	Normal	LUAD	R0	Stage IA	M0	N0	T1b	Yes	Alive	FEMALE	WHITE	77	1163
TCGA-44-3917-01A-01R- A278-07	Tumor	LUAD	R0	Stage IB	M0	N0	T2	Yes	Alive	FEMALE	WHITE	33	1183
TCGA-44-3917-01B-02R- A277-07	Tumor	LUAD	R0	Stage IB	M0	N0	T2	Yes	Alive	FEMALE	WHITE	33	1183
TCGA-44-3918-01A-01R-1107-07	Tumor	LUAD	R0	Stage IA	M0	N0	T1	Yes	Alive	FEMALE	WHITE	60	1036
TCGA-44-3918-01A-01R- A278-07	Tumor	LUAD	R0	Stage IA	M0	N0	T1	Yes	Alive	FEMALE	WHITE	60	1036

TCGA-44-3918-01B-02R- A277-07	Tumor	LUAD	R0	Stage IA	M0	N0	T1	Yes	Alive	FEMALE	WHITE	60	1036
TCGA-44-3919-01A-02R-1107-07	Tumor	LUAD	R0	Stage IA	M0	N0	T1	No	Alive	FEMALE	WHITE	71	1026
TCGA-44-4112-01A-01R-1107-07	Tumor	LUAD	R0	Stage IB	M0	N0	T2a	Yes	Alive	FEMALE	WHITE	60	808
TCGA-44-4112-01B-06R- A277-07	Tumor	LUAD	R0	Stage IB	M0	N0	T2a	Yes	Alive	FEMALE	WHITE	60	808
TCGA-44-5643-01A-01R-1628-07	Tumor	LUAD	Not Available	Stage IIIA	M0	N2	T2b	Yes	Alive	MALE	BLACK OR AFRICAN AMERICAN	53	1013
TCGA-44-5644-01A-21R-2039-07	Tumor	LUAD	Not Available	Stage IB	Not Available	N0	T2a	Yes	Alive	FEMALE	WHITE	51	863

TCGA-44-5645-01A-01R-1628-07	Tumor	LUAD	Not Available	Stage IA	Not Available	NX	T1	Yes	Alive	FEMALE	BLACK OR AFRICAN AMERICAN	61	852
TCGA-44-5645-01A-01R- A278-07	Tumor	LUAD	Not Available	Stage IA	Not Available	NX	T1	Yes	Alive	FEMALE	BLACK OR AFRICAN AMERICAN	61	852
TCGA-44-5645-01B-04R- A277-07	Tumor	LUAD	Not Available	Stage IA	Not Available	NX	T1	Yes	Alive	FEMALE	BLACK OR AFRICAN AMERICAN	61	852
TCGA-44-5645-11A-01R-1628-07	Normal	LUAD	Not Available	Stage IA	Not Available	NX	T1	Yes	Alive	FEMALE	BLACK OR AFRICAN AMERICAN	61	852
TCGA-44-6144-11A-01R-1755-07	Normal	LUAD	RX	Stage IA	M0	N0	T1a	Yes	Alive	MALE	WHITE	58	723

TCGA-44-6145-01A-11R-1755-07	Tumor	LUAD	Not Available	Stage IA	M0	N0	T1	Yes	Alive	FEMALE	WHITE	62	595
TCGA-44-6145-11A-01R-1858-07	Normal	LUAD	Not Available	Stage IA	M0	N0	T1	Yes	Alive	FEMALE	WHITE	62	595
TCGA-44-6146-01A-11R-1755-07	Tumor	LUAD	Not Available	Stage IIB	M0	N0	T3	Yes	Alive	MALE	WHITE	64	728
TCGA-44-6146-01A-11R- A278-07	Tumor	LUAD	Not Available	Stage IIB	M0	N0	T3	Yes	Alive	MALE	WHITE	64	728
TCGA-44-6146-01B-04R- A277-07	Tumor	LUAD	Not Available	Stage IIB	M0	N0	T3	Yes	Alive	MALE	WHITE	64	728
TCGA-44-6146-11A-01R-1858-07	Normal	LUAD	Not Available	Stage IIB	M0	N0	T3	Yes	Alive	MALE	WHITE	64	728
TCGA-44-6147-01A-11R-1755-07	Tumor	LUAD	Not Available	Stage IA	M0	NX	T1b	Yes	Alive	FEMALE	WHITE	67	845
TCGA-44-6147-01A-11R- A278-07	Tumor	LUAD	Not Available	Stage IA	M0	NX	T1b	Yes	Alive	FEMALE	WHITE	67	845

TCGA-44-6147-01B-06R- A277-07	Tumor	LUAD	Not Available	Stage IA	M0	NX	T1b	Yes	Alive	FEMALE	WHITE	67	845
TCGA-44-6147-11A-01R-1858-07	Normal	LUAD	Not Available	Stage IA	M0	NX	T1b	Yes	Alive	FEMALE	WHITE	67	845
TCGA-44-6148-01A-11R-1755-07	Tumor	LUAD	RX	Stage IA	M0	N0	T1b	Yes	Alive	MALE	WHITE	60	704
TCGA-44-6148-11A-01R-1858-07	Normal	LUAD	RX	Stage IA	M0	N0	T1b	Yes	Alive	MALE	WHITE	60	704
TCGA-44-6774-01A-21R-1858-07	Tumor	LUAD	Not Available	Stage IIIA	M0	N2	T1	Yes	Alive	FEMALE	WHITE	56	658
TCGA-44-6775-01A-11R-1858-07	Tumor	LUAD	RX	Stage IB	MX	N0	T2a	Yes	Alive	FEMALE	WHITE	72	705
TCGA-44-6775-01A-11R- A278-07	Tumor	LUAD	RX	Stage IB	MX	N0	T2a	Yes	Alive	FEMALE	WHITE	72	705
TCGA-44-6775-01C-02R- A277-07	Tumor	LUAD	RX	Stage IB	MX	N0	T2a	Yes	Alive	FEMALE	WHITE	72	705

TCGA-44-6776-01A-11R-1858-07	Tumor	LUAD	Not Available	Stage IA	MX	N0	T1	Yes	Alive	FEMALE	WHITE	60	2616
TCGA-44-6776-11A-01R-1858-07	Normal	LUAD	Not Available	Stage IA	MX	N0	T1	Yes	Alive	FEMALE	WHITE	60	2616
TCGA-44-6777-01A-11R-1858-07	Tumor	LUAD	Not Available	Stage IB	MX	NX	T2	Yes	Dead	FEMALE	WHITE	85	987
TCGA-44-6777-11A-01R-1858-07	Normal	LUAD	Not Available	Stage IB	MX	NX	T2	Yes	Dead	FEMALE	WHITE	85	987
TCGA-44-6778-01A-11R-1858-07	Tumor	LUAD	Not Available	Stage IA	MX	N0	T1	Yes	Alive	MALE	BLACK OR AFRICAN AMERICAN	59	1864
TCGA-44-6778-11A-01R-1858-07	Normal	LUAD	Not Available	Stage IA	MX	N0	T1	Yes	Alive	MALE	BLACK OR AFRICAN AMERICAN	59	1864
TCGA-44-6779-01A-11R-1858-07	Tumor	LUAD	Not Available	Stage IIB	MX	N1	T2	Yes	Dead	FEMALE	WHITE	50	500

TCGA-44-7659-01A-11R-2066-07	Tumor	LUAD	Not Available	Stage IA	MX	N0	T1b	Yes	Alive	MALE	WHITE	70	691
TCGA-44-7660-01A-11R-2066-07	Tumor	LUAD	Not Available	Stage IB	MX	N0	T2	Yes	Alive	MALE	WHITE	72	592
TCGA-44-7661-01A-11R-2066-07	Tumor	LUAD	Not Available	Stage IB	M0	N0	T2a	Yes	Alive	FEMALE	WHITE	69	557
TCGA-44-7662-01A-11R-2066-07	Tumor	LUAD	Not Available	Stage IB	MX	N0	T2a	Yes	Alive	MALE	WHITE	61	218
TCGA-44-7667-01A-31R-2066-07	Tumor	LUAD	Not Available	Stage IIB	MX	N0	T3	Yes	Alive	FEMALE	WHITE	49	1097
TCGA-44-7669-01A-21R-2066-07	Tumor	LUAD	Not Available	Stage IIA	MX	N1	T1b	Yes	Dead	MALE	BLACK OR AFRICAN AMERICAN	59	574
TCGA-44-7670-01A-11R-2066-07	Tumor	LUAD	Not Available	Stage IIA	M0	N1	T1b	Yes	Alive	FEMALE	WHITE	47	882

TCGA-44-7671-01A-11R-2066-07	Tumor	LUAD	Not Available	Stage IB	M0	N0	T2a	Yes	Alive	MALE	BLACK OR AFRICAN AMERICAN	64	889
TCGA-44-7672-01A-11R-2066-07	Tumor	LUAD	Not Available	Stage IA	M0	N0	T1b	Yes	Alive	FEMALE	WHITE	52	719
TCGA-44-8117-01A-11R-2241-07	Tumor	LUAD	Not Available	Stage IB	M0	N0	T2a	Yes	Alive	FEMALE	WHITE	54	385
TCGA-44-8119-01A-11R-2241-07	Tumor	LUAD	Not Available	Stage IIB	M0	N0	T3	Yes	Alive	MALE	WHITE	73	285
TCGA-44-8120-01A-11R-2241-07	Tumor	LUAD	Not Available	Stage IB	M0	N0	T2a	Yes	Alive	MALE	BLACK OR AFRICAN AMERICAN	58	260

TCGA-44-A479-01A-31R- A24H-07	Tumor	LUAD	R0	Stage IB	MX	N0	T2	Yes	Alive	FEMALE	BLACK OR AFRICAN AMERICAN	73	486
TCGA-44-A47A-01A-21R-A24H-07	Tumor	LUAD	R0	Stage IB	MX	N0	T2a	Yes	Alive	FEMALE	WHITE	78	466
TCGA-44-A47B-01A-11R- A24H-07	Tumor	LUAD	R0	Stage IB	M0	N0	T2a	Yes	Alive	MALE	WHITE	79	287
TCGA-44-A47G-01A-21R-A24H-07	Tumor	LUAD	R0	Stage IA	M0	N0	T1	Yes	Alive	FEMALE	WHITE	73	351
TCGA-44-A4SS-01A-11R-A24X-07	Tumor	LUAD	Not Available	Stage IA	M0	N0	T1b	Yes	Alive	MALE	WHITE	73	415
TCGA-44-A4SU-01A-11R-A24X-07	Tumor	LUAD	R0	Stage IA	MX	N0	T1a	Yes	Alive	FEMALE	WHITE	67	409

TCGA-49-4486-01A-01R-1206-07	Tumor	LUAD	R0	Stage IA	M0	N0	T1	Yes	Dead	MALE	WHITE	72	2318
TCGA-49-4487-01A-21R-1858-07	Tumor	LUAD	R0	Stage IA	M0	N0	T1	Yes	Dead	FEMALE	WHITE	72	855
TCGA-49-4488-01A-01R-1755-07	Tumor	LUAD	R0	Stage IA	MX	N0	T1	Yes	Dead	FEMALE	WHITE	74	869
TCGA-49-4490-01A-21R-1858-07	Tumor	LUAD	Not Available	Stage IIIA	M0	N2	T3	Yes	Dead	FEMALE	WHITE	45	385
TCGA-49-4490-11A-01R-1858-07	Normal	LUAD	Not Available	Stage IIIA	M0	N2	T3	Yes	Dead	FEMALE	WHITE	45	385
TCGA-49-4494-01A-01R-1206-07	Tumor	LUAD	R0	Stage IIIA	M0	N2	T3	Yes	Dead	MALE	WHITE	77	1081
TCGA-49-4501-01A-01R-1206-07	Tumor	LUAD	R0	Stage IB	M0	N0	T2	No	Dead	FEMALE	WHITE	67	1421
TCGA-49-4505-01A-01R-1206-07	Tumor	LUAD	R0	Stage IIB	M0	N1	T2	Yes	Dead	FEMALE	WHITE	61	428

TCGA-49-4506-01A-01R-1206-07	Tumor	LUAD	R1	Stage IIB	M0	N1	T2	Yes	Dead	FEMALE	WHITE	68	999
TCGA-49-4507-01A-01R-1206-07	Tumor	LUAD	R0	Stage IIIA	M0	N1	T3	Yes	Dead	FEMALE	WHITE	73	268
TCGA-49-4510-01A-01R-1206-07	Tumor	LUAD	R0	Stage IIB	M0	N1	T2	Yes	Dead	FEMALE	BLACK OR AFRICAN AMERICAN	51	896
TCGA-49-4512-01A-21R-1858-07	Tumor	LUAD	R1	Stage IIIA	MX	N2	T2	No	Dead	FEMALE	WHITE	69	905
TCGA-49-4512-11A-01R-1858-07	Normal	LUAD	R1	Stage IIIA	MX	N2	T2	No	Dead	FEMALE	WHITE	69	905
TCGA-49-4514-01A-21R-1858-07	Tumor	LUAD	R0	Stage IA	M0	N0	T1	Yes	Alive	FEMALE	WHITE	79	1700
TCGA-49-6742-01A-11R-1858-07	Tumor	LUAD	R0	Stage IIA	M0	N1	T2a	Yes	Alive	MALE	WHITE	70	488

TCGA-49-6742-11A-01R-1858-07	Normal	LUAD	R0	Stage IIA	M0	N1	T2a	Yes	Alive	MALE	WHITE	70	488
TCGA-49-6743-01A-11R-1858-07	Tumor	LUAD	Not Available	Stage IIIA	MX	N2	T1	Yes	Alive	FEMALE	WHITE	81	1621
TCGA-49-6743-11A-01R-1858-07	Normal	LUAD	Not Available	Stage IIIA	MX	N2	T1	Yes	Alive	FEMALE	WHITE	81	1621
TCGA-49-6744-01A-11R-1858-07	Tumor	LUAD	R0	Stage IIA	MX	N1	T2a	Yes	Alive	FEMALE	WHITE	64	1683
TCGA-49-6744-11A-01R-1858-07	Normal	LUAD	R0	Stage IIA	MX	N1	T2a	Yes	Alive	FEMALE	WHITE	64	1683
TCGA-49-6745-01A-11R-1858-07	Tumor	LUAD	Not Available	Stage IIIA	M0	N2	T2a	Yes	Alive	MALE	WHITE	82	522
TCGA-49-6745-11A-01R-1858-07	Normal	LUAD	Not Available	Stage IIIA	M0	N2	T2a	Yes	Alive	MALE	WHITE	82	522
TCGA-49-6761-01A-31R-1949-07	Tumor	LUAD	R0	Stage IIIA	MX	N2	T1	Yes	Alive	FEMALE	WHITE	68	354

TCGA-49-6761-11A-01R-1949-07	Normal	LUAD	R0	Stage IIIA	MX	N2	T1	Yes	Alive	FEMALE	WHITE	68	354
TCGA-49-6767-01A-11R-1858-07	Tumor	LUAD	R0	Stage IIB	MX	N0	T3	Yes	Alive	FEMALE	WHITE	46	677
TCGA-49-AAQV-01A-11R-A39D-07	Tumor	LUAD	R0	Stage II	MX	N1	T1	No	Dead	FEMALE	BLACK OR AFRICAN AMERICAN	63	677
TCGA-49-AAR0-01A-21R-A39D-07	Tumor	LUAD	R0	Stage IA	MX	N0	T1	Yes	Alive	MALE	BLACK OR AFRICAN AMERICAN	57	4765
TCGA-49-AAR2-01A-11R-A39D-07	Tumor	LUAD	R0	Stage IB	MX	N0	T2	Yes	Alive	MALE	BLACK OR AFRICAN AMERICAN	64	2224
TCGA-49-AAR3-01A-11R-A41B-07	Tumor	LUAD	R0	Stage IIB	MX	N1	T2	Unknown	Alive	MALE	BLACK OR AFRICAN AMERICAN	69	1893

TCGA-49-AAR4-01A-12R-A41B-07	Tumor	LUAD	R0	Stage IIIA	MX	N2	T2	Yes	Dead	MALE	BLACK OR AFRICAN AMERICAN	51	879
TCGA-49-AAR9-01A-21R-A41B-07	Tumor	LUAD	R0	Stage IIB	MX	N0	T3	Yes	Dead	MALE	BLACK OR AFRICAN AMERICAN	61	260
TCGA-49-AARE-01A-11R-A41B-07	Tumor	LUAD	R0	Stage IA	MX	N0	T1	Yes	Dead	FEMALE	BLACK OR AFRICAN AMERICAN	51	1229
TCGA-49-AARN-01A-21R-A41B-07	Tumor	LUAD	R0	Stage IA	MX	N0	T1	Yes	Dead	FEMALE	BLACK OR AFRICAN AMERICAN	56	1135
TCGA-49-AARO-01A-12R-A41B-07	Tumor	LUAD	R0	Stage IA	MX	N0	T1a	Yes	Alive	FEMALE	BLACK OR AFRICAN AMERICAN	39	3759

TCGA-49-AARQ-01A-11R-A41B-07	Tumor	LUAD	R0	Stage I	MX	N0	T2	Yes	Alive	FEMALE	BLACK OR AFRICAN AMERICAN	41	6732
TCGA-49-AARR-01A-11R-A41B-07	Tumor	LUAD	R0	Stage IA	MX	N0	T1	Yes	Alive	MALE	BLACK OR AFRICAN AMERICAN	68	4992
TCGA-4B-A93V-01A-11R- A39D-07	Tumor	LUAD	R0	Stage IA	M0	N0	T1b	Yes	Alive	FEMALE	BLACK OR AFRICAN AMERICAN	52	300
TCGA-50-5044-01A-21R-1858-07	Tumor	LUAD	Not Available	Stage IIIB	M0	N1	T4	Not Available	Dead	FEMALE	WHITE	72	624
TCGA-50-5045-01A-01R-1628-07	Tumor	LUAD	Not Available	Discrepancy	M0	N1	T2	Not Available	Dead	FEMALE	BLACK OR AFRICAN AMERICAN	57	2174

TCGA-50-5049-01A-01R-1628-07	Tumor	LUAD	Not Available	Stage IA	M0	N0	T2	Not Available	Alive	MALE	WHITE	70	3094
TCGA-50-5051-01A-21R-1858-07	Tumor	LUAD	R0	Stage IIIA	M0	N2	T2	Yes	Alive	FEMALE	WHITE	42	478
TCGA-50-5055-01A-01R-1628-07	Tumor	LUAD	R0	Stage IIA	M0	N1	T1	Not Available	Alive	FEMALE	WHITE	79	1830
TCGA-50-5066-01A-01R-1628-07	Tumor	LUAD	R0	Stage IB	M0	N0	T2	No	Alive	MALE	BLACK OR AFRICAN AMERICAN	72	1442
TCGA-50-5066-02A-11R-2090-07	Tumor	LUAD	R0	Stage IB	M0	N0	T2	No	Alive	MALE	BLACK OR AFRICAN AMERICAN	72	1442

TCGA-50-5068-01A-01R-1628-07	Tumor	LUAD	Not Available	Stage IIB	MX	N1	T2	Not Available	Dead	FEMALE	WHITE	59	1499
TCGA-50-5072-01A-21R-1858-07	Tumor	LUAD	Not Available	Stage IIIA	M0	N2	T2	Yes	Alive	MALE	WHITE	74	250
TCGA-50-5930-01A-11R-1755-07	Tumor	LUAD	Not Available	Stage IIIA	M0	N2	T2	Not Available	Dead	MALE	WHITE	47	282
TCGA-50-5930-11A-01R-1755-07	Normal	LUAD	Not Available	Stage IIIA	M0	N2	T2	Not Available	Dead	MALE	WHITE	47	282
TCGA-50-5931-01A-11R-1755-07	Tumor	LUAD	R0	Stage IB	M0	N0	T2	Yes	Dead	FEMALE	WHITE	75	434
TCGA-50-5931-11A-01R-1858-07	Normal	LUAD	R0	Stage IB	M0	N0	T2	Yes	Dead	FEMALE	WHITE	75	434
TCGA-50-5932-01A-11R-1755-07	Tumor	LUAD	Not Available	Stage IIB	M0	N1	T2	Not Available	Dead	MALE	WHITE	75	1235

TCGA-50-5932-11A-01R-1755-07	Normal	LUAD	Not Available	Stage IIB	M0	N1	T2	Not Available	Dead	MALE	WHITE	75	1235
TCGA-50-5933-01A-11R-1755-07	Tumor	LUAD	Not Available	Stage IIIB	M0	N2	T4	Not Available	Dead	MALE	WHITE	72	2393
TCGA-50-5933-11A-01R-1755-07	Normal	LUAD	Not Available	Stage IIIB	M0	N2	T4	Not Available	Dead	MALE	WHITE	72	2393
TCGA-50-5935-01A-11R-1755-07	Tumor	LUAD	Not Available	Stage IA	M0	N0	T1	Not Available	Dead	FEMALE	WHITE	86	653
TCGA-50-5935-11A-01R-1858-07	Normal	LUAD	Not Available	Stage IA	M0	N0	T1	Not Available	Dead	FEMALE	WHITE	86	653
TCGA-50-5936-01A-11R-1628-07	Tumor	LUAD	Not Available	Stage IIIA	M0	N2	T2	Not Available	Dead	MALE	WHITE	58	257
TCGA-50-5936-11A-01R-1628-07	Normal	LUAD	Not Available	Stage IIIA	M0	N2	T2	Not Available	Dead	MALE	WHITE	58	257
TCGA-50-5939-01A-11R-1628-07	Tumor	LUAD	Not Available	Stage IB	M0	N0	T2	Yes	Dead	MALE	WHITE	85	460

TCGA-50-5939-11A-01R-1628-07	Normal	LUAD	Not Available	Stage IB	M0	N0	T2	Yes	Dead	MALE	WHITE	85	460
TCGA-50-5941-01A-11R-1755-07	Tumor	LUAD	Not Available	Stage IIIA	M0	N2	T2a	Yes	Alive	FEMALE	WHITE	55	1474
TCGA-50-5942-01A-21R-1755-07	Tumor	LUAD	R0	Stage IA	M0	N0	T1	Yes	Alive	FEMALE	WHITE	67	1847
TCGA-50-5944-01A-11R-1755-07	Tumor	LUAD	R0	Stage IA	M0	N0	T1	Not Available	Alive	FEMALE	WHITE	69	1750
TCGA-50-5946-01A-11R-1755-07	Tumor	LUAD	Not Available	Stage IA	MX	N0	T1	Yes	Alive	MALE	WHITE	62	1617
TCGA-50-5946-02A-11R-2090-07	Tumor	LUAD	Not Available	Stage IA	MX	N0	T1	Yes	Alive	MALE	WHITE	62	1617
TCGA-50-6590-01A-12R-1858-07	Tumor	LUAD	Not Available	Stage IB	M0	N0	T2	Yes	Dead	FEMALE	WHITE	72	1288
TCGA-50-6591-01A-11R-1755-07	Tumor	LUAD	Not Available	Stage IV	M1	N0	T2	No	Dead	FEMALE	WHITE	63	119

TCGA-50-6592-01A-11R-1755-07	Tumor	LUAD	Not Available	Stage IB	M0	N0	T2	Yes	Dead	FEMALE	WHITE	71	777
TCGA-50-6593-01A-11R-1755-07	Tumor	LUAD	R0	Stage IIIA	M0	N2	T1	Yes	Dead	FEMALE	WHITE	49	336
TCGA-50-6594-01A-11R-1755-07	Tumor	LUAD	Not Available	Stage IIIA	M0	N2	T3	Yes	Dead	FEMALE	BLACK OR AFRICAN AMERICAN	79	370
TCGA-50-6595-01A-12R-1858-07	Tumor	LUAD	Not Available	Stage IIIA	M0	N2	T2	Yes	Dead	FEMALE	WHITE	74	189
TCGA-50-6595-11A-01R-1858-07	Normal	LUAD	Not Available	Stage IIIA	M0	N2	T2	Yes	Dead	FEMALE	WHITE	74	189
TCGA-50-6597-01A-11R-1858-07	Tumor	LUAD	R0	Stage IB	M0	N0	T2	No	Alive	FEMALE	WHITE	79	1268
TCGA-50-6673-01A-11R-1949-07	Tumor	LUAD	R0	Stage I	M0	N0	T1	No	Dead	FEMALE	WHITE	84	22

TCGA-50-7109-01A-11R-2039-07	Tumor	LUAD	Not Available	Stage IA	M0	N0	T1	Yes	Alive	MALE	WHITE	60	308
TCGA-50-8457-01A-11R-2326-07	Tumor	LUAD	Not Available	Stage IA	M0	N0	T1a	Yes	Alive	FEMALE	BLACK OR AFRICAN AMERICAN	63	1125
TCGA-50-8459-01A-11R-2326-07	Tumor	LUAD	Not Available	Stage IIB	M0	N0	T3	Yes	Alive	MALE	WHITE	68	1119
TCGA-50-8460-01A-11R-2326-07	Tumor	LUAD	R0	Stage IA	M0	N0	T1a	Unknown	Alive	MALE	WHITE	74	829
TCGA-53-7624-01A-11R-2066-07	Tumor	LUAD	R0	Stage IV	M1	N0	T2	Yes	Dead	FEMALE	WHITE	40	1043
TCGA-53-7626-01A-12R-2066-07	Tumor	LUAD	R0	Stage IIA	M0	N1	T1	Yes	Dead	FEMALE	WHITE	76	929
TCGA-53-7813-01A-11R-2170-07	Tumor	LUAD	Not Available	Stage IIIB	M0	N0	T4	Yes	Alive	FEMALE	WHITE	51	424

TCGA-53-A4EZ-01A-12R-A24X-07	Tumor	LUAD	RX	Stage IIA	MX	N1	T2a	Yes	Alive	MALE	WHITE	63	1071
TCGA-55-1592-01A-01R-0946-07	Tumor	LUAD	R0	Stage IA	M0	N0	T2	Yes	Dead	MALE	WHITE	65	701
TCGA-55-1594-01A-01R-0946-07	Tumor	LUAD	R0	Stage IIIA	M0	N2	T2	Yes	Alive	MALE	WHITE	68	1178
TCGA-55-1596-01A-01R-0946-07	Tumor	LUAD	R0	Stage IIB	M0	N1	T2	Yes	Alive	MALE	ASIAN	55	2065
TCGA-55-5899-01A-11R-1628-07	Tumor	LUAD	R0	Discrepancy	M0	N1	T1a	Yes	Alive	MALE	WHITE	58	930
TCGA-55-6543-01A-11R-1755-07	Tumor	LUAD	Not Available	Stage IA	MX	N0	T1b	Yes	Alive	FEMALE	WHITE	60	435
TCGA-55-6642-01A-11R-1858-07	Tumor	LUAD	R0	Stage IB	MX	N0	T2	Yes	Alive	MALE	WHITE	63	2449
TCGA-55-6712-01A-11R-1858-07	Tumor	LUAD	R1	Stage IIA	MX	N1	T2a	Yes	Alive	MALE	WHITE	71	171

TCGA-55-6968-01A-11R-1949-07	Tumor	LUAD	Not Available	Stage IV	M1	N0	T1	Yes	Dead	MALE	WHITE	61	1293
TCGA-55-6968-11A-01R-1949-07	Normal	LUAD	Not Available	Stage IV	M1	N0	T1	Yes	Dead	MALE	WHITE	61	1293
TCGA-55-6969-11A-01R-1949-07	Normal	LUAD	Not Available	Stage IB	M0	N0	T2	Yes	Alive	MALE	WHITE	52	1239
TCGA-55-6970-01A-11R-1949-07	Tumor	LUAD	R1	Stage IIIA	MX	N2	T2	Yes	Dead	FEMALE	WHITE	67	464
TCGA-55-6970-11A-01R-1949-07	Normal	LUAD	R1	Stage IIIA	MX	N2	T2	Yes	Dead	FEMALE	WHITE	67	464
TCGA-55-6971-01A-11R-1949-07	Tumor	LUAD	R0	Stage IB	MX	N0	T2	Yes	Alive	FEMALE	WHITE	59	1400
TCGA-55-6971-11A-01R-1949-07	Normal	LUAD	R0	Stage IB	MX	N0	T2	Yes	Alive	FEMALE	WHITE	59	1400
TCGA-55-6972-01A-11R-1949-07	Tumor	LUAD	R0	Stage IB	M0	N0	T2	Yes	Alive	MALE	WHITE	72	1632

TCGA-55-6972-11A-01R-1949-07	Normal	LUAD	R0	Stage IB	M0	N0	T2	Yes	Alive	MALE	WHITE	72	1632
TCGA-55-6975-01A-11R-1949-07	Tumor	LUAD	Not Available	Stage IIB	M0	N1	T2	Yes	Dead	MALE	WHITE	61	118
TCGA-55-6975-11A-01R-1949-07	Normal	LUAD	Not Available	Stage IIB	M0	N1	T2	Yes	Dead	MALE	WHITE	61	118
TCGA-55-6978-01A-11R-1949-07	Tumor	LUAD	Not Available	Stage IIA	MX	N0	T2b	No	Dead	MALE	WHITE	81	176
TCGA-55-6978-11A-01R-1949-07	Normal	LUAD	Not Available	Stage IIA	MX	N0	T2b	No	Dead	MALE	WHITE	81	176
TCGA-55-6979-01A-11R-1949-07	Tumor	LUAD	Not Available	Stage IIB	M0	N1	T2	Yes	Dead	FEMALE	WHITE	59	237
TCGA-55-6979-11A-01R-1949-07	Normal	LUAD	Not Available	Stage IIB	M0	N1	T2	Yes	Dead	FEMALE	WHITE	59	237
TCGA-55-6980-01A-11R-1949-07	Tumor	LUAD	R0	Stage IA	M0	N0	T1	No	Alive	MALE	WHITE	56	2109

TCGA-55-6980-11A-01R-1949-07	Normal	LUAD	R0	Stage IA	M0	N0	T1	No	Alive	MALE	WHITE	56	2109
TCGA-55-6981-01A-11R-1949-07	Tumor	LUAD	R0	Stage IIIA	M0	N2	T1	Yes	Dead	FEMALE	WHITE	53	1379
TCGA-55-6981-11A-01R-1949-07	Normal	LUAD	R0	Stage IIIA	M0	N2	T1	Yes	Dead	FEMALE	WHITE	53	1379
TCGA-55-6982-01A-11R-1949-07	Tumor	LUAD	Not Available	Stage IIB	M0	N1	T2	No	Dead	FEMALE	WHITE	79	995
TCGA-55-6982-11A-01R-1949-07	Normal	LUAD	Not Available	Stage IIB	M0	N1	T2	No	Dead	FEMALE	WHITE	79	995
TCGA-55-6983-01A-11R-1949-07	Tumor	LUAD	R0	Stage IIB	M0	N1	T2	Yes	Alive	MALE	WHITE	81	2823
TCGA-55-6983-11A-01R-1949-07	Normal	LUAD	R0	Stage IIB	M0	N1	T2	Yes	Alive	MALE	WHITE	81	2823
TCGA-55-6984-01A-11R-1949-07	Tumor	LUAD	Not Available	Stage IIB	M0	N1	T2	Not Available	Dead	FEMALE	WHITE	71	760

TCGA-55-6984-11A-01R-1949-07	Normal	LUAD	Not Available	Stage IIB	M0	N1	T2	Not Available	Dead	FEMALE	WHITE	71	760
TCGA-55-6985-01A-11R-1949-07	Tumor	LUAD	R0	Stage IB	MX	N0	T2	Yes	Alive	FEMALE	WHITE	58	1233
TCGA-55-6985-11A-01R-1949-07	Normal	LUAD	R0	Stage IB	MX	N0	T2	Yes	Alive	FEMALE	WHITE	58	1233
TCGA-55-6986-01A-11R-1949-07	Tumor	LUAD	Not Available	Stage IB	M0	N0	T2	No	Alive	FEMALE	WHITE	74	3261
TCGA-55-6986-11A-01R-1949-07	Normal	LUAD	Not Available	Stage IB	M0	N0	T2	No	Alive	FEMALE	WHITE	74	3261
TCGA-55-6987-01A-11R-1949-07	Tumor	LUAD	Not Available	Stage IA	M0	N0	T1	Yes	Alive	MALE	WHITE	77	2137
TCGA-55-7227-01A-11R-2039-07	Tumor	LUAD	R0	Stage IIIA	MX	N1	T3	Yes	Alive	MALE	WHITE	77	952
TCGA-55-7281-01A-11R-2039-07	Tumor	LUAD	R0	Stage IA	M0	N0	T1b	Yes	Alive	FEMALE	WHITE	70	872

TCGA-55-7283-01A-11R-2039-07	Tumor	LUAD	Not Available	Stage IIIA	MX	N2	T3	Yes	Alive	FEMALE	WHITE	76	609
TCGA-55-7284-01B-11R-2241-07	Tumor	LUAD	R0	Stage IIB	MX	N0	T3	Yes	Alive	MALE	WHITE	74	243
TCGA-55-7570-01A-11R-2039-07	Tumor	LUAD	R0	Stage IA	MX	N0	T1a	Yes	Alive	MALE	BLACK OR AFRICAN AMERICAN	60	824
TCGA-55-7573-01A-11R-2039-07	Tumor	LUAD	Not Available	Stage IA	MX	N0	T1b	Yes	Alive	FEMALE	WHITE	72	487
TCGA-55-7574-01A-11R-2039-07	Tumor	LUAD	R1	Stage IB	M0	N0	T2a	Yes	Alive	FEMALE	WHITE	64	995
TCGA-55-7576-01A-11R-2066-07	Tumor	LUAD	R0	Stage IB	M0	N0	T2a	Yes	Alive	MALE	BLACK OR AFRICAN AMERICAN	54	670

TCGA-55-7724-01A-11R-2170-07	Tumor	LUAD	R0	Stage IB	MX	N0	T2a	Yes	Alive	FEMALE	WHITE	76	705
TCGA-55-7725-01A-11R-2170-07	Tumor	LUAD	R0	Stage IA	MX	N0	T1a	Yes	Alive	FEMALE	WHITE	68	442
TCGA-55-7726-01A-11R-2170-07	Tumor	LUAD	R0	Stage IA	MX	N0	T1b	Yes	Alive	FEMALE	WHITE	72	652
TCGA-55-7727-01A-11R-2170-07	Tumor	LUAD	R0	Stage IIIA	MX	N2	T1a	Yes	Alive	MALE	WHITE	70	119
TCGA-55-7728-01A-11R-2187-07	Tumor	LUAD	R0	Stage IB	MX	N0	T2a	Yes	Alive	FEMALE	WHITE	64	704
TCGA-55-7815-01A-11R-2170-07	Tumor	LUAD	R0	Stage IB	MX	N0	T2a	No	Alive	MALE	Not Available	76	773
TCGA-55-7816-01A-11R-2170-07	Tumor	LUAD	Not Available	Stage IV	MX	NX	TX	No	Alive	FEMALE	WHITE	49	468

TCGA-55-7903-01A-11R-2170-07	Tumor	LUAD	R0	Stage IA	MX	N0	T1b	Yes	Alive	MALE	WHITE	64	567
TCGA-55-7907-01A-11R-2170-07	Tumor	LUAD	R0	Stage IIA	MX	N1	T2a	Yes	Alive	MALE	WHITE	77	343
TCGA-55-7910-01A-11R-2170-07	Tumor	LUAD	R0	Stage IIA	M0	N0	T2b	Yes	Alive	FEMALE	BLACK OR AFRICAN AMERICAN	50	1040
TCGA-55-7911-01A-11R-2170-07	Tumor	LUAD	R0	Stage IA	MX	N0	T1a	Yes	Alive	FEMALE	WHITE	70	537
TCGA-55-7913-01B-11R-2241-07	Tumor	LUAD	R0	Stage IA	MX	N0	T1b	Yes	Alive	FEMALE	BLACK OR AFRICAN AMERICAN	61	561
TCGA-55-7914-01A-11R-2170-07	Tumor	LUAD	R0	Stage IIA	MX	N1	T1b	Yes	Alive	FEMALE	WHITE	71	187

TCGA-55-7994-01A-11R-2187-07	Tumor	LUAD	R0	Stage IIB	MX	N0	T3	Yes	Alive	MALE	WHITE	81	603
TCGA-55-7995-01A-11R-2187-07	Tumor	LUAD	R0	Stage IA	M0	N0	T1b	Yes	Alive	FEMALE	WHITE	73	889
TCGA-55-8085-01A-11R-2241-07	Tumor	LUAD	R0	Stage IA	M0	N0	T1b	Yes	Alive	MALE	WHITE	64	904
TCGA-55-8087-01A-11R-2241-07	Tumor	LUAD	R0	Stage IB	MX	N0	T2a	No	Alive	FEMALE	WHITE	59	462
TCGA-55-8089-01A-11R-2241-07	Tumor	LUAD	R0	Stage IA	M0	N0	T1a	Yes	Alive	MALE	WHITE	56	702
TCGA-55-8090-01A-11R-2241-07	Tumor	LUAD	R0	Stage IA	M0	N0	T1a	Yes	Alive	MALE	WHITE	80	598
TCGA-55-8091-01A-11R-2241-07	Tumor	LUAD	R0	Stage IB	MX	N0	T2	Yes	Alive	MALE	WHITE	74	600
TCGA-55-8092-01A-11R-2241-07	Tumor	LUAD	R0	Stage IIB	MX	N0	T3	Yes	Alive	MALE	WHITE	75	154

TCGA-55-8094-01A-11R-2241-07	Tumor	LUAD	R0	Stage IV	M1b	N0	T2b	Yes	Alive	MALE	WHITE	51	541
TCGA-55-8096-01A-11R-2241-07	Tumor	LUAD	Not Available	Stage IB	MX	N0	T2a	Yes	Alive	FEMALE	WHITE	67	719
TCGA-55-8097-01A-11R-2241-07	Tumor	LUAD	R0	Stage IA	MX	N0	T1a	Yes	Alive	FEMALE	WHITE	60	476
TCGA-55-8203-01A-11R-2241-07	Tumor	LUAD	R0	Stage IA	M0	N0	T1b	Yes	Alive	FEMALE	WHITE	69	547
TCGA-55-8204-01A-11R-2241-07	Tumor	LUAD	R0	Stage IB	MX	N0	T2a	Yes	Alive	FEMALE	WHITE	87	515
TCGA-55-8205-01A-11R-2241-07	Tumor	LUAD	R0	Stage IIA	M0	N0	T2b	Yes	Alive	FEMALE	WHITE	76	599
TCGA-55-8206-01A-11R-2241-07	Tumor	LUAD	R0	Stage IA	M0	N0	T1b	No	Alive	MALE	WHITE	56	888
TCGA-55-8207-01A-11R-2241-07	Tumor	LUAD	R0	Stage IB	MX	N0	T2a	Yes	Alive	MALE	WHITE	73	977

TCGA-55-8208-01A-11R-2241-07	Tumor	LUAD	R0	Stage IA	M0	N0	T1b	Yes	Alive	FEMALE	WHITE	73	674
TCGA-55-8299-01A-11R-2287-07	Tumor	LUAD	R0	Stage IA	MX	N0	T1b	Yes	Alive	FEMALE	WHITE	61	469
TCGA-55-8301-01A-11R-2287-07	Tumor	LUAD	R0	Stage IB	MX	N0	T2a	Yes	Alive	MALE	WHITE	58	534
TCGA-55-8302-01A-11R-2326-07	Tumor	LUAD	R0	Stage IB	MX	N0	T2	Yes	Alive	MALE	WHITE	54	478
TCGA-55-8505-01A-11R-2403-07	Tumor	LUAD	R0	Stage IIIA	MX	N2	T1a	No	Alive	MALE	WHITE	62	440
TCGA-55-8506-01A-11R-2403-07	Tumor	LUAD	R0	Stage IIB	MX	N0	T3	Yes	Alive	FEMALE	WHITE	62	11
TCGA-55-8507-01A-11R-2403-07	Tumor	LUAD	R0	Stage IA	MX	N0	T1a	Yes	Alive	MALE	WHITE	53	418
TCGA-55-8508-01A-11R-2403-07	Tumor	LUAD	R0	Stage IIA	MX	N1	T2a	Yes	Alive	FEMALE	BLACK OR AFRICAN AMERICAN	60	617

TCGA-55-8510-01A-11R-2403-07	Tumor	LUAD	R0	Stage IB	MX	N0	T2a	Yes	Alive	FEMALE	WHITE	55	539
TCGA-55-8511-01A-11R-2403-07	Tumor	LUAD	R0	Stage IB	MX	N0	T2a	Yes	Alive	FEMALE	WHITE	73	552
TCGA-55-8512-01A-11R-2403-07	Tumor	LUAD	R0	Stage IV	M1b	N1	T1a	Yes	Alive	MALE	WHITE	41	607
TCGA-55-8513-01A-11R-2403-07	Tumor	LUAD	R0	Stage IIB	MX	N0	T3	No	Alive	FEMALE	WHITE	77	791
TCGA-55-8514-01A-11R-2403-07	Tumor	LUAD	R0	Stage IB	MX	N0	T2a	Yes	Alive	FEMALE	BLACK OR AFRICAN AMERICAN	70	520
TCGA-55-8614-01A-11R-2403-07	Tumor	LUAD	R0	Stage IB	MX	N0	T2a	Yes	Alive	MALE	WHITE	76	536
TCGA-55-8615-01A-11R-2403-07	Tumor	LUAD	R0	Stage IIIA	MX	N2	T3	Yes	Alive	MALE	WHITE	67	446

TCGA-55-8616-01A-11R-2403-07	Tumor	LUAD	R0	Stage IB	M0	N0	T2a	Yes	Alive	FEMALE	WHITE	58	48
TCGA-55-8619-01A-11R-2403-07	Tumor	LUAD	R0	Stage IIB	MX	N0	T3	No	Alive	FEMALE	WHITE	72	416
TCGA-55-8620-01A-11R-2403-07	Tumor	LUAD	R0	Stage IV	M1b	N1	T1a	Yes	Alive	MALE	WHITE	60	375
TCGA-55-8621-01A-11R-2403-07	Tumor	LUAD	R0	Stage IA	MX	N0	T1a	Yes	Alive	FEMALE	WHITE	75	515
TCGA-55-A48X-01A-11R- A24H-07	Tumor	LUAD	R0	Stage IIA	M0	N1	T1b	Yes	Alive	FEMALE	WHITE	63	689
TCGA-55-A48Y-01A-11R- A24H-07	Tumor	LUAD	R0	Stage IIA	M0	N0	T2b	Yes	Alive	MALE	WHITE	69	630
TCGA-55-A48Z-01A-12R- A24X-07	Tumor	LUAD	R0	Stage IIIB	MX	N3	T1a	Yes	Alive	FEMALE	WHITE	60	651
TCGA-55-A490-01A-11R- A466-07	Tumor	LUAD	R0	Stage IIA	MX	N0	T2b	Yes	Alive	MALE	WHITE	78	99

TCGA-55-A491-01A-11R- A24H-07	Tumor	LUAD	R0	Stage IA	MX	N0	T1b	Yes	Alive	FEMALE	WHITE	81	626
TCGA-55-A492-01A-11R- A24H-07	Tumor	LUAD	R0	Stage IA	MX	N0	T1a	Yes	Alive	FEMALE	WHITE	70	596
TCGA-55-A493-01A-11R- A24H-07	Tumor	LUAD	R0	Stage IB	M0	N0	T2a	Yes	Alive	FEMALE	WHITE	54	28
TCGA-55-A494-01A-11R- A24X-07	Tumor	LUAD	R0	Stage IB	MX	N0	T2a	Yes	Alive	FEMALE	WHITE	61	481
TCGA-55-A4DF-01A-11R-A24H-07	Tumor	LUAD	R0	Stage IA	MX	N0	T1b	Yes	Alive	MALE	WHITE	88	614
TCGA-55-A4DG-01A-11R-A24H-07	Tumor	LUAD	R0	Stage IA	MX	N0	T1b	Yes	Alive	MALE	WHITE	71	608
TCGA-55-A57B-01A-12R-A39D-07	Tumor	LUAD	R0	Stage IA	M0	N0	T1b	No	Alive	FEMALE	BLACK OR AFRICAN AMERICAN	80	546

TCGA-62-8394-01A-11R-2326-07	Tumor	LUAD	R0	Stage IIIB	M0	N2	T4	No	Dead	FEMALE	WHITE	65	139
TCGA-62-8395-01A-11R-2326-07	Tumor	LUAD	R0	Stage IIB	M0	N0	T3	No	Alive	FEMALE	WHITE	80	1216
TCGA-62-8397-01A-11R-2326-07	Tumor	LUAD	R0	Stage IIB	M0	N0	T3	No	Alive	FEMALE	WHITE	70	1289
TCGA-62-8398-01A-11R-2326-07	Tumor	LUAD	R0	Stage IIIA	M0	N2	T2	Yes	Dead	MALE	WHITE	55	444
TCGA-62-8399-01A-21R-2326-07	Tumor	LUAD	R0	Stage IIIA	M0	N2	T2	Yes	Alive	MALE	WHITE	62	2696
TCGA-62-8402-01A-11R-2326-07	Tumor	LUAD	R0	Stage IIIA	M0	N2	T2	No	Alive	FEMALE	WHITE	73	1498
TCGA-62-A46O-01A-11R-A24H-07	Tumor	LUAD	R0	Stage IB	M0	N0	T2	Yes	Dead	FEMALE	WHITE	65	1454

TCGA-62-A46P-01A-11R- A24H-07	Tumor	LUAD	R0	Stage IB	M0	N0	T2	Yes	Dead	MALE	WHITE	65	594
TCGA-62-A46R-01A-11R-A24H-07	Tumor	LUAD	R0	Stage IB	M0	N0	T2	Yes	Dead	FEMALE	WHITE	54	1725
TCGA-62-A46S-01A-11R- A24H-07	Tumor	LUAD	R0	Stage IB	M0	N0	T2	Yes	Dead	MALE	WHITE	73	1653
TCGA-62-A46V-01A-11R- A24H-07	Tumor	LUAD	R0	Stage IB	M0	N0	T2	Yes	Alive	FEMALE	WHITE	78	2199
TCGA-62-A46Y-01A-11R- A24H-07	Tumor	LUAD	R0	Stage IIIA	M0	N2	T2	No	Dead	FEMALE	WHITE	70	414
TCGA-62-A470-01A-11R- A24H-07	Tumor	LUAD	R0	Stage IB	M0	N0	T2	Yes	Dead	MALE	WHITE	84	1194
TCGA-62-A471-01A-12R- A24H-07	Tumor	LUAD	R0	Stage IIB	M0	N1	T2b	Yes	Alive	MALE	WHITE	64	1246
TCGA-62-A472-01A-11R- A24H-07	Tumor	LUAD	R0	Stage IIB	M0	N0	T3	Yes	Alive	MALE	WHITE	70	910

TCGA-64-1676-01A-01R-0946-07	Tumor	LUAD	R0	Stage IA	M0	N0	T1a	Yes	Alive	MALE	WHITE	58	1728
TCGA-64-1677-01A-01R-0946-07	Tumor	LUAD	R0	Stage IIIA	M0	N2	T2	Yes	Dead	FEMALE	WHITE	77	628
TCGA-64-1678-01A-01R-0946-07	Tumor	LUAD	R0	Discrepancy	M0	N0	T2b	Yes	Alive	FEMALE	WHITE	70	1189
TCGA-64-1679-01A-21R-2066-07	Tumor	LUAD	R0	Stage IIIA	M0	N2	T1	Yes	Alive	FEMALE	WHITE	58	2488
TCGA-64-1680-01A-02R-0946-07	Tumor	LUAD	R0	Stage IV	M1	N2	T2a	Yes	Alive	MALE	WHITE	63	1126
TCGA-64-1681-01A-11R-2066-07	Tumor	LUAD	R0	Stage IA	M0	N0	T1	Yes	Dead	FEMALE	WHITE	61	1167
TCGA-64-5774-01A-01R-1628-07	Tumor	LUAD	R0	Stage IB	M0	N0	T2	Yes	Alive	MALE	WHITE	60	2676
TCGA-64-5775-01A-01R-1628-07	Tumor	LUAD	R0	Stage IIIA	M0	N0	T4	Yes	Dead	MALE	WHITE	71	62

TCGA-64-5778-01A-01R-1628-07	Tumor	LUAD	R0	Stage IB	M0	N0	T2	Yes	Alive	MALE	WHITE	60	1305
TCGA-64-5779-01A-01R-1628-07	Tumor	LUAD	R0	Stage IIIA	M0	N2	T2	Yes	Alive	MALE	WHITE	61	864
TCGA-64-5781-01A-01R-1628-07	Tumor	LUAD	R0	Stage IB	M0	N0	T2	Yes	Alive	FEMALE	WHITE	55	1559
TCGA-64-5815-01A-01R-1628-07	Tumor	LUAD	R0	Stage IIB	M0	N1	T2	Yes	Alive	MALE	WHITE	74	866
TCGA-67-3770-01A-01R-0946-07	Tumor	LUAD	R0	Stage IA	M0	N0	T1	Yes	Alive	FEMALE	WHITE	70	610
TCGA-67-3771-01A-01R-0946-07	Tumor	LUAD	R0	Stage IA	M0	N0	T1	Yes	Alive	FEMALE	BLACK OR AFRICAN AMERICAN	77	610
TCGA-67-3772-01A-01R-0946-07	Tumor	LUAD	Not Available	Stage IB	M0	N0	T2	No	Alive	FEMALE	WHITE	82	573

TCGA-67-3773-01A-01R-0946-07	Tumor	LUAD	R0	Stage IB	M0	N0	T2	Yes	Alive	FEMALE	WHITE	84	427
TCGA-67-3774-01A-01R-0946-07	Tumor	LUAD	RX	Stage IB	M0	N0	T2	Yes	Alive	FEMALE	WHITE	73	385
TCGA-67-4679-01B-01R-1755-07	Tumor	LUAD	R0	Discrepancy	M0	N0	T3	Yes	Alive	MALE	WHITE	69	448
TCGA-67-6215-01A-11R-1755-07	Tumor	LUAD	R0	Stage IB	M0	N0	T2a	No	Alive	FEMALE	WHITE	52	174
TCGA-67-6216-01A-11R-1755-07	Tumor	LUAD	R0	Stage IA	M0	N0	T1a	No	Alive	FEMALE	WHITE	57	141
TCGA-67-6217-01A-11R-1755-07	Tumor	LUAD	R0	Stage IIA	M0	N1	T2a	Yes	Alive	FEMALE	WHITE	73	422
TCGA-69-7760-01A-11R-2170-07	Tumor	LUAD	Not Available	Stage IIB	M0	N0	T3	No	Alive	MALE	WHITE	73	202
TCGA-69-7761-01A-11R-2170-07	Tumor	LUAD	Not Available	Stage IB	MX	N0	T2a	Yes	Alive	MALE	WHITE	84	186

TCGA-69-7763-01A-11R-2170-07	Tumor	LUAD	Not Available	Stage IA	M0	N0	T1b	Yes	Alive	MALE	WHITE	69	690
TCGA-69-7764-01A-11R-2170-07	Tumor	LUAD	Not Available	Stage IA	M0	N0	T1b	Yes	Alive	MALE	WHITE	75	414
TCGA-69-7765-01A-11R-2170-07	Tumor	LUAD	Not Available	Discrepancy	MX	N0	T4	Yes	Alive	MALE	BLACK OR AFRICAN AMERICAN	56	165
TCGA-69-7973-01A-11R-2187-07	Tumor	LUAD	Not Available	Stage IB	M0	N0	T2a	Yes	Alive	FEMALE	WHITE	42	230
TCGA-69-7974-01A-11R-2187-07	Tumor	LUAD	Not Available	Stage IIIA	MX	N2	T2a	Yes	Alive	FEMALE	WHITE	54	184
TCGA-69-7978-01A-11R-2187-07	Tumor	LUAD	Not Available	Stage IIB	MX	N1	T2b	Yes	Alive	MALE	WHITE	59	134
TCGA-69-7979-01A-11R-2187-07	Tumor	LUAD	Not Available	Stage IB	MX	N0	T2a	Yes	Alive	FEMALE	WHITE	71	408

TCGA-69-7980-01A-11R-2187-07	Tumor	LUAD	Not Available	Stage I	M0	N0	T1b	Yes	Alive	FEMALE	WHITE	70	411
TCGA-69-8253-01A-11R-2287-07	Tumor	LUAD	Not Available	Stage IIA	MX	N1	T1a	Yes	Alive	FEMALE	BLACK OR AFRICAN AMERICAN	59	426
TCGA-69-8254-01A-11R-2287-07	Tumor	LUAD	Not Available	Not Available	Not Available	Not Available	T2b	Yes	Alive	MALE	WHITE	85	409
TCGA-69-8255-01A-11R-2287-07	Tumor	LUAD	Not Available	Stage IA	M0	N0	T1a	Yes	Alive	MALE	WHITE	71	129
TCGA-69-8453-01A-12R-2326-07	Tumor	LUAD	Not Available	Stage IIB	MX	N0	T3	Yes	Alive	MALE	WHITE	77	813
TCGA-69-A59K-01A-11R- A262-07	Tumor	LUAD	Not Evaluated	Stage IIB	M0	N0	T3	Yes	Alive	FEMALE	BLACK OR AFRICAN AMERICAN	60	591

TCGA-71-6725-01A-11R-1858-07	Tumor	LUAD	RX	Stage IB	M0	N0	T2	Yes	Alive	FEMALE	ASIAN	48	256
TCGA-71-8520-01A-11R-2403-07	Tumor	LUAD	R0	Stage IB	M0	N0	T2	No	Alive	FEMALE	ASIAN	60	210
TCGA-73-4658-01A-01R-1755-07	Tumor	LUAD	R0	Stage IB	M0	N0	T2	Yes	Dead	FEMALE	WHITE	80	1600
TCGA-73-4659-01A-01R-1206-07	Tumor	LUAD	R0	Stage IIIA	M0	N2	T2	Yes	Dead	MALE	WHITE	66	711
TCGA-73-4662-01A-01R-1206-07	Tumor	LUAD	R0	Stage IA	M0	N0	T1	Yes	Alive	FEMALE	WHITE	65	2515
TCGA-73-4666-01A-01R-1206-07	Tumor	LUAD	R0	Stage IV	M1	N0	T1	Yes	Alive	FEMALE	WHITE	52	800

TCGA-73-4668-01A-01R-1206-07	Tumor	LUAD	R0	Stage IIB	M0	N1	T2	Yes	Alive	FEMALE	AMERICAN INDIAN OR ALASKA NATIVE	66	467
TCGA-73-4670-01A-01R-1206-07	Tumor	LUAD	R0	Stage IV	M1	N0	T2	Yes	Alive	FEMALE	WHITE	69	131
TCGA-73-4675-01A-01R-1206-07	Tumor	LUAD	RX	Stage IIIA	M0	N1	T3	Yes	Alive	MALE	WHITE	59	922
TCGA-73-4676-01A-01R-1755-07	Tumor	LUAD	R0	Stage IIA	M0	N1	T2a	Yes	Alive	MALE	WHITE	45	281
TCGA-73-4676-11A-01R-1755-07	Normal	LUAD	R0	Stage IIA	M0	N1	T2a	Yes	Alive	MALE	WHITE	45	281
TCGA-73-4677-01A-01R-1206-07	Tumor	LUAD	R0	Discrepancy	M0	N0	T2a	Yes	Dead	MALE	WHITE	74	38

TCGA-73-7498-01A-12R-2187-07	Tumor	LUAD	R0	Stage IA	M0	N0	T1b	Yes	Alive	FEMALE	WHITE	58	1189
TCGA-73-7499-01A-11R-2187-07	Tumor	LUAD	R0	Stage IB	M0	N0	T2a	No	Alive	FEMALE	WHITE	81	1531
TCGA-73-A9RS-01A-11R-A41B-07	Tumor	LUAD	R0	Stage IIB	M0	N0	T3	Yes	Alive	MALE	BLACK OR AFRICAN AMERICAN	41	340
TCGA-75-5122-01A-01R-1755-07	Tumor	LUAD	R0	Stage IB	M0	N0	T2	Yes	Dead	MALE	Not Available	Not Available	
TCGA-75-5125-01A-01R-1755-07	Tumor	LUAD	R0	Stage IIB	M0	N1	T2	Yes	Alive	MALE	Not Available	Not Available	2027
TCGA-75-5126-01A-01R-1755-07	Tumor	LUAD	R2	Stage IIIA	M0	N2	T3	Yes	Alive	FEMALE	Not Available	Not Available	
TCGA-75-5146-01A-01R-1628-07	Tumor	LUAD	R0	Stage IB	M0	N0	T2	Yes	Alive	MALE	Not Available	Not Available	2368

TCGA-75-5147-01A-01R-1628-07	Tumor	LUAD	R0	Stage IB	M0	N0	T2	No	Alive	FEMALE	Not Available	Not Available	1333
TCGA-75-6203-01A-11R-1755-07	Tumor	LUAD	R0	Stage IIIA	M0	N2	T2	No	Alive	FEMALE	Not Available	Not Available	
TCGA-75-6205-01A-11R-1755-07	Tumor	LUAD	R0	Stage IB	M0	N0	T2a	No	Dead	FEMALE	Not Available	Not Available	
TCGA-75-6206-01A-11R-1755-07	Tumor	LUAD	R0	Stage IB	M0	N0	T2	Yes	Alive	MALE	Not Available	Not Available	2590
TCGA-75-6207-01A-11R-1755-07	Tumor	LUAD	R0	Stage IIIA	M0	N2	T2	Yes	Dead	MALE	Not Available	Not Available	
TCGA-75-6211-01A-11R-1755-07	Tumor	LUAD	R0	Stage IB	M0	N0	T2	Yes	Dead	FEMALE	Not Available	Not Available	
TCGA-75-6212-01A-11R-1755-07	Tumor	LUAD	R0	Stage IIB	M0	N1	T2	No	Alive	FEMALE	Not Available	Not Available	1516
TCGA-75-6214-01A-41R-1949-07	Tumor	LUAD	R0	Stage IIIA	M0	N2	T2	Yes	Alive	FEMALE	Not Available	Not Available	1115

TCGA-75-7025-01A-12R-1949-07	Tumor	LUAD	R0	Stage IB	M0	N0	T2	Yes	Alive	MALE	Not Available	Not Available	3305
TCGA-75-7027-01A-11R-1949-07	Tumor	LUAD	R0	Stage IB	M0	N0	T2	Yes	Alive	MALE	Not Available	Not Available	3059
TCGA-75-7030-01A-11R-1949-07	Tumor	LUAD	R0	Stage IIB	M0	N0	T3	No	Alive	MALE	Not Available	Not Available	
TCGA-75-7031-01A-11R-1949-07	Tumor	LUAD	R0	Stage IB	M0	N0	T2	Yes	Alive	FEMALE	Not Available	Not Available	
TCGA-78-7143-01A-11R-2039-07	Tumor	LUAD	R0	Stage IB	M0	N0	T2	No	Dead	FEMALE	WHITE	62	4961
TCGA-78-7145-01A-11R-2039-07	Tumor	LUAD	R1	Stage IV	M1	N1	T4	Yes	Dead	FEMALE	WHITE	52	826
TCGA-78-7146-01A-11R-2039-07	Tumor	LUAD	R0	Stage IIIA	M0	N2	T2	Yes	Dead	FEMALE	WHITE	71	173
TCGA-78-7147-01A-11R-2039-07	Tumor	LUAD	R0	Stage IIB	M0	N1	T2	Yes	Dead	FEMALE	WHITE	67	586

TCGA-78-7148-01A-11R-2039-07	Tumor	LUAD	R0	Stage IIB	M0	N1	T2	Yes	Dead	MALE	WHITE	71	626
TCGA-78-7149-01A-11R-2039-07	Tumor	LUAD	R0	Stage IIIB	M0	N0	T4	Yes	Alive	MALE	WHITE	71	3940
TCGA-78-7150-01A-21R-2039-07	Tumor	LUAD	R0	Stage IIB	M0	N1	T2	Yes	Dead	MALE	WHITE	59	666
TCGA-78-7152-01A-11R-2039-07	Tumor	LUAD	R0	Stage IB	M0	N0	T2	Yes	Alive	MALE	WHITE	65	1215
TCGA-78-7153-01A-11R-2039-07	Tumor	LUAD	R0	Stage IB	M0	N0	T2	Yes	Alive	FEMALE	WHITE	65	3635
TCGA-78-7154-01A-11R-2039-07	Tumor	LUAD	R0	Stage IIIA	M0	N2	T3	Yes	Dead	MALE	WHITE	72	593
TCGA-78-7155-01A-11R-2039-07	Tumor	LUAD	R0	Stage IB	M0	N0	T2	Yes	Dead	MALE	WHITE	68	1171
TCGA-78-7156-01A-11R-2039-07	Tumor	LUAD	R0	Stage IV	M1	N1	T4	Yes	Dead	MALE	WHITE	62	976

TCGA-78-7158-01A-11R-2039-07	Tumor	LUAD	R1	Stage IIIB	M0	N2	T4	Yes	Dead	FEMALE	WHITE	59	179
TCGA-78-7159-01A-11R-2039-07	Tumor	LUAD	R0	Stage IA	M0	NX	T1	Yes	Alive	FEMALE	WHITE	60	1974
TCGA-78-7160-01A-11R-2039-07	Tumor	LUAD	R0	Stage IV	M1	N2	T4	Yes	Alive	MALE	WHITE	61	697
TCGA-78-7161-01A-11R-2039-07	Tumor	LUAD	R1	Stage IIB	M0	N0	T3	Yes	Alive	FEMALE	WHITE	69	291
TCGA-78-7162-01A-21R-2066-07	Tumor	LUAD	R0	Stage IA	M0	N0	T1	Yes	Dead	MALE	WHITE	75	3169
TCGA-78-7163-01A-12R-2066-07	Tumor	LUAD	R0	Stage IB	M0	N0	T2	Yes	Alive	MALE	WHITE	60	7248
TCGA-78-7166-01A-12R-2066-07	Tumor	LUAD	R0	Stage IIB	M0	N1	T2	Yes	Dead	MALE	WHITE	84	258
TCGA-78-7167-01A-11R-2066-07	Tumor	LUAD	R0	Stage IV	M1	N0	T2	Yes	Dead	MALE	WHITE	77	2681

TCGA-78-7220-01A-11R-2039-07	Tumor	LUAD	R0	Stage IIIA	M0	N2	T2	Yes	Dead	FEMALE	WHITE	53	807
TCGA-78-7535-01A-11R-2066-07	Tumor	LUAD	R0	Stage IB	M0	N0	T2	Yes	Dead	MALE	WHITE	45	949
TCGA-78-7536-01A-11R-2066-07	Tumor	LUAD	R0	Stage IIIA	M0	N2	T2	Yes	Dead	MALE	WHITE	69	244
TCGA-78-7537-01A-11R-2066-07	Tumor	LUAD	R0	Stage IB	M0	N0	T2	Yes	Dead	MALE	Not Available	72	1622
TCGA-78-7539-01A-11R-2066-07	Tumor	LUAD	R0	Stage IIA	M0	N0	T2b	Yes	Alive	FEMALE	WHITE	75	791
TCGA-78-7540-01A-11R-2066-07	Tumor	LUAD	R0	Stage IB	M0	N0	T2	No	Alive	FEMALE	WHITE	66	1197
TCGA-78-7542-01A-21R-2066-07	Tumor	LUAD	R0	Stage IB	M0	N0	T2	Yes	Dead	MALE	WHITE	56	321
TCGA-78-7633-01A-11R-2066-07	Tumor	LUAD	R0	Stage IB	M0	N0	T2	Yes	Alive	MALE	WHITE	67	1528

TCGA-78-8640-01A-11R-2403-07	Tumor	LUAD	R0	Stage IIA	M0	N1	T1	Yes	Alive	MALE	Not Evaluated	59	7062
TCGA-78-8648-01A-11R-2403-07	Tumor	LUAD	R0	Stage IIB	M0	N0	T3	Yes	Dead	FEMALE	Not Evaluated	58	1209
TCGA-78-8655-01A-11R-2403-07	Tumor	LUAD	R0	Stage IA	M0	N0	T1	Yes	Alive	FEMALE	Not Evaluated	77	2360
TCGA-78-8660-01A-11R-2403-07	Tumor	LUAD	R0	Stage IIB	M0	N1	T2	Yes	Dead	MALE	Not Evaluated	69	321
TCGA-78-8662-01A-11R-2403-07	Tumor	LUAD	R0	Stage IB	M0	N0	T2	Yes	Dead	FEMALE	Not Evaluated	53	3361
TCGA-80-5607-01A-31R-1949-07	Tumor	LUAD	R0	Stage IIB	M0	N1	T2	Yes	Alive	FEMALE	Not Available	Not Available	
TCGA-80-5608-01A-31R-1949-07	Tumor	LUAD	R0	Stage IA	M0	N0	T1	Yes	Alive	FEMALE	Not Available	Not Available	2832
TCGA-80-5611-01A-01R-1628-07	Tumor	LUAD	R0	Stage IB	M0	N0	T2	Yes	Alive	MALE	Not Available	Not Available	2595

TCGA-83-5908-01A-21R-2287-07	Tumor	LUAD	R0	Stage IA	M0	N0	T1	Yes	Alive	FEMALE	WHITE	59	824
TCGA-86-6562-01A-11R-1755-07	Tumor	LUAD	R0	Stage IIA	M0	N1	T2a	No	Alive	MALE	WHITE	52	376
TCGA-86-6851-01A-11R-1949-07	Tumor	LUAD	Not Available	Stage IIA	M0	N1	T1b	Yes	Alive	FEMALE	WHITE	73	179
TCGA-86-7701-01A-11R-2170-07	Tumor	LUAD	R0	Stage IV	M1	N0	T2	No	Alive	MALE	WHITE	66	947
TCGA-86-7711-01A-11R-2066-07	Tumor	LUAD	R0	Stage IIA	M0	N1	T2a	Yes	Alive	MALE	WHITE	70	1046
TCGA-86-7713-01A-11R-2066-07	Tumor	LUAD	R0	Stage IIA	M0	N0	T2b	No	Alive	MALE	WHITE	70	1157
TCGA-86-7714-01A-12R-2170-07	Tumor	LUAD	R0	Stage IIIA	M0	N2	T1b	Yes	Alive	FEMALE	BLACK OR AFRICAN AMERICAN	61	625

TCGA-86-7953-01A-11R-2187-07	Tumor	LUAD	RX	Stage IA	M0	N0	T1b	No	Alive	FEMALE	WHITE	69	997
TCGA-86-7954-01A-11R-2187-07	Tumor	LUAD	R0	Stage IB	M0	N0	T2	Yes	Alive	FEMALE	WHITE	68	605
TCGA-86-7955-01A-11R-2187-07	Tumor	LUAD	RX	Stage IB	M0	N0	T2a	No	Alive	MALE	WHITE	62	1072
TCGA-86-8054-01A-11R-2241-07	Tumor	LUAD	R0	Stage IIB	M0	N1	T2b	Yes	Alive	MALE	WHITE	61	1148
TCGA-86-8055-01A-11R-2241-07	Tumor	LUAD	R0	Stage IIA	M0	N1	T2a	Yes	Alive	MALE	WHITE	79	124
TCGA-86-8056-01A-11R-2241-07	Tumor	LUAD	R0	Stage IIIA	M0	N0	T4	Yes	Alive	FEMALE	WHITE	63	139
TCGA-86-8073-01A-11R-2241-07	Tumor	LUAD	R0	Stage IB	M0	N0	T2a	Yes	Alive	MALE	WHITE	58	740

TCGA-86-8074-01A-11R-2241-07	Tumor	LUAD	R0	Stage IIA	M0	N1	T1b	Yes	Alive	FEMALE	WHITE	62	24
TCGA-86-8075-01A-11R-2241-07	Tumor	LUAD	R0	Stage IB	M0	N0	T2	No	Alive	FEMALE	WHITE	66	694
TCGA-86-8076-01A-31R-2241-07	Tumor	LUAD	R0	Stage IA	M0	N0	T1	No	Alive	MALE	WHITE	42	993
TCGA-86-8278-01A-11R-2287-07	Tumor	LUAD	R0	Stage IIB	M0	N1	T2	No	Alive	FEMALE	WHITE	63	944
TCGA-86-8279-01A-11R-2287-07	Tumor	LUAD	R0	Stage IIA	M0	N1	T2a	No	Alive	MALE	WHITE	46	949
TCGA-86-8280-01A-11R-2287-07	Tumor	LUAD	R0	Stage IIA	M0	N0	T2b	No	Alive	FEMALE	WHITE	54	701
TCGA-86-8281-01A-11R-2287-07	Tumor	LUAD	Not Evaluated	Stage IA	M0	NX	T1	Yes	Alive	MALE	WHITE	75	0
TCGA-86-8358-01A-11R-2326-07	Tumor	LUAD	R0	Stage IB	M0	N0	T2a	Yes	Alive	MALE	WHITE	44	653

TCGA-86-8359-01A-11R-2326-07	Tumor	LUAD	R0	Stage IIIA	M0	N2	T3	Yes	Alive	MALE	WHITE	52	444
TCGA-86-8585-01A-11R-2403-07	Tumor	LUAD	R0	Stage IB	M0	N0	T2a	No	Alive	MALE	WHITE	57	353
TCGA-86-8668-01A-11R-2403-07	Tumor	LUAD	R0	Stage IA	M0	N0	T1b	No	Alive	FEMALE	WHITE	61	423
TCGA-86-8669-01A-11R-2403-07	Tumor	LUAD	R0	Stage IA	M0	N0	T1b	Yes	Alive	MALE	WHITE	64	938
TCGA-86-8671-01A-11R-2403-07	Tumor	LUAD	R0	Stage IIB	M0	N1	T2b	No	Alive	FEMALE	WHITE	72	839
TCGA-86-8672-01A-21R-2403-07	Tumor	LUAD	R0	Stage IIB	M0	N0	T3	No	Alive	MALE	WHITE	59	19
TCGA-86-8673-01A-11R-2403-07	Tumor	LUAD	R0	Stage IB	M0	N0	T2	Yes	Alive	MALE	WHITE	61	862
TCGA-86-8674-01A-21R-2403-07	Tumor	LUAD	R0	Stage IIA	M0	N1	T2a	Yes	Alive	MALE	WHITE	50	806

TCGA-86-A456-01A-11R- A24H-07	Tumor	LUAD	R0	Stage IA	M0	N0	T1a	Yes	Alive	FEMALE	ASIAN	78	896
TCGA-86-A4D0-01A-11R-A24H-07	Tumor	LUAD	R0	Stage IIA	M0	N0	T2b	Yes	Alive	MALE	WHITE	48	116
TCGA-86-A4JF-01A-11R- A24X-07	Tumor	LUAD	R0	Stage IIB	M0	N0	T3	Yes	Alive	MALE	WHITE	56	737
TCGA-86-A4P7-01A-11R- A24X-07	Tumor	LUAD	R0	Stage IB	M0	N0	T2a	No	Alive	FEMALE	WHITE	63	415
TCGA-86-A4P8-01A-11R- A24X-07	Tumor	LUAD	Not Evaluated	Stage IIIA	MX	N2	T1b	No	Alive	FEMALE	WHITE	59	805
TCGA-91-6828-01A-11R-1858-07	Tumor	LUAD	R0	Stage IA	M0	N0	T1a	Yes	Alive	MALE	WHITE	70	323
TCGA-91-6828-11A-01R-1858-07	Normal	LUAD	R0	Stage IA	M0	N0	T1a	Yes	Alive	MALE	WHITE	70	323
TCGA-91-6829-01A-21R-1858-07	Tumor	LUAD	R0	Stage IB	MX	N0	T2	Yes	Dead	MALE	WHITE	78	1258

TCGA-91-6829-11A-01R-1858-07	Normal	LUAD	R0	Stage IB	MX	N0	T2	Yes	Dead	MALE	WHITE	78	1258
TCGA-91-6830-01A-11R-1949-07	Tumor	LUAD	Not Available	Stage IIA	MX	N1	T1	Yes	Alive	FEMALE	WHITE	65	60
TCGA-91-6831-01A-11R-1858-07	Tumor	LUAD	Not Available	Stage IB	MX	N0	T2	Yes	Alive	MALE	WHITE	66	310
TCGA-91-6831-11A-02R-1858-07	Normal	LUAD	Not Available	Stage IB	MX	N0	T2	Yes	Alive	MALE	WHITE	66	310
TCGA-91-6835-01A-11R-1858-07	Tumor	LUAD	R0	Stage IA	M0	N0	T1	Yes	Alive	FEMALE	WHITE	81	79
TCGA-91-6835-11A-01R-1858-07	Normal	LUAD	R0	Stage IA	M0	N0	T1	Yes	Alive	FEMALE	WHITE	81	79
TCGA-91-6836-01A-21R-1858-07	Tumor	LUAD	Not Available	Stage IB	MX	N0	T2	Yes	Alive	FEMALE	WHITE	52	417
TCGA-91-6836-11A-01R-1858-07	Normal	LUAD	Not Available	Stage IB	MX	N0	T2	Yes	Alive	FEMALE	WHITE	52	417

TCGA-91-6840-01A-11R-1949-07	Tumor	LUAD	R0	Stage IA	M0	N0	T1b	Yes	Alive	FEMALE	WHITE	59	372
TCGA-91-6847-01A-11R-1949-07	Tumor	LUAD	Not Available	Stage IB	MX	N0	T2	Yes	Alive	FEMALE	WHITE	62	842
TCGA-91-6847-11A-01R-1949-07	Normal	LUAD	Not Available	Stage IB	MX	N0	T2	Yes	Alive	FEMALE	WHITE	62	842
TCGA-91-6848-01A-11R-1949-07	Tumor	LUAD	Not Available	Stage IIIA	MX	N2	T2	Yes	Alive	MALE	WHITE	59	224
TCGA-91-6849-01A-11R-1949-07	Tumor	LUAD	Not Available	Stage IIIA	MX	N2	T2	Yes	Alive	FEMALE	BLACK OR AFRICAN AMERICAN	75	35
TCGA-91-6849-11A-01R-1949-07	Normal	LUAD	Not Available	Stage IIIA	MX	N2	T2	Yes	Alive	FEMALE	BLACK OR AFRICAN AMERICAN	75	35
TCGA-91-7771-01A-11R-2170-07	Tumor	LUAD	Not Available	Stage IIB	MX	N0	T3	Yes	Alive	MALE	WHITE	62	492

TCGA-91-8496-01A-11R-2403-07	Tumor	LUAD	Not Evaluated	Stage IB	MX	NX	T2a	No	Alive	FEMALE	WHITE	63	505
TCGA-91-8497-01A-11R-2403-07	Tumor	LUAD	Not Evaluated	Stage IA	MX	N0	T1a	No	Alive	FEMALE	WHITE	75	434
TCGA-91-8499-01A-11R-2403-07	Tumor	LUAD	Not Evaluated	Stage IA	MX	N0	T1b	Yes	Alive	FEMALE	WHITE	76	36
TCGA-91-A4BC-01A-11R-A24H-07	Tumor	LUAD	Not Evaluated	Stage IIA	MX	N0	T2b	Yes	Alive	MALE	WHITE	59	44
TCGA-91-A4BD-01A-11R-A24H-07	Tumor	LUAD	RX	Stage IIA	MX	N1	T1b	Yes	Alive	MALE	WHITE	78	603
TCGA-93-7347-01A-11R-2187-07	Tumor	LUAD	R0	Stage IA	MX	N0	T1a	Yes	Alive	FEMALE	WHITE	76	683
TCGA-93-7348-01A-21R-2039-07	Tumor	LUAD	R0	Stage IA	MX	N0	T1a	Yes	Alive	FEMALE	WHITE	75	531
TCGA-93-8067-01A-11R-2287-07	Tumor	LUAD	RX	Stage IB	MX	N0	T2a	Yes	Alive	MALE	ASIAN	77	186

TCGA-93-A4JN-01A-11R- A24X-07	Tumor	LUAD	RX	Stage IV	M1a	N0	T2a	Yes	Alive	MALE	WHITE	71	718
TCGA-93-A4JO-01A-21R-A24X-07	Tumor	LUAD	RX	Stage IA	MX	N0	T1a	Yes	Dead	MALE	WHITE	70	33
TCGA-93-A4JP-01A-11R- A24X-07	Tumor	LUAD	RX	Stage IV	M1b	NX	TX	No	Alive	MALE	ASIAN	64	578
TCGA-93-A4JQ-01A-11R-A24X-07	Tumor	LUAD	RX	Stage IA	MX	N0	T1b	Yes	Alive	MALE	WHITE	49	526
TCGA-95-7039-01A-11R-1949-07	Tumor	LUAD	Not Available	Stage IIB	MX	N0	T3	Yes	Alive	FEMALE	WHITE	54	1272
TCGA-95-7043-01A-11R-1949-07	Tumor	LUAD	Not Available	Stage IA	MX	N0	T1a	Yes	Alive	FEMALE	WHITE	63	503
TCGA-95-7562-01A-11R-2241-07	Tumor	LUAD	RX	Stage IIA	M0	N1	T2a	Yes	Dead	MALE	BLACK OR AFRICAN AMERICAN	71	87

TCGA-95-7567-01A-11R-2066-07	Tumor	LUAD	Not Available	Stage IIB	M0	N1	T2b	Yes	Alive	MALE	WHITE	61	568
TCGA-95-7944-01A-11R-2187-07	Tumor	LUAD	R0	Stage IA	M0	N0	T1a	Yes	Alive	MALE	WHITE	71	377
TCGA-95-7947-01A-11R-2187-07	Tumor	LUAD	R0	Stage IA	M0	N0	T1a	Yes	Alive	MALE	WHITE	67	477
TCGA-95-7948-01A-11R-2187-07	Tumor	LUAD	R0	Stage IB	M0	N0	T2a	Yes	Alive	FEMALE	WHITE	42	476
TCGA-95-8039-01A-11R-2241-07	Tumor	LUAD	RX	Stage IA	MX	N0	T1	No	Alive	MALE	WHITE	72	830
TCGA-95-8494-01A-11R-2326-07	Tumor	LUAD	Not Evaluated	Stage IIA	M0	N1	T2a	Yes	Alive	MALE	WHITE	67	84
TCGA-95-A4VK-01A-11R-A262-07	Tumor	LUAD	RX	Stage IIIA	M0	N2	T2b	Yes	Alive	FEMALE	WHITE	74	651

TCGA-95-A4VN-01A-11R-A262-07	Tumor	LUAD	R0	Stage IIA	M0	N1	T2a	Yes	Alive	FEMALE	WHITE	62	553
TCGA-95-A4VP-01A-21R-A262-07	Tumor	LUAD	Not Evaluated	Stage IIIA	M0	N2	T2b	Yes	Alive	FEMALE	WHITE	66	605
TCGA-97-7546-01A-11R-2039-07	Tumor	LUAD	R0	Stage IA	MX	N0	T1	Yes	Alive	FEMALE	WHITE	76	1285
TCGA-97-7547-01A-11R-2039-07	Tumor	LUAD	Not Available	Stage IB	MX	N0	T2	Yes	Alive	FEMALE	WHITE	67	1965
TCGA-97-7552-01A-11R-2039-07	Tumor	LUAD	Not Available	Stage IB	MX	N0	T2	Yes	Alive	MALE	WHITE	70	1932
TCGA-97-7553-01A-21R-2039-07	Tumor	LUAD	Not Available	Stage IA	MX	N0	T1	Yes	Alive	FEMALE	WHITE	58	1870
TCGA-97-7554-01A-11R-2039-07	Tumor	LUAD	R0	Stage IIIA	M0	N2	T2a	Yes	Alive	FEMALE	WHITE	83	775
TCGA-97-7937-01A-11R-2170-07	Tumor	LUAD	Not Available	Stage IB	MX	N0	T2a	Yes	Alive	MALE	WHITE	65	564

TCGA-97-7938-01A-11R-2170-07	Tumor	LUAD	R0	Stage IA	MX	N0	T1a	Yes	Dead	FEMALE	WHITE	76	18
TCGA-97-7941-01A-11R-2187-07	Tumor	LUAD	R0	Stage IA	MX	N0	T1b	Yes	Alive	FEMALE	WHITE	72	484
TCGA-97-8171-01A-11R-2287-07	Tumor	LUAD	Not Available	Stage IV	M1a	N2	T2a	Yes	Alive	MALE	ASIAN	81	568
TCGA-97-8172-01A-11R-2287-07	Tumor	LUAD	Not Available	Stage IB	M0	N0	T2a	Yes	Alive	FEMALE	WHITE	75	545
TCGA-97-8174-01A-11R-2287-07	Tumor	LUAD	Not Available	Stage IIA	M0	N0	T2b	Yes	Dead	MALE	WHITE	67	164
TCGA-97-8175-01A-11R-2287-07	Tumor	LUAD	Not Available	Stage IB	M0	N0	T2a	Yes	Alive	FEMALE	WHITE	55	551
TCGA-97-8176-01A-11R-2403-07	Tumor	LUAD	R0	Stage IIIA	M0	N1	T3	Yes	Alive	MALE	WHITE	63	468
TCGA-97-8177-01A-11R-2287-07	Tumor	LUAD	Not Available	Stage IB	M0	N0	T2a	No	Alive	FEMALE	WHITE	59	499

TCGA-97-8179-01A-11R-2287-07	Tumor	LUAD	Not Available	Stage IA	M0	N0	T1a	Yes	Alive	MALE	WHITE	72	435
TCGA-97-8547-01A-11R-2403-07	Tumor	LUAD	Not Evaluated	Stage IIIA	MX	N2	T2a	No	Alive	FEMALE	Not Evaluated	78	657
TCGA-97-8552-01A-11R-2403-07	Tumor	LUAD	Not Evaluated	Stage I	MX	N0	T1a	No	Alive	FEMALE	WHITE	55	626
TCGA-97-A4LX-01A-11R- A24X-07	Tumor	LUAD	Not Evaluated	Stage IB	M0	N0	T2a	Yes	Alive	MALE	WHITE	81	614
TCGA-97-A4M0-01A-11R-A24X-07	Tumor	LUAD	Not Evaluated	Stage IB	M0	N0	T2a	Yes	Alive	FEMALE	WHITE	60	652
TCGA-97-A4M1-01A-11R-A24X-07	Tumor	LUAD	Not Evaluated	Stage IA	M0	N0	T1a	Yes	Alive	FEMALE	WHITE	52	601
TCGA-97-A4M2-01A-12R-A24X-07	Tumor	LUAD	R0	Stage IA	M0	N0	T1a	Yes	Alive	MALE	WHITE	66	624
TCGA-97-A4M3-01A-11R-A24X-07	Tumor	LUAD	Not Evaluated	Stage IA	M0	N0	T1b	Yes	Alive	FEMALE	WHITE	69	540

TCGA-97-A4M5-01A-11R-A24X-07	Tumor	LUAD	Not Evaluated	Stage IA	M0	N0	T1b	Yes	Alive	MALE	WHITE	83	634
TCGA-97-A4M6-01A-11R-A24X-07	Tumor	LUAD	Not Evaluated	Stage IA	M0	N0	T1a	No	Alive	FEMALE	WHITE	45	568
TCGA-97-A4M7-01A-11R-A24X-07	Tumor	LUAD	R0	Stage IA	M0	N0	T1b	Yes	Alive	MALE	WHITE	74	629
TCGA-99-7458-01A-11R-2039-07	Tumor	LUAD	R0	Stage IIIA	M0	N0	T4	Yes	Alive	FEMALE	WHITE	74	747
TCGA-99-8025-01A-11R-2241-07	Tumor	LUAD	R0	Stage IIIA	M0	N2	T3	Yes	Alive	FEMALE	BLACK OR AFRICAN AMERICAN	72	1060
TCGA-99-8028-01A-11R-2241-07	Tumor	LUAD	R0	Stage IA	M0	N0	T1a	Yes	Alive	FEMALE	BLACK OR AFRICAN AMERICAN	50	1118
TCGA-99-8032-01A-11R-2241-07	Tumor	LUAD	R0	Stage IA	M0	N0	T1a	Yes	Alive	MALE	WHITE	61	44

TCGA-99-8033-01A-11R-2241-07	Tumor	LUAD	R0	Stage IV	M1	NX	TX	No	Alive	FEMALE	WHITE	74	656
TCGA-99-AA5R-01A-11R-A39D-07	Tumor	LUAD	R0	Stage IA	M0	N0	T1a	Yes	Alive	FEMALE	BLACK OR AFRICAN AMERICAN	70	658
TCGA-J2-8192-01A-11R-2241-07	Tumor	LUAD	Not Available	Stage IIA	MX	N1	T2a	No	Alive	FEMALE	WHITE	65	739
TCGA-J2-8194-01A-11R-2241-07	Tumor	LUAD	Not Available	Stage IIB	MX	N0	T3	Yes	Alive	FEMALE	WHITE	69	724
TCGA-J2-A4AD-01A-11R-A24H-07	Tumor	LUAD	RX	Stage IA	MX	N0	T1a	Yes	Alive	FEMALE	WHITE	61	550
TCGA-J2-A4AE-01A-21R-A24H-07	Tumor	LUAD	R0	Stage IA	MX	N0	T1a	No	Alive	FEMALE	WHITE	77	1079
TCGA-J2-A4AG-01A-11R-A24H-07	Tumor	LUAD	R0	Stage IA	MX	N0	T1b	Yes	Alive	FEMALE	WHITE	66	988

TCGA-L4-A4E5-01A-11R- A24X-07	Tumor	LUAD	R0	Stage I	M0	N0	T1	Yes	Alive	FEMALE	WHITE	48	578
TCGA-L4-A4E6-01A-11R- A24H-07	Tumor	LUAD	R0	Stage IA	M0	N0	T1	Yes	Alive	MALE	WHITE	67	435
TCGA-L9-A443-01A-12R- A24H-07	Tumor	LUAD	R0	Stage IA	MX	N0	T1a	Yes	Alive	FEMALE	WHITE	63	193
TCGA-L9-A444-01A-21R- A24H-07	Tumor	LUAD	R0	Stage IA	MX	N0	T1a	Yes	Alive	FEMALE	WHITE	60	307
TCGA-L9-A50W-01A-12R-A39D-07	Tumor	LUAD	R0	Stage IIA	MX	N1	T1b	Yes	Alive	MALE	BLACK OR AFRICAN AMERICAN	75	442
TCGA-L9-A5IP-01A-21R- A39D-07	Tumor	LUAD	R1	Stage IV	M1b	N2	T3	Yes	Dead	FEMALE	BLACK OR AFRICAN AMERICAN	40	58

TCGA-L9-A743-01A-43R- A39D-07	Tumor	LUAD	R0	Stage IIA	M0	N1	T2a	Yes	Alive	MALE	BLACK OR AFRICAN AMERICAN	56	664
TCGA-L9-A7SV-01A-11R-A39D-07	Tumor	LUAD	R0	Stage IIA	M0	N1	T2a	Yes	Alive	MALE	BLACK OR AFRICAN AMERICAN	69	565
TCGA-L9-A8F4-01A-11R- A39D-07	Tumor	LUAD	R0	Stage IB	MX	N0	T2a	Yes	Alive	FEMALE	BLACK OR AFRICAN AMERICAN	64	476
TCGA-MN-A4N1-01A-11R-A24X-07	Tumor	LUAD	R0	Stage IIA	M0	N1	T2a	Yes	Alive	MALE	BLACK OR AFRICAN AMERICAN	60	827
TCGA-MN-A4N4-01A-12R-A24X-07	Tumor	LUAD	R0	Stage IA	M0	N0	T1b	Yes	Alive	MALE	WHITE	57	1175

TCGA-MN-A4N5-01A-11R-A24X-07	Tumor	LUAD	R0	Stage IA	M0	N0	T1a	Yes	Alive	MALE	WHITE	63	84
TCGA-MP-A4SV-01A-11R-A24X-07	Tumor	LUAD	R0	Stage IB	M0	N0	T2	Yes	Dead	MALE	Unknown	67	2620
TCGA-MP-A4SW-01A-21R-A24X-07	Tumor	LUAD	R0	Stage IIB	M0	N1	T2	Yes	Dead	MALE	WHITE	53	1778
TCGA-MP-A4SY-01A-21R-A24X-07	Tumor	LUAD	R0	Stage IIB	M0	N1	T2	Yes	Dead	MALE	WHITE	61	1501
TCGA-MP-A4T4-01A-11R- A262-07	Tumor	LUAD	R0	Stage IIB	M0	N1	T2	Yes	Dead	FEMALE	WHITE	68	2617
TCGA-MP-A4T6-01A-32R- A262-07	Tumor	LUAD	R0	Stage IIIA	MX	N2	T1	Yes	Dead	FEMALE	WHITE	76	1790
TCGA-MP-A4T7-01A-11R- A24X-07	Tumor	LUAD	R0	Stage IV	M1	N0	T2	Yes	Dead	FEMALE	Unknown	75	167
TCGA-MP-A4T8-01A-11R- A24X-07	Tumor	LUAD	R0	Stage IIIA	M0	N2	T2	Yes	Dead	MALE	Unknown	68	161

TCGA-MP-A4T9-01A-11R- A24X-07	Tumor	LUAD	RX	Stage IIIA	MX	N2	T2	Yes	Dead	FEMALE	WHITE	54	1265
TCGA-MP-A4TA-01A-21R-A24X-07	Tumor	LUAD	R0	Stage IA	M0	N0	T1	Yes	Dead	FEMALE	WHITE	75	950
TCGA-MP-A4TC-01A-11R-A24X-07	Tumor	LUAD	R0	Stage IIIA	M0	N2	T1	Yes	Dead	MALE	WHITE	77	74
TCGA-MP-A4TD-01A-32R-A262-07	Tumor	LUAD	RX	Stage IIIA	M0	N2	T2	Yes	Dead	MALE	WHITE	71	307
TCGA-MP-A4TE-01A-22R-A466-07	Tumor	LUAD	RX	Stage IIA	MX	N0	T2b	Yes	Dead	MALE	WHITE	56	896
TCGA-MP-A4TF-01A-11R- A262-07	Tumor	LUAD	R0	Stage IIA	M0	N0	T2b	Yes	Dead	FEMALE	WHITE	58	336
TCGA-MP-A4TH-01A-31R-A262-07	Tumor	LUAD	R0	Stage IA	M0	N0	T1a	Yes	Alive	FEMALE	WHITE	70	741

TCGA-MP-A4TI-01A-21R- A24X-07	Tumor	LUAD	R0	Stage IIA	M0	N1	T2a	Yes	Dead	MALE	WHITE	72	429
TCGA-MP-A4TJ-01A-51R- A262-07	Tumor	LUAD	R0	Stage IA	M0	N0	T1	Yes	Dead	FEMALE	Unknown	62	339
TCGA-MP-A4TK-01A-11R-A24X-07	Tumor	LUAD	R0	Stage IIB	MX	N1	T2	Yes	Dead	FEMALE	Unknown	56	582
TCGA-MP-A5C7-01A-11R-A262-07	Tumor	LUAD	R0	Stage IB	M0	N0	T2	Yes	Alive	FEMALE	WHITE	76	2248
TCGA-NJ-A4YF-01A-12R-A262-07	Tumor	LUAD	Not Evaluated	Stage IA	M0	N0	T1	Yes	Alive	FEMALE	BLACK OR AFRICAN AMERICAN	50	2161
TCGA-NJ-A4YG-01A-22R-A262-07	Tumor	LUAD	Not Evaluated	Stage IB	M0	N0	T2	Yes	Alive	MALE	WHITE	65	2261
TCGA-NJ-A4YI-01A-11R- A262-07	Tumor	LUAD	Not Evaluated	Stage IIIA	M0	N2	T2	Yes	Dead	FEMALE	WHITE	87	4

TCGA-NJ-A4YP-01A-11R-A262-07	Tumor	LUAD	Not Evaluated	Stage IB	M0	N0	T2a	Yes	Alive	MALE	WHITE	52	50
TCGA-NJ-A4YQ-01A-11R-A262-07	Tumor	LUAD	RX	Stage IA	M0	N0	T1b	Yes	Alive	FEMALE	WHITE	69	1432
TCGA-NJ-A55A-01A-11R- A262-07	Tumor	LUAD	Not Available	Stage IB	M0	N0	T2	Yes	Alive	FEMALE	WHITE	76	15
TCGA-NJ-A55O-01A-11R-A262-07	Tumor	LUAD	Not Available	Stage IIA	M0	N1	T1b	Yes	Alive	FEMALE	WHITE	56	13
TCGA-NJ-A55R-01A-11R-A262-07	Tumor	LUAD	Not Available	Stage IA	MX	N0	T1b	Yes	Alive	MALE	WHITE	67	603
TCGA-NJ-A7XG-01A-12R-A39D-07	Tumor	LUAD	Not Available	Stage IIIA	M0	N1	T4	Yes	Alive	MALE	BLACK OR AFRICAN AMERICAN	49	617
TCGA-O1-A52J-01A-11R- A262-07	Tumor	LUAD	Not Evaluated	Stage IA	MX	N0	T1	Yes	Dead	FEMALE	WHITE	74	1798

TCGA-S2-AA1A-01A-12R-A39D-07	Tumor	LUAD	R0	Stage IA	M0	N0	T1b	Yes	Alive	FEMALE	BLACK OR AFRICAN AMERICAN	68	513
TCGA-18-3406-01A-01R-0980-07	Tumor	LUSC	R0	Stage IA	M0	N0	T1	Yes	Dead	MALE	WHITE	67	371
TCGA-18-3407-01A-01R-0980-07	Tumor	LUSC	R0	Stage IB	M0	N0	T2	Yes	Dead	MALE	ASIAN	72	136
TCGA-18-3408-01A-01R-0980-07	Tumor	LUSC	R0	Stage IB	M0	N0	T2	Yes	Alive	FEMALE	WHITE	77	2304
TCGA-18-3409-01A-01R-0980-07	Tumor	LUSC	R0	Stage IA	M0	N0	T1	Yes	Alive	MALE	WHITE	74	3747
TCGA-18-3410-01A-01R-0980-07	Tumor	LUSC	R0	Stage IIB	M0	N0	T3	Yes	Dead	MALE	Not Available	81	146
TCGA-18-3411-01A-01R-0980-07	Tumor	LUSC	R0	Stage IIIA	M0	N2	T2	Yes	Alive	FEMALE	Not Available	63	3576
TCGA-18-3412-01A-01R-0980-07	Tumor	LUSC	R0	Stage IB	M0	N0	T2	Yes	Dead	MALE	WHITE	52	345

TCGA-18-3414-01A-01R-0980-07	Tumor	LUSC	R0	Stage IV	M1	N1	T4	Yes	Dead	MALE	ASIAN	73	716
TCGA-18-3415-01A-01R-0980-07	Tumor	LUSC	R0	Stage IB	M0	N0	T2	Yes	Alive	MALE	Not Available	77	2803
TCGA-18-3416-01A-01R-0980-07	Tumor	LUSC	R0	Stage IIB	M0	N1	T2	Yes	Alive	MALE	Not Available	83	973
TCGA-18-3417-01A-01R-1443-07	Tumor	LUSC	R0	Stage IV	M1	N1	T2	Yes	Alive	MALE	Not Available	65	1097
TCGA-18-3419-01A-01R-0980-07	Tumor	LUSC	R0	Stage IIB	M0	N1	T2	Yes	Alive	MALE	Not Available	73	2811
TCGA-18-3421-01A-01R-0980-07	Tumor	LUSC	R0	Stage IB	M0	N0	T2	Yes	Alive	MALE	WHITE	65	2645
TCGA-18-4083-01A-01R-1100-07	Tumor	LUSC	R0	Stage IIB	M0	N1	T2	Yes	Dead	MALE	WHITE	63	188
TCGA-18-4086-01A-01R-1100-07	Tumor	LUSC	R0	Stage IB	M0	N0	T2	Yes	Dead	MALE	Not Available	64	85

TCGA-18-4721-01A-01R-1443-07	Tumor	LUSC	R0	Stage IA	M0	N0	T1	Yes	Alive	MALE	WHITE	74	4694
TCGA-18-5592-01A-01R-1635-07	Tumor	LUSC	R0	Stage IIB	M0	N0	T3	Yes	Alive	MALE	Not Available	57	1519
TCGA-18-5595-01A-01R-1635-07	Tumor	LUSC	R0	Stage IB	M0	N0	T2	No	Dead	MALE	Not Available	50	827
TCGA-21-1070-01A-01R-0692-07	Tumor	LUSC	R0	Stage IIIA	M0	N0	T3	Yes	Alive	FEMALE	BLACK OR AFRICAN AMERICAN	60	3636
TCGA-21-1071-01A-01R-0692-07	Tumor	LUSC	R0	Stage IB	M0	N0	T2	Yes	Dead	MALE	WHITE	67	1426
TCGA-21-1072-01A-01R-0692-07	Tumor	LUSC	R0	Stage IB	M0	N0	T2	Yes	Alive	MALE	WHITE	75	3016
TCGA-21-1075-01A-01R-0692-07	Tumor	LUSC	R0	Stage IIB	M0	N1	T2	Yes	Alive	MALE	WHITE	57	2134

TCGA-21-1076-01A-01R-0692-07	Tumor	LUSC	R0	Stage IB	M0	N0	T2	Yes	Alive	FEMALE	WHITE	54	1852
TCGA-21-1076-01A-02R-0692-07	Tumor	LUSC	R0	Stage IB	M0	N0	T2	Yes	Alive	FEMALE	WHITE	54	1852
TCGA-21-1077-01A-01R-0692-07	Tumor	LUSC	R0	Stage IIB	M0	N1	T2	Yes	Dead	MALE	WHITE	64	1058
TCGA-21-1078-01A-01R-0692-07	Tumor	LUSC	R0	Stage IB	M0	N0	T2	Yes	Dead	MALE	WHITE	77	474
TCGA-21-1079-01A-01R-0692-07	Tumor	LUSC	R0	Stage IIIA	M0	N0	T3	Yes	Dead	MALE	WHITE	71	965
TCGA-21-1080-01A-01R-0692-07	Tumor	LUSC	R0	Stage IB	M0	N0	T2	Yes	Alive	MALE	WHITE	66	3724
TCGA-21-1081-01A-01R-0692-07	Tumor	LUSC	R0	Stage IIB	M0	N1	T2	Yes	Dead	MALE	WHITE	69	284
TCGA-21-1082-01A-01R-0692-07	Tumor	LUSC	R0	Stage IB	M0	N0	T2	Not Available	Alive	MALE	WHITE	61	3644

TCGA-21-1083-01A-01R-0692-07	Tumor	LUSC	R0	Stage IA	M0	N0	T1	Yes	Dead	MALE	WHITE	75	1315
TCGA-21-5782-01A-01R-1635-07	Tumor	LUSC	R0	Stage IB	M0	N0	T2	Yes	Dead	FEMALE	WHITE	68	962
TCGA-21-5783-01A-41R-2187-07	Tumor	LUSC	R0	Stage IB	M0	N0	T2	Yes	Dead	MALE	WHITE	76	2680
TCGA-21-5784-01A-01R-1635-07	Tumor	LUSC	R0	Stage IB	M0	N0	T2	Yes	Alive	FEMALE	WHITE	80	1268
TCGA-21-5786-01A-01R-1635-07	Tumor	LUSC	R0	Stage IB	M0	N0	T2	Yes	Alive	MALE	WHITE	64	1032
TCGA-21-5787-01A-01R-1635-07	Tumor	LUSC	R0	Stage IIIA	M0	N2	T2	Yes	Dead	MALE	BLACK OR AFRICAN AMERICAN	65	329
TCGA-21-A5DI-01A-31R- A26W-07	Tumor	LUSC	R0	Stage IA	M0	N0	T1b	Yes	Alive	MALE	WHITE	77	979

TCGA-22-0940-01A-01R-0692-07	Tumor	LUSC	R0	Stage IIA	M0	N1	T1	Yes	Dead	MALE	WHITE	71	669
TCGA-22-0944-01A-01R-0692-07	Tumor	LUSC	R0	Stage IB	M0	N0	T2	Yes	Dead	MALE	WHITE	61	223
TCGA-22-1000-01A-01R- A32Z-07	Tumor	LUSC	R0	Stage IB	M0	N0	T2	Yes	Dead	MALE	WHITE	76	454
TCGA-22-1002-01A-01R-0692-07	Tumor	LUSC	R0	Stage IA	M0	N0	T1	Yes	Dead	MALE	WHITE	69	131
TCGA-22-1005-01A-01R-0692-07	Tumor	LUSC	R0	Stage IA	M0	N0	T1	Yes	Dead	MALE	WHITE	63	1953
TCGA-22-1011-01A-01R-0692-07	Tumor	LUSC	R0	Stage IB	M0	N0	T2	Yes	Dead	MALE	WHITE	73	53
TCGA-22-1012-01A-01R-0692-07	Tumor	LUSC	R0	Stage IB	M0	N0	T2	Yes	Dead	FEMALE	WHITE	80	429
TCGA-22-1016-01A-01R-0692-07	Tumor	LUSC	R0	Stage IB	M0	N0	T2	Yes	Dead	MALE	WHITE	65	822

TCGA-22-1017-01A-01R-0692-07	Tumor	LUSC	R0	Stage IA	M0	N0	T1	Yes	Dead	MALE	WHITE	62	1485
TCGA-22-4591-01A-01R-1201-07	Tumor	LUSC	R0	Stage IIIA	M0	N2	T3	Yes	Dead	MALE	WHITE	80	623
TCGA-22-4593-01A-21R-1820-07	Tumor	LUSC	R0	Stage IIA	M0	N0	T2b	Yes	Dead	MALE	WHITE	77	1067
TCGA-22-4593-11A-01R-1820-07	Normal	LUSC	R0	Stage IIA	M0	N0	T2b	Yes	Dead	MALE	WHITE	77	1067
TCGA-22-4594-01A-01R-1201-07	Tumor	LUSC	R0	Stage IIIA	M0	N2	T3	Yes	Dead	FEMALE	Not Available	60	1470
TCGA-22-4595-01A-01R-1201-07	Tumor	LUSC	R0	Stage IIIA	Not Available	N2	T3	Yes	Dead	MALE	WHITE	57	734
TCGA-22-4596-01A-01R-1201-07	Tumor	LUSC	R0	Stage IB	M0	N0	T1b	Yes	Dead	FEMALE	WHITE	69	17
TCGA-22-4599-01A-01R-1443-07	Tumor	LUSC	R0	Stage IB	M0	N0	T2a	Yes	Dead	FEMALE	WHITE	73	1161

TCGA-22-4601-01A-01R-1443-07	Tumor	LUSC	R0	Stage IIIA	M0	N0	T4	Yes	Dead	FEMALE	WHITE	73	1057
TCGA-22-4604-01A-01R-1201-07	Tumor	LUSC	R0	Stage IIA	M0	N1	T2a	Yes	Dead	MALE	WHITE	73	399
TCGA-22-4605-01A-21R-2125-07	Tumor	LUSC	R0	Stage IB	M0	N0	T2	Yes	Dead	FEMALE	Not Available	78	974
TCGA-22-4607-01A-01R-1201-07	Tumor	LUSC	R0	Stage IB	M0	N0	T2a	Yes	Dead	MALE	WHITE	75	587
TCGA-22-4609-01A-21R-2125-07	Tumor	LUSC	R0	Stage IA	M0	N0	T1	Yes	Dead	MALE	WHITE	81	291
TCGA-22-4609-11A-01R-2125-07	Normal	LUSC	R0	Stage IA	M0	N0	T1	Yes	Dead	MALE	WHITE	81	291
TCGA-22-4613-01A-01R-1443-07	Tumor	LUSC	R0	Stage IA	M0	N0	T1b	Yes	Dead	FEMALE	WHITE	73	358
TCGA-22-5471-01A-01R-1635-07	Tumor	LUSC	R2	Stage IB	M0	N0	T2	Yes	Alive	MALE	WHITE	75	1845

TCGA-22-5471-11A-01R-1635-07	Normal	LUSC	R2	Stage IB	M0	N0	T2	Yes	Alive	MALE	WHITE	75	1845
TCGA-22-5472-01A-01R-1635-07	Tumor	LUSC	R0	Stage IB	M0	N0	T2a	Yes	Dead	MALE	WHITE	67	1975
TCGA-22-5472-11A-11R-1635-07	Normal	LUSC	R0	Stage IB	M0	N0	T2a	Yes	Dead	MALE	WHITE	67	1975
TCGA-22-5473-01A-01R-1635-07	Tumor	LUSC	R0	Discrepancy	M0	N0	T3	Yes	Dead	MALE	WHITE	78	1933
TCGA-22-5474-01A-01R-1635-07	Tumor	LUSC	R0	Stage IB	M0	N0	T2a	Yes	Dead	MALE	WHITE	74	445
TCGA-22-5477-01A-01R-1635-07	Tumor	LUSC	R0	Stage IA	M0	N0	T1	Yes	Dead	MALE	WHITE	65	1346
TCGA-22-5478-01A-01R-1635-07	Tumor	LUSC	R0	Stage IB	M0	N0	T2a	Yes	Dead	MALE	Not Available	79	24
TCGA-22-5478-11A-11R-1635-07	Normal	LUSC	R0	Stage IB	M0	N0	T2a	Yes	Dead	MALE	Not Available	79	24

TCGA-22-5479-01A-31R-1949-07	Tumor	LUSC	R0	Stage IB	M0	N0	T2	Yes	Alive	MALE	WHITE	64	2625
TCGA-22-5480-01A-01R-1635-07	Tumor	LUSC	R0	Stage IA	M0	N0	T1b	Yes	Dead	FEMALE	WHITE	66	2170
TCGA-22-5481-01A-31R-1949-07	Tumor	LUSC	R0	Stage IIB	M0	N1	T2	Yes	Dead	FEMALE	WHITE	72	2409
TCGA-22-5481-11A-01R-1949-07	Normal	LUSC	R0	Stage IIB	M0	N1	T2	Yes	Dead	FEMALE	WHITE	72	2409
TCGA-22-5482-01A-01R-1635-07	Tumor	LUSC	R0	Stage IB	M0	N0	T2a	Yes	Dead	MALE	WHITE	81	357
TCGA-22-5482-11A-01R-1635-07	Normal	LUSC	R0	Stage IB	M0	N0	T2a	Yes	Dead	MALE	WHITE	81	357
TCGA-22-5483-01A-01R-1820-07	Tumor	LUSC	R0	Stage IIA	M0	N1	T1a	Yes	Dead	MALE	WHITE	74	573
TCGA-22-5483-11A-11R-1820-07	Normal	LUSC	R0	Stage IIA	M0	N1	T1a	Yes	Dead	MALE	WHITE	74	573

TCGA-22-5485-01A-01R-1635-07	Tumor	LUSC	R0	Stage IA	M0	N0	T1a	Yes	Dead	FEMALE	WHITE	58	916
TCGA-22-5489-01A-01R-1635-07	Tumor	LUSC	R0	Stage IA	M0	N0	T1b	Yes	Dead	MALE	WHITE	64	1912
TCGA-22-5489-11A-01R-1635-07	Normal	LUSC	R0	Stage IA	M0	N0	T1b	Yes	Dead	MALE	WHITE	64	1912
TCGA-22-5491-01A-01R-1635-07	Tumor	LUSC	R0	Stage IA	M0	N0	T1a	Yes	Dead	MALE	WHITE	74	1713
TCGA-22-5491-11A-01R-1858-07	Normal	LUSC	R0	Stage IA	M0	N0	T1a	Yes	Dead	MALE	WHITE	74	1713
TCGA-22-5492-01A-01R-1635-07	Tumor	LUSC	R0	Stage IIIA	M0	N2	T2a	Yes	Dead	FEMALE	Not Available	73	506
TCGA-22-A5C4-01A-12R-A27Q-07	Tumor	LUSC	R0	Stage IIA	M0	N0	T2b	Yes	Alive	MALE	WHITE	70	671
TCGA-33-4532-01A-01R-1201-07	Tumor	LUSC	R0	Stage IB	M0	N0	T2	Yes	Alive	MALE	WHITE	68	3924

TCGA-33-4533-01A-01R-1201-07	Tumor	LUSC	R0	Stage IB	M0	N0	T2	Yes	Alive	FEMALE	BLACK OR AFRICAN AMERICAN	76	4068
TCGA-33-4538-01A-01R-1201-07	Tumor	LUSC	RX	Stage IIIA	M0	N2	T2	Yes	Dead	MALE	WHITE	66	2979
TCGA-33-4547-01A-01R-1201-07	Tumor	LUSC	R0	Stage IB	M0	N0	T2	Yes	Alive	MALE	WHITE	68	2419
TCGA-33-4566-01A-01R-1443-07	Tumor	LUSC	R0	Stage IB	M0	N0	T2	Yes	Dead	MALE	WHITE	40	5287
TCGA-33-4582-01A-01R-1443-07	Tumor	LUSC	R0	Stage IA	M0	N0	T1	Yes	Dead	MALE	WHITE	55	3149
TCGA-33-4583-01A-01R-1443-07	Tumor	LUSC	R0	Stage IA	M0	N0	T1	Yes	Alive	MALE	WHITE	73	4601
TCGA-33-4586-01A-01R-1443-07	Tumor	LUSC	Not Available	Stage IIIA	M0	N2	T2	Yes	Dead	MALE	WHITE	57	428

TCGA-33-4587-01A-11R-2125-07	Tumor	LUSC	R0	Stage IB	MX	N0	T2	Yes	Dead	FEMALE	WHITE	63	1656
TCGA-33-4587-11A-01R-2125-07	Normal	LUSC	R0	Stage IB	MX	N0	T2	Yes	Dead	FEMALE	WHITE	63	1656
TCGA-33-4589-01A-01R-1443-07	Tumor	LUSC	Not Available	Stage IIB	M0	N1	T2	Yes	Dead	FEMALE	WHITE	62	47
TCGA-33-6737-01A-11R-1820-07	Tumor	LUSC	Not Available	Stage IIIA	M0	N2	T2	Yes	Dead	MALE	WHITE	71	601
TCGA-33-6737-11A-01R-1820-07	Normal	LUSC	Not Available	Stage IIIA	M0	N2	T2	Yes	Dead	MALE	WHITE	71	601
TCGA-33-6738-01A-11R-1949-07	Tumor	LUSC	Not Available	Stage IIIA	MX	N2	T1	Yes	Alive	MALE	WHITE	80	1927
TCGA-33-A4WN-01A-11R-A262-07	Tumor	LUSC	R0	Stage IB	MX	N0	T2a	Yes	Dead	MALE	WHITE	60	143
TCGA-33-A5GW-01A-11R-A27Q-07	Tumor	LUSC	R0	Stage IIA	MX	N1	T1a	Yes	Alive	MALE	WHITE	67	9

TCGA-33-AAS8-01A-11R-A405-07	Tumor	LUSC	R0	Stage IA	MX	N0	T1	Yes	Dead	FEMALE	BLACK OR AFRICAN AMERICAN	59	1114
TCGA-33-AASB-01A-11R-A405-07	Tumor	LUSC	R0	Stage IB	MX	N0	T2	Yes	Dead	MALE	BLACK OR AFRICAN AMERICAN	66	211
TCGA-33-AASD-01A-11R-A405-07	Tumor	LUSC	R0	Stage IA	MX	N0	T1	Yes	Dead	MALE	BLACK OR AFRICAN AMERICAN	83	3376
TCGA-33-AASI-01A-22R- A405-07	Tumor	LUSC	RX	Stage IIB	MX	N1	T2	Yes	Dead	FEMALE	BLACK OR AFRICAN AMERICAN	65	1344
TCGA-33-AASJ-01A-11R-A405-07	Tumor	LUSC	R0	Stage IB	MX	N0	T2	Yes	Dead	MALE	BLACK OR AFRICAN AMERICAN	60	3600

TCGA-33-AASL-01A-11R-A405-07	Tumor	LUSC	R0	Stage IA	MX	N0	T1	Yes	Dead	FEMALE	BLACK OR AFRICAN AMERICAN	57	826
TCGA-34-2596-01A-01R-0851-07	Tumor	LUSC	R0	Stage IIB	M0	N1	T2	Yes	Dead	MALE	WHITE	70	80
TCGA-34-2600-01A-01R-0851-07	Tumor	LUSC	R0	Stage IA	M0	N0	T1	Yes	Dead	FEMALE	WHITE	76	1874
TCGA-34-2608-01A-02R-0851-07	Tumor	LUSC	R0	Stage IB	M0	N0	T2	Yes	Dead	MALE	WHITE	84	1000
TCGA-34-5231-01A-21R-1820-07	Tumor	LUSC	Not Available	Stage IA	M0	N0	T1	Yes	Alive	MALE	WHITE	72	1984
TCGA-34-5232-01A-21R-1820-07	Tumor	LUSC	Not Available	Stage IIA	M0	N1	T1	Not Available	Alive	FEMALE	BLACK OR AFRICAN AMERICAN	75	2471

TCGA-34-5234-01A-01R-1635-07	Tumor	LUSC	R0	Stage IA	M0	N0	T1	Yes	Alive	FEMALE	WHITE	71	2271
TCGA-34-5236-01A-21R-1820-07	Tumor	LUSC	Not Available	Stage IIB	M0	N0	T3	Not Available	Dead	MALE	WHITE	60	276
TCGA-34-5239-01A-21R-1820-07	Tumor	LUSC	Not Available	Stage IIIA	M0	N0	T4	Not Available	Alive	MALE	WHITE	75	1834
TCGA-34-5240-01A-01R-1443-07	Tumor	LUSC	Not Available	Stage IIB	M0	N1	T2	Yes	Alive	FEMALE	WHITE	73	1541
TCGA-34-5241-01A-01R-1443-07	Tumor	LUSC	Not Available	Stage IB	M0	N0	T2	Yes	Alive	MALE	WHITE	79	515
TCGA-34-5927-01A-11R-1820-07	Tumor	LUSC	Not Available	Stage IA	M0	N0	T1	Not Available	Alive	FEMALE	WHITE	70	1361
TCGA-34-5928-01A-11R-1820-07	Tumor	LUSC	Not Available	Stage IIB	M0	N1	T2	Yes	Alive	FEMALE	WHITE	83	1196

TCGA-34-5929-01A-11R-1820-07	Tumor	LUSC	Not Available	Stage IB	M0	N0	T2	Yes	Dead	FEMALE	WHITE	78	151
TCGA-34-7107-01A-11R-1949-07	Tumor	LUSC	Not Available	Stage II	M0	N0	T2a	Yes	Dead	MALE	WHITE	70	34
TCGA-34-7107-11A-01R-1949-07	Normal	LUSC	Not Available	Stage II	M0	N0	T2a	Yes	Dead	MALE	WHITE	70	34
TCGA-34-8454-01A-11R-2326-07	Tumor	LUSC	R0	Stage IIIA	M0	N1	T3	Yes	Alive	FEMALE	WHITE	62	1180
TCGA-34-8454-11A-01R-2326-07	Normal	LUSC	R0	Stage IIIA	M0	N1	T3	Yes	Alive	FEMALE	WHITE	62	1180
TCGA-34-8455-01A-11R-2326-07	Tumor	LUSC	R0	Stage IV	M1a	N0	T4	Yes	Dead	MALE	WHITE	67	123
TCGA-34-8456-01A-21R-2326-07	Tumor	LUSC	R0	Stage IIA	M0	N1	T2a	Yes	Alive	FEMALE	WHITE	71	804
TCGA-34-A5IX-01A-12R- A27Q-07	Tumor	LUSC	Not Available	Stage IIB	M0	N0	T3	Yes	Alive	MALE	WHITE	80	1031

TCGA-37-3783-01A-01R-1201-07	Tumor	LUSC	R0	Stage IIIA	M0	N2	T3	Not Available	Alive	MALE	WHITE	51	122
TCGA-37-3789-01A-01R-0980-07	Tumor	LUSC	R0	Stage IB	Not Available	N0	T2	No	Alive	MALE	WHITE	65	13
TCGA-37-3792-01A-01R-0980-07	Tumor	LUSC	R0	Stage IB	M0	N0	T2	No	Alive	MALE	WHITE	69	12
TCGA-37-4129-01A-01R-1100-07	Tumor	LUSC	R0	Stage IA	M0	N0	T1b	Yes	Alive	FEMALE	WHITE	52	242
TCGA-37-4130-01A-01R-1100-07	Tumor	LUSC	R0	Stage IA	M0	N0	T1b	Yes	Alive	MALE	WHITE	56	247
TCGA-37-4132-01A-01R-1100-07	Tumor	LUSC	R0	Stage IV	M1	N0	T2	No	Alive	FEMALE	WHITE	61	227
TCGA-37-4133-01A-01R-1100-07	Tumor	LUSC	R0	Stage IIIA	M0	N0	T4	Yes	Alive	MALE	WHITE	63	238
TCGA-37-4135-01A-01R-1100-07	Tumor	LUSC	R0	Stage IB	M0	N0	T2a	Yes	Alive	MALE	WHITE	68	207

TCGA-37-4141-01A-02R-1100-07	Tumor	LUSC	R0	Stage IA	M0	N0	T1b	No	Alive	FEMALE	WHITE	65	12
TCGA-37-5819-01A-01R-1635-07	Tumor	LUSC	R0	Stage IIIA	M0	N2	T2	Yes	Alive	MALE	WHITE	64	103
TCGA-37-A5EL-01A-11R- A26W-07	Tumor	LUSC	Not Evaluated	Stage IIB	M0	N0	T3	Unknown	Alive	MALE	WHITE	53	1143
TCGA-37-A5EM-01A-21R-A27Q-07	Tumor	LUSC	Not Evaluated	Stage II	M0	N0	T2	Unknown	Alive	MALE	WHITE	49	867
TCGA-37-A5EN-01A-21R-A26W-07	Tumor	LUSC	Not Available	Stage IIIB	M0	N2	T4	Unknown	Alive	MALE	WHITE	59	660
TCGA-39-5011-01A-01R-1443-07	Tumor	LUSC	R0	Stage IA	M0	N0	T1b	Yes	Alive	FEMALE	WHITE	70	4053
TCGA-39-5016-01A-01R-1443-07	Tumor	LUSC	R0	Stage IIA	M0	N1	T2a	Yes	Alive	MALE	WHITE	44	3850
TCGA-39-5019-01A-01R-1820-07	Tumor	LUSC	R0	Stage IB	M0	N0	T2a	Yes	Alive	MALE	WHITE	70	3387

TCGA-39-5021-01A-01R-1443-07	Tumor	LUSC	R0	Stage IB	M0	N0	T2a	Yes	Dead	MALE	WHITE	70	2086
TCGA-39-5022-01A-21R-1820-07	Tumor	LUSC	R0	Stage IB	M0	N0	T2a	Yes	Dead	MALE	WHITE	76	1679
TCGA-39-5024-01A-21R-1820-07	Tumor	LUSC	R0	Stage IIIA	M0	N2	T2a	Yes	Alive	FEMALE	WHITE	65	2510
TCGA-39-5027-01A-21R-1820-07	Tumor	LUSC	R0	Stage IB	M0	N0	T2a	Yes	Alive	MALE	WHITE	73	3108
TCGA-39-5028-01A-01R-1443-07	Tumor	LUSC	R0	Stage IIIA	M0	N1	T4	Yes	Dead	MALE	WHITE	75	52
TCGA-39-5029-01A-01R-1443-07	Tumor	LUSC	R0	Stage IIIA	M0	N2	T1b	Yes	Dead	MALE	WHITE	67	740
TCGA-39-5030-01A-01R-1443-07	Tumor	LUSC	R0	Stage IIIA	M0	N2	T2a	Yes	Dead	FEMALE	ASIAN	81	59
TCGA-39-5031-01A-01R-1443-07	Tumor	LUSC	R0	Stage IA	M0	N0	T1a	Yes	Alive	FEMALE	Not Available	76	1841

TCGA-39-5034-01A-01R-1443-07	Tumor	LUSC	R0	Stage IIB	M0	N0	T3	Yes	Dead	FEMALE	WHITE	73	1107
TCGA-39-5035-01A-01R-1443-07	Tumor	LUSC	R0	Stage IA	M0	N0	T1b	Yes	Alive	FEMALE	WHITE	72	2080
TCGA-39-5036-01A-01R-1443-07	Tumor	LUSC	R0	Stage IB	M0	N0	T2	Yes	Alive	MALE	WHITE	73	2165
TCGA-39-5037-01A-01R-1443-07	Tumor	LUSC	R0	Stage IIA	M0	N1	T1b	Yes	Alive	MALE	WHITE	65	1690
TCGA-39-5039-01A-01R-1443-07	Tumor	LUSC	R0	Stage IIA	M0	N0	T2b	Yes	Dead	MALE	WHITE	76	544
TCGA-39-5040-01A-21R-2125-07	Tumor	LUSC	R0	Stage IIIA	M0	N2	T2a	Yes	Dead	MALE	WHITE	59	519
TCGA-39-5040-11A-01R-2125-07	Normal	LUSC	R0	Stage IIIA	M0	N2	T2a	Yes	Dead	MALE	WHITE	59	519
TCGA-43-2576-01A-01R- A32Z-07	Tumor	LUSC	R0	Stage IIIA	M0	N2	T2	Yes	Alive	FEMALE	WHITE	62	1223

TCGA-43-2578-01A-01R-0851-07	Tumor	LUSC	R0	Stage IA	M0	N0	T1	Yes	Alive	FEMALE	WHITE	59	684
TCGA-43-2581-01A-01R-0851-07	Tumor	LUSC	R0	Stage IIIA	M0	N1	T3	Yes	Alive	FEMALE	WHITE	47	1176
TCGA-43-3394-01A-01R-0980-07	Tumor	LUSC	R0	Stage IB	M0	N0	T2a	Yes	Alive	MALE	BLACK OR AFRICAN AMERICAN	52	1190
TCGA-43-3920-01A-01R-0980-07	Tumor	LUSC	R0	Stage IB	M0	N0	T2	Yes	Alive	MALE	WHITE	71	1007
TCGA-43-5668-01A-01R-1635-07	Tumor	LUSC	Not Available	Stage IIA	M0	N1	T1b	Yes	Alive	MALE	WHITE	78	559
TCGA-43-5670-01A-21R-2125-07	Tumor	LUSC	Not Available	Stage IIA	M0	N0	T2b	Yes	Alive	MALE	WHITE	70	849
TCGA-43-5670-11A-01R-2125-07	Normal	LUSC	Not Available	Stage IIA	M0	N0	T2b	Yes	Alive	MALE	WHITE	70	849

TCGA-43-6143-01A-11R-1820-07	Tumor	LUSC	RX	Stage IB	M0	N0	T2	Yes	Alive	MALE	WHITE	70	699
TCGA-43-6143-11A-01R-1820-07	Normal	LUSC	RX	Stage IB	M0	N0	T2	Yes	Alive	MALE	WHITE	70	699
TCGA-43-6647-01A-11R-1820-07	Tumor	LUSC	RX	Stage IIB	MX	N1	T2b	Yes	Alive	FEMALE	WHITE	69	757
TCGA-43-6647-11A-01R-1820-07	Normal	LUSC	RX	Stage IIB	MX	N1	T2b	Yes	Alive	FEMALE	WHITE	69	757
TCGA-43-6770-01A-11R-1820-07	Tumor	LUSC	RX	Stage IB	MX	N0	T2a	Yes	Alive	FEMALE	BLACK OR AFRICAN AMERICAN	59	653
TCGA-43-6771-01A-11R-1820-07	Tumor	LUSC	RX	Stage IB	MX	N0	T2	Yes	Dead	MALE	WHITE	85	166
TCGA-43-6771-11A-01R-1820-07	Normal	LUSC	RX	Stage IB	MX	N0	T2	Yes	Dead	MALE	WHITE	85	166

TCGA-43-6773-01A-41R-1949-07	Tumor	LUSC	Not Available	Stage IIB	MX	N1	T2	Yes	Dead	MALE	WHITE	76	116
TCGA-43-6773-11A-01R-1949-07	Normal	LUSC	Not Available	Stage IIB	MX	N1	T2	Yes	Dead	MALE	WHITE	76	116
TCGA-43-7656-01A-11R-2125-07	Tumor	LUSC	Not Available	Stage IA	MX	N0	T1b	Yes	Alive	MALE	WHITE	71	596
TCGA-43-7657-01A-31R-2125-07	Tumor	LUSC	Not Available	Stage IA	MX	N0	T1	Yes	Alive	FEMALE	WHITE	68	236
TCGA-43-7657-11A-01R-2125-07	Normal	LUSC	Not Available	Stage IA	MX	N0	T1	Yes	Alive	FEMALE	WHITE	68	236
TCGA-43-7658-01A-11R-2125-07	Tumor	LUSC	Not Available	Stage IA	M0	N0	T1	Yes	Alive	FEMALE	WHITE	75	2378
TCGA-43-7658-11A-01R-2125-07	Normal	LUSC	Not Available	Stage IA	M0	N0	T1	Yes	Alive	FEMALE	WHITE	75	2378
TCGA-43-8115-01A-11R-2247-07	Tumor	LUSC	R0	Stage IIA	MX	N1	T2a	Yes	Alive	FEMALE	WHITE	72	407

TCGA-43-8116-01A-11R-2247-07	Tumor	LUSC	Not Available	Stage IA	M0	N0	T1b	Yes	Alive	MALE	WHITE	73	358
TCGA-43-8118-01A-11R-2403-07	Tumor	LUSC	Not Evaluated	Stage IA	M0	N0	T1b	Yes	Dead	FEMALE	WHITE	55	89
TCGA-43-A474-01A-11R- A24H-07	Tumor	LUSC	R0	Stage IIA	M0	N0	T2b	Yes	Alive	MALE	WHITE	66	353
TCGA-43-A475-01A-11R- A24H-07	Tumor	LUSC	R1	Stage IIB	M0	N0	T3	Yes	Alive	FEMALE	WHITE	67	296
TCGA-43-A56U-01A-11R-A26W-07	Tumor	LUSC	R0	Stage IA	MX	N0	T1b	Yes	Alive	FEMALE	WHITE	76	432
TCGA-43-A56V-01A-11R- A26W-07	Tumor	LUSC	Not Available	Stage IIIA	M0	N2	T2a	Yes	Alive	MALE	BLACK OR AFRICAN AMERICAN	61	366
TCGA-46-3765-01A-01R-0980-07	Tumor	LUSC	R0	Stage IA	M0	N0	T1	Yes	Alive	FEMALE	WHITE	59	405

TCGA-46-3766-01A-01R-0980-07	Tumor	LUSC	R0	Stage IA	M0	N0	T1	Yes	Alive	FEMALE	WHITE	62	370
TCGA-46-3767-01A-01R-0980-07	Tumor	LUSC	R0	Stage IA	M0	N0	T1a	Yes	Alive	MALE	WHITE	76	396
TCGA-46-3768-01A-01R-0980-07	Tumor	LUSC	R0	Stage IIIA	M0	N1	T3	Yes	Dead	MALE	WHITE	58	299
TCGA-46-3769-01A-01R-0980-07	Tumor	LUSC	R1	Discrepancy	M0	N0	T4	Yes	Alive	MALE	WHITE	57	135
TCGA-46-6025-01A-11R-1820-07	Tumor	LUSC	R0	Stage IIB	M0	N1	T2b	Yes	Alive	MALE	WHITE	71	324
TCGA-46-6026-01A-11R-1820-07	Tumor	LUSC	R2	Stage IIB	M0	N1	T2a	Yes	Alive	MALE	WHITE	81	423
TCGA-51-4079-01A-01R-1100-07	Tumor	LUSC	R0	Stage IB	Not Available	N0	T2	Yes	Dead	FEMALE	BLACK OR AFRICAN AMERICAN	73	12
TCGA-51-4079-11A-01R-1758-07	Normal	LUSC	R0	Stage IB	Not Available	N0	T2	Yes	Dead	FEMALE	BLACK OR AFRICAN AMERICAN	73	12

TCGA-51-4080-01A-01R-1100-07	Tumor	LUSC	R0	Stage IIIB	Not Available	N1	T4	Yes	Dead	MALE	BLACK OR AFRICAN AMERICAN	65	12
TCGA-51-4081-01A-01R-1100-07	Tumor	LUSC	R0	Stage IIB	M0	N1	T2a	Yes	Alive	MALE	WHITE	55	911
TCGA-51-4081-11A-01R-1758-07	Normal	LUSC	R0	Stage IIB	M0	N1	T2a	Yes	Alive	MALE	WHITE	55	911
TCGA-51-6867-01A-11R-2045-07	Tumor	LUSC	R0	Stage I	M0	N0	T1	Yes	Dead	FEMALE	WHITE	72	1856
TCGA-52-7622-01A-11R-2125-07	Tumor	LUSC	R0	Stage IA	M0	N0	T1a	Yes	Alive	FEMALE	WHITE	62	862
TCGA-52-7809-01A-21R-2125-07	Tumor	LUSC	R0	Stage IB	M0	N0	T2	Yes	Dead	MALE	WHITE	74	166

TCGA-52-7810-01A-11R-2125-07	Tumor	LUSC	Not Available	Stage IIB	M0	N0	T3	Yes	Alive	FEMALE	WHITE	60	923
TCGA-52-7811-01A-11R-2125-07	Tumor	LUSC	R0	Stage IB	M0	N0	T2	Yes	Dead	MALE	WHITE	67	266
TCGA-52-7812-01A-11R-2125-07	Tumor	LUSC	R0	Discrepancy	M0	N2	T2	Yes	Dead	MALE	WHITE	68	835
TCGA-56-1622-01A-01R-0692-07	Tumor	LUSC	R0	Stage IB	M0	N0	T2	Yes	Dead	MALE	WHITE	58	881
TCGA-56-5897-01A-11R-1635-07	Tumor	LUSC	Not Available	Stage IA	MX	N0	T1b	Yes	Alive	MALE	WHITE	74	378
TCGA-56-5898-01A-11R-1635-07	Tumor	LUSC	R0	Stage IA	M0	N0	T1b	Yes	Alive	MALE	WHITE	69	555
TCGA-56-6545-01A-11R-1820-07	Tumor	LUSC	R0	Stage IB	M0	N0	T2a	Yes	Alive	FEMALE	WHITE	77	666
TCGA-56-6546-01A-11R-1820-07	Tumor	LUSC	Not Available	Stage IIA	MX	N0	T2b	Yes	Alive	MALE	WHITE	67	

TCGA-56-7221-01A-11R-2045-07	Tumor	LUSC	R0	Stage IB	M0	N0	T2	Yes	Alive	MALE	WHITE	79	608
TCGA-56-7222-01A-11R-2045-07	Tumor	LUSC	Not Available	Stage IB	M0	N0	T2a	Yes	Alive	MALE	WHITE	60	562
TCGA-56-7222-11A-01R-2045-07	Normal	LUSC	Not Available	Stage IB	M0	N0	T2a	Yes	Alive	MALE	WHITE	60	562
TCGA-56-7223-01A-11R-2045-07	Tumor	LUSC	R0	Stage IIIA	MX	N1	T3	Yes	Alive	MALE	WHITE	66	442
TCGA-56-7579-01A-11R-2045-07	Tumor	LUSC	R0	Stage IIIA	M0	N1	T3	Yes	Alive	MALE	WHITE	61	951
TCGA-56-7579-11A-01R-2045-07	Normal	LUSC	R0	Stage IIIA	M0	N1	T3	Yes	Alive	MALE	WHITE	61	951
TCGA-56-7580-01A-11R-2045-07	Tumor	LUSC	R0	Stage IB	M0	N0	T2a	Yes	Alive	MALE	WHITE	84	925
TCGA-56-7580-11A-01R-2045-07	Normal	LUSC	R0	Stage IB	M0	N0	T2a	Yes	Alive	MALE	WHITE	84	925

TCGA-56-7582-01A-11R-2045-07	Tumor	LUSC	R0	Stage IB	M0	N0	T2a	Yes	Alive	MALE	WHITE	83	601
TCGA-56-7582-11A-01R-2045-07	Normal	LUSC	R0	Stage IB	M0	N0	T2a	Yes	Alive	MALE	WHITE	83	601
TCGA-56-7730-01A-11R-2125-07	Tumor	LUSC	R1	Stage IIA	M0	N0	T2b	Yes	Alive	MALE	WHITE	73	198
TCGA-56-7730-11A-01R-2125-07	Normal	LUSC	R1	Stage IIA	M0	N0	T2b	Yes	Alive	MALE	WHITE	73	198
TCGA-56-7731-01A-11R-2125-07	Tumor	LUSC	Not Available	Stage IB	MX	N0	T2a	Yes	Alive	FEMALE	WHITE	66	3
TCGA-56-7731-11A-01R-2125-07	Normal	LUSC	Not Available	Stage IB	MX	N0	T2a	Yes	Alive	FEMALE	WHITE	66	3
TCGA-56-7822-01A-11R-2125-07	Tumor	LUSC	R0	Stage IIB	M0	N1	T2b	Yes	Alive	MALE	BLACK OR AFRICAN AMERICAN	75	532

TCGA-56-7823-01B-11R-2247-07	Tumor	LUSC	Not Available	Stage IIA	M0	N1	T1b	Yes	Alive	FEMALE	WHITE	58	1011
TCGA-56-7823-11A-01R-2247-07	Normal	LUSC	Not Available	Stage IIA	M0	N1	T1b	Yes	Alive	FEMALE	WHITE	58	1011
TCGA-56-8082-01A-11R-2247-07	Tumor	LUSC	R0	Stage IIA	MX	N0	T2b	Yes	Alive	FEMALE	WHITE	80	455
TCGA-56-8082-11A-01R-2247-07	Normal	LUSC	R0	Stage IIA	MX	N0	T2b	Yes	Alive	FEMALE	WHITE	80	455
TCGA-56-8083-01A-11R-2247-07	Tumor	LUSC	R0	Stage IB	MX	N0	T2a	Yes	Alive	MALE	WHITE	56	150
TCGA-56-8083-11A-01R-2247-07	Normal	LUSC	R0	Stage IB	MX	N0	T2a	Yes	Alive	MALE	WHITE	56	150
TCGA-56-8201-01A-11R-2247-07	Tumor	LUSC	R0	Stage IIB	MX	N0	T3	Yes	Alive	MALE	WHITE	74	397

TCGA-56-8201-11A-01R-2247-07	Normal	LUSC	R0	Stage IIB	MX	N0	T3	Yes	Alive	MALE	WHITE	74	397
TCGA-56-8304-01A-11R-2326-07	Tumor	LUSC	R0	Stage IA	MX	N0	T1b	Yes	Alive	FEMALE	WHITE	73	106
TCGA-56-8305-01A-11R-2296-07	Tumor	LUSC	R0	Stage IB	M0	N0	T2a	Yes	Alive	MALE	WHITE	72	105
TCGA-56-8307-01A-11R-2296-07	Tumor	LUSC	R0	Stage IIB	M0	N0	T3	Yes	Alive	FEMALE	WHITE	55	818
TCGA-56-8308-01A-11R-2296-07	Tumor	LUSC	R1	Stage IIB	MX	N0	T3	Yes	Alive	MALE	WHITE	79	517
TCGA-56-8309-01A-11R-2296-07	Tumor	LUSC	R0	Stage IA	MX	N0	T1b	Yes	Alive	MALE	WHITE	66	428
TCGA-56-8309-11A-01R-2296-07	Normal	LUSC	R0	Stage IA	MX	N0	T1b	Yes	Alive	MALE	WHITE	66	428
TCGA-56-8503-01A-11R-2403-07	Tumor	LUSC	R0	Stage IIB	M0	N0	T3	Yes	Alive	FEMALE	WHITE	76	41

TCGA-56-8504-01A-11R-2403-07	Tumor	LUSC	R0	Stage IB	MX	N0	T2a	Yes	Alive	MALE	WHITE	74	510
TCGA-56-8622-01A-11R-2403-07	Tumor	LUSC	R0	Stage IB	M0	N0	T2a	Yes	Alive	MALE	WHITE	68	55
TCGA-56-8623-01A-11R- A28V-07	Tumor	LUSC	R0	Stage IB	MX	N0	T2a	Yes	Alive	MALE	WHITE	71	692
TCGA-56-8623-11A-01R- A28V-07	Normal	LUSC	R0	Stage IB	MX	N0	T2a	Yes	Alive	MALE	WHITE	71	692
TCGA-56-8624-01A-11R-2403-07	Tumor	LUSC	R0	Stage IIB	MX	N0	T3	Yes	Alive	MALE	WHITE	84	420
TCGA-56-8625-01A-11R-2403-07	Tumor	LUSC	RX	Stage IIIA	MX	N1	T3	Yes	Alive	FEMALE	WHITE	66	315
TCGA-56-8626-01A-11R-2403-07	Tumor	LUSC	R0	Stage IA	MX	N0	T1a	Yes	Alive	MALE	WHITE	59	302
TCGA-56-8628-01A-11R-2403-07	Tumor	LUSC	R0	Stage IA	MX	N0	T1b	Yes	Alive	MALE	WHITE	78	616

TCGA-56-8629-01A-11R-2403-07	Tumor	LUSC	R0	Stage IIA	MX	N0	T2b	Yes	Alive	MALE	WHITE	63	481
TCGA-56-A49D-01A-11R-A24H-07	Tumor	LUSC	R0	Stage IIIA	MX	N2	T2a	Yes	Alive	MALE	WHITE	67	637
TCGA-56-A4BW-01A-11R-A24H-07	Tumor	LUSC	R0	Stage IIA	M0	N1	T2a	Yes	Alive	MALE	WHITE	55	585
TCGA-56-A4BX-01A-11R-A24H-07	Tumor	LUSC	R0	Stage IIA	MX	N0	T2b	Yes	Alive	MALE	WHITE	70	405
TCGA-56-A4BY-01A-11R- A24H-07	Tumor	LUSC	RX	Stage IB	MX	N0	T2a	Yes	Alive	MALE	WHITE	66	543
TCGA-56-A4ZJ-01A-11R- A262-07	Tumor	LUSC	R0	Stage IA	M0	N0	T1a	Yes	Alive	FEMALE	WHITE	75	640
TCGA-56-A4ZK-01A-11R-A262-07	Tumor	LUSC	R1	Stage IB	M0	N0	T2	Yes	Alive	FEMALE	WHITE	76	570
TCGA-56-A5DR-01A-11R-A27Q-07	Tumor	LUSC	R0	Stage IA	MX	N0	T1a	No	Alive	MALE	WHITE	81	4

TCGA-56-A5DS-01A-11R-A27Q-07	Tumor	LUSC	R0	Stage IB	MX	N0	T2a	Yes	Alive	FEMALE	WHITE	72	8
TCGA-56-A62T-01A-11R- A405-07	Tumor	LUSC	R1	Stage IIA	MX	N0	T2b	Yes	Alive	MALE	BLACK OR AFRICAN AMERICAN	78	440
TCGA-58-8386-01A-11R-2296-07	Tumor	LUSC	R0	Stage IV	M1	NX	T3	Yes	Dead	MALE	Not Evaluated	75	1
TCGA-58-8386-11A-01R-2296-07	Normal	LUSC	R0	Stage IV	M1	NX	T3	Yes	Dead	MALE	Not Evaluated	75	1
TCGA-58-8387-01A-11R-2296-07	Tumor	LUSC	R0	Stage IIA	M0	N0	T2b	Yes	Dead	MALE	WHITE	60	403
TCGA-58-8388-01A-11R-2326-07	Tumor	LUSC	R0	Stage IB	M0	N0	T2a	Yes	Alive	MALE	WHITE	60	412
TCGA-58-8390-01A-11R-2326-07	Tumor	LUSC	R0	Stage IIA	M0	N0	T2b	Yes	Alive	MALE	WHITE	70	911

TCGA-58-8391-01A-11R-2326-07	Tumor	LUSC	R0	Stage IIIA	M0	N2	T2	Yes	Alive	FEMALE	WHITE	57	2167
TCGA-58-8392-01A-11R-2326-07	Tumor	LUSC	R0	Stage IB	M0	N0	T2a	Yes	Dead	MALE	WHITE	70	501
TCGA-58-8393-01A-11R-2326-07	Tumor	LUSC	R0	Stage IB	M0	N0	T2a	Yes	Alive	FEMALE	WHITE	68	1058
TCGA-58-A46J-01A-11R- A24H-07	Tumor	LUSC	R0	Stage IIB	M0	N1	T2	Yes	Alive	MALE	WHITE	64	2589
TCGA-58-A46K-01A-11R- A24H-07	Tumor	LUSC	R0	Stage IIIA	M0	N2	T2	Yes	Dead	MALE	WHITE	59	1045
TCGA-58-A46L-01A-11R- A24H-07	Tumor	LUSC	R0	Stage IIIA	M0	N2	T2	Yes	Alive	MALE	WHITE	73	1723
TCGA-58-A46M-01A-11R-A24H-07	Tumor	LUSC	R0	Stage IIB	M0	N1	T2b	Yes	Alive	MALE	WHITE	61	1072
TCGA-58-A46N-01A-11R-A24H-07	Tumor	LUSC	R0	Stage IB	M0	N0	T2a	Yes	Alive	MALE	WHITE	52	910

TCGA-60-2695-01A-01R-0851-07	Tumor	LUSC	R0	Stage IB	M0	N0	T2	Yes	Alive	FEMALE	WHITE	74	642
TCGA-60-2696-01A-01R-0851-07	Tumor	LUSC	RX	Stage IIA	M0	N0	T2	Yes	Dead	FEMALE	BLACK OR AFRICAN AMERICAN	76	109
TCGA-60-2697-01A-11R-2125-07	Tumor	LUSC	RX	Stage IIA	M0	N2	T2	Not Available	Dead	MALE	WHITE	41	372
TCGA-60-2698-01A-01R-0851-07	Tumor	LUSC	RX	Stage IIB	M0	N1	T2	Yes	Dead	MALE	WHITE	62	311
TCGA-60-2703-01A-11R-2045-07	Tumor	LUSC	R0	Stage IIB	M0	N1	T2	Yes	Dead	MALE	WHITE	73	2945
TCGA-60-2704-01A-11R-2045-07	Tumor	LUSC	R0	Stage IIB	M0	N1	T2	Yes	Dead	MALE	WHITE	73	1154
TCGA-60-2706-01A-01R-0851-07	Tumor	LUSC	Not Available	Stage IA	M0	N0	T1	Yes	Alive	MALE	WHITE	58	2820

TCGA-60-2707-01A-01R-0851-07	Tumor	LUSC	RX	Stage IB	M0	N0	T2	Yes	Dead	MALE	Not Available	70	667
TCGA-60-2708-01A-01R-0851-07	Tumor	LUSC	R0	Stage IIB	M0	N1	T2	Yes	Alive	FEMALE	WHITE	64	2447
TCGA-60-2709-01A-21R-1820-07	Tumor	LUSC	R0	Stage IB	MX	N0	T2	Yes	Alive	MALE	BLACK OR AFRICAN AMERICAN	69	1505
TCGA-60-2709-11A-01R-1820-07	Normal	LUSC	R0	Stage IB	MX	N0	T2	Yes	Alive	MALE	BLACK OR AFRICAN AMERICAN	69	1505
TCGA-60-2710-01A-01R-0851-07	Tumor	LUSC	Not Available	Stage IIA	M0	N1	T1	Yes	Alive	FEMALE	WHITE	67	2024
TCGA-60-2711-01A-01R-0851-07	Tumor	LUSC	R0	Stage IB	M0	N0	T2	Yes	Alive	FEMALE	WHITE	64	1260

TCGA-60-2712-01A-01R-0851-07	Tumor	LUSC	R0	Stage IIB	M0	N1	T2	Yes	Dead	FEMALE	WHITE	79	274
TCGA-60-2713-01A-01R-0851-07	Tumor	LUSC	R0	Stage IB	M0	N0	T2	Yes	Alive	MALE	WHITE	64	1731
TCGA-60-2714-01A-01R-0851-07	Tumor	LUSC	R0	Stage IIB	M0	N1	T2	Yes	Alive	FEMALE	WHITE	66	1531
TCGA-60-2715-01A-01R-0851-07	Tumor	LUSC	RX	Stage IA	M0	N0	T1	Yes	Dead	MALE	WHITE	51	1075
TCGA-60-2716-01A-01R-0851-07	Tumor	LUSC	R0	Stage IIB	M0	N1	T2	No	Alive	MALE	Not Available	39	1475
TCGA-60-2719-01A-01R-0851-07	Tumor	LUSC	R0	Stage IB	M0	N0	T1	Yes	Alive	FEMALE	WHITE	83	1297
TCGA-60-2720-01A-01R-0851-07	Tumor	LUSC	R0	Stage IB	M0	N0	T2	Yes	Alive	FEMALE	WHITE	60	97
TCGA-60-2721-01A-01R-0851-07	Tumor	LUSC	R0	Stage IB	M0	N0	T2	Yes	Alive	MALE	WHITE	73	983

TCGA-60-2722-01A-01R-0851-07	Tumor	LUSC	R0	Stage IIB	M0	N1	T2	Yes	Alive	MALE	WHITE	66	908
TCGA-60-2723-01A-01R-0851-07	Tumor	LUSC	R0	Stage IB	M0	N0	T2	Yes	Alive	FEMALE	WHITE	74	1092
TCGA-60-2724-01A-01R-0851-07	Tumor	LUSC	R0	Stage IIIA	M0	N1	T3	Yes	Alive	MALE	WHITE	47	717
TCGA-60-2725-01A-01R-1201-07	Tumor	LUSC	R0	Stage IB	M0	N0	T2	Yes	Alive	MALE	WHITE	74	816
TCGA-60-2726-01A-01R-0851-07	Tumor	LUSC	R0	Stage IIA	M0	N1	T2	Yes	Dead	MALE	WHITE	56	358
TCGA-63-5128-01A-01R-1443-07	Tumor	LUSC	R0	Stage IB	M0	N0	T2	Yes	Dead	MALE	Not Available	Not Available	
TCGA-63-5131-01A-01R-1443-07	Tumor	LUSC	R0	Stage IIB	M0	N1	T2	Yes	Dead	MALE	Not Available	Not Available	
TCGA-63-6202-01A-11R-1820-07	Tumor	LUSC	R0	Stage IIA	M0	N0	T2	Yes	Alive	MALE	Not Available	Not Available	1602

TCGA-63-7020-01A-11R-1949-07	Tumor	LUSC	R0	Stage IA	M0	N0	T1	Yes	Alive	MALE	Not Available	Not Available	2133
TCGA-63-7021-01A-11R-1949-07	Tumor	LUSC	R0	Stage IA	M0	N0	T1	Yes	Alive	MALE	Not Available	Not Available	2142
TCGA-63-7022-01A-11R-1949-07	Tumor	LUSC	R0	Stage IA	M0	N0	T1	Yes	Alive	FEMALE	Not Available	Not Available	2073
TCGA-63-7023-01A-11R-1949-07	Tumor	LUSC	R0	Stage IIA	M0	N1	T1	Yes	Alive	MALE	Not Available	Not Available	1682
TCGA-63-A5M9-01A-11R-A26W-07	Tumor	LUSC	RX	Stage IIB	M0	N1	T2	Yes	Alive	FEMALE	Not Evaluated	Not Available	0
TCGA-63-A5MB-01A-11R-A26W-07	Tumor	LUSC	R0	Stage IB	M0	N0	T2	Yes	Alive	MALE	Not Evaluated	62	3123
TCGA-63-A5MG-01A-12R-A27Q-07	Tumor	LUSC	R0	Stage IB	M0	N0	T2	Yes	Alive	MALE	Not Evaluated	68	2148
TCGA-63-A5MH-01A-12R-A27Q-07	Tumor	LUSC	R0	Stage IA	M0	N0	T1	Yes	Alive	MALE	Not Evaluated	68	2026

TCGA-63-A5MI-01A-12R- A27Q-07	Tumor	LUSC	R0	Stage IIIA	M0	N2	T2	Yes	Alive	MALE	Not Evaluated	65	1784
TCGA-63-A5MJ-01A-11R-A27Q-07	Tumor	LUSC	R0	Stage IIB	M0	N1	T2	Yes	Alive	MALE	Not Evaluated	54	1824
TCGA-63-A5ML-01A-31R-A27Q-07	Tumor	LUSC	R0	Stage IB	M0	N0	T2	Yes	Alive	MALE	Not Evaluated	68	1386
TCGA-63-A5MM-01A-11R-A26W-07	Tumor	LUSC	R0	Stage IIB	M0	N1	T2	Yes	Dead	FEMALE	Not Evaluated	69	456
TCGA-63-A5MN-01A-22R-A27Q-07	Tumor	LUSC	R1	Stage IIB	M0	N0	T3	No	Dead	FEMALE	Not Evaluated	78	345
TCGA-63-A5MP-01A-11R-A26W-07	Tumor	LUSC	R0	Stage IIB	M0	N1	T2	Yes	Alive	MALE	Not Evaluated	56	769
TCGA-63-A5MR-01A-31R-A27Q-07	Tumor	LUSC	R0	Stage IB	M0	N0	T2	Yes	Alive	FEMALE	Not Evaluated	70	2716
TCGA-63-A5MS-01A-11R-A26W-07	Tumor	LUSC	R0	Stage IB	M0	N0	T2	Yes	Alive	MALE	Not Evaluated	78	2381

TCGA-63-A5MT-01A-21R-A26W-07	Tumor	LUSC	R0	Stage IIB	M0	N0	T3	Yes	Alive	MALE	Not Evaluated	74	498
TCGA-63-A5MU-01A-11R-A26W-07	Tumor	LUSC	R0	Stage IIB	M0	N1	T2b	Yes	Dead	MALE	Not Evaluated	48	
TCGA-63-A5MV-01A-21R-A26W-07	Tumor	LUSC	R0	Stage IIA	M0	N0	T2b	Yes	Alive	MALE	Not Evaluated	69	1100
TCGA-63-A5MW-01A-11R-A26W-07	Tumor	LUSC	R0	Stage IB	M0	N0	T2	Yes	Alive	MALE	Not Evaluated	76	1639
TCGA-63-A5MY-01A-11R-A26W-07	Tumor	LUSC	R0	Stage IA	M0	N0	T1b	Yes	Alive	MALE	Not Evaluated	63	1052
TCGA-66-2727-01A-01R-0980-07	Tumor	LUSC	R0	Stage IB	M0	N0	T2	Yes	Dead	FEMALE	Not Available	55	516
TCGA-66-2734-01A-01R-0980-07	Tumor	LUSC	R0	Stage IB	M0	N0	T2	Yes	Alive	FEMALE	Not Available	62	1311
TCGA-66-2737-01A-01R-0980-07	Tumor	LUSC	R0	Stage IIB	M0	N1	T2	Yes	Alive	MALE	Not Available	72	61

TCGA-66-2742-01A-01R-0980-07	Tumor	LUSC	R0	Stage IV	M1	N1	T2	Yes	Alive	MALE	Not Available	70	641
TCGA-66-2744-01A-01R-0980-07	Tumor	LUSC	R0	Stage IIB	M0	N1	T2	Yes	Alive	MALE	Not Available	71	30
TCGA-66-2753-01A-01R-0980-07	Tumor	LUSC	R0	Stage IB	M0	N0	T2	Yes	Alive	MALE	Not Available	69	31
TCGA-66-2754-01A-01R-0980-07	Tumor	LUSC	R0	Stage IIIA	M0	N2	T2	Yes	Alive	MALE	Not Available	67	61
TCGA-66-2755-01A-01R-0851-07	Tumor	LUSC	R0	Stage IB	M0	N0	T2	Yes	Alive	MALE	Not Available	63	28
TCGA-66-2756-01A-01R-0851-07	Tumor	LUSC	R0	Stage IIIB	M0	N0	T4	Yes	Alive	MALE	Not Available	68	30
TCGA-66-2757-01A-01R-0851-07	Tumor	LUSC	R0	Stage IA	M0	N0	T1	Yes	Alive	FEMALE	Not Available	65	1338
TCGA-66-2758-01A-02R-0851-07	Tumor	LUSC	R0	Stage IB	M0	N0	T2	Yes	Alive	MALE	Not Available	71	639

TCGA-66-2759-01A-01R-0851-07	Tumor	LUSC	R0	Stage IIIA	M0	N2	T2	Yes	Alive	MALE	Not Available	66	762
TCGA-66-2763-01A-01R-0851-07	Tumor	LUSC	R0	Stage IB	M0	N0	T2	Yes	Alive	FEMALE	Not Available	63	30
TCGA-66-2765-01A-01R-0851-07	Tumor	LUSC	R0	Stage IB	M0	N0	T2	Yes	Alive	MALE	Not Available	64	61
TCGA-66-2766-01A-01R-0851-07	Tumor	LUSC	R0	Stage IIIA	M0	N2	T2	Yes	Alive	MALE	Not Available	54	31
TCGA-66-2767-01A-01R-0851-07	Tumor	LUSC	R0	Stage IIIB	M0	N3	T2	Yes	Alive	MALE	Not Available	62	61
TCGA-66-2768-01A-01R-0851-07	Tumor	LUSC	R0	Stage IIB	M0	N1	T2	Yes	Alive	MALE	Not Available	57	61
TCGA-66-2769-01A-02R-0851-07	Tumor	LUSC	R1	Stage IIIB	M0	N0	T4	Yes	Dead	MALE	Not Available	75	215
TCGA-66-2770-01A-01R-0851-07	Tumor	LUSC	R0	Stage IB	M0	N0	T2	Yes	Alive	MALE	Not Available	79	700

TCGA-66-2771-01A-01R-0980-07	Tumor	LUSC	R0	Stage IIB	M0	N1	T2	Yes	Alive	MALE	Not Available	60	578
TCGA-66-2773-01A-01R-1201-07	Tumor	LUSC	R0	Stage IB	M0	N0	T2	Yes	Dead	MALE	Not Available	69	92
TCGA-66-2777-01A-01R-1201-07	Tumor	LUSC	R0	Stage IB	M0	N0	T2	Yes	Alive	MALE	Not Available	71	61
TCGA-66-2778-01A-02R-0851-07	Tumor	LUSC	R2	Stage IIIB	M0	N3	T2	Yes	Alive	FEMALE	Not Available	68	578
TCGA-66-2780-01A-01R-0851-07	Tumor	LUSC	R0	Stage IB	M0	N0	T2	Yes	Dead	MALE	Not Available	65	366
TCGA-66-2781-01A-01R-0851-07	Tumor	LUSC	R0	Stage IB	M0	N0	T2	Yes	Alive	MALE	Not Available	67	121
TCGA-66-2782-01A-01R-0851-07	Tumor	LUSC	R0	Stage IIB	M0	N0	T3	Yes	Dead	MALE	Not Available	71	365
TCGA-66-2783-01A-01R-1201-07	Tumor	LUSC	R2	Stage IIIB	M0	N3	T2	Yes	Alive	MALE	Not Available	67	759

TCGA-66-2785-01A-01R-0851-07	Tumor	LUSC	R0	Stage IB	M0	N0	T2	Yes	Alive	MALE	Not Available	65	60
TCGA-66-2786-01A-01R-0851-07	Tumor	LUSC	R0	Stage IA	M0	N0	T1	Yes	Alive	FEMALE	Not Available	68	790
TCGA-66-2787-01A-01R-0980-07	Tumor	LUSC	R0	Stage IA	M0	N0	T1	Yes	Alive	MALE	Not Available	57	1217
TCGA-66-2788-01A-01R-0980-07	Tumor	LUSC	R0	Stage IB	M0	N0	T2	Yes	Alive	MALE	Not Available	56	699
TCGA-66-2789-01A-01R-0980-07	Tumor	LUSC	R0	Stage IIIB	M0	N3	T1	Yes	Dead	MALE	Not Available	73	123
TCGA-66-2790-01A-01R-0980-07	Tumor	LUSC	R0	Stage IIB	M0	N1	T2	Yes	Alive	MALE	Not Available	72	699
TCGA-66-2791-01A-01R-0980-07	Tumor	LUSC	R1	Stage IIIB	M0	N3	T2	Yes	Dead	MALE	Not Available	66	153
TCGA-66-2792-01A-01R-0980-07	Tumor	LUSC	R0	Stage IIB	M0	N1	T2	Yes	Alive	MALE	Not Available	58	913

TCGA-66-2793-01A-01R-1201-07	Tumor	LUSC	R0	Stage IIIB	M0	N1	T4	Yes	Dead	MALE	Not Available	68	306
TCGA-66-2794-01A-01R-1201-07	Tumor	LUSC	R0	Stage IIIB	M0	N2	T4	Yes	Alive	MALE	Not Available	64	1645
TCGA-66-2795-01A-02R-0980-07	Tumor	LUSC	R0	Stage IIIB	M0	N1	T4	Yes	Alive	MALE	Not Available	68	122
TCGA-66-2800-01A-01R-1201-07	Tumor	LUSC	R0	Stage IIIB	M0	N0	T4	Yes	Alive	MALE	Not Available	70	1492
TCGA-68-7755-01A-11R-2125-07	Tumor	LUSC	Not Available	Stage IIA	M0	N1	T1b	Yes	Alive	FEMALE	WHITE	60	83
TCGA-68-7756-01A-11R-2125-07	Tumor	LUSC	Not Available	Stage IIIA	MX	N1	T4	Yes	Alive	MALE	WHITE	84	202
TCGA-68-7757-01B-11R-2296-07	Tumor	LUSC	Not Available	Stage IA	MX	N0	T1b	Yes	Alive	MALE	WHITE	74	211

TCGA-68-8250-01A-11R-2296-07	Tumor	LUSC	Not Available	Stage IA	MX	N0	T1a	Yes	Alive	MALE	BLACK OR AFRICAN AMERICAN	66	244
TCGA-68-8251-01A-11R-2296-07	Tumor	LUSC	Not Available	Stage IB	M0	N0	T2a	Yes	Alive	MALE	WHITE	78	406
TCGA-68-A59I- 01A-11R-A262-07	Tumor	LUSC	Not Evaluated	Stage IIIA	M0	N1	T3	Yes	Alive	FEMALE	BLACK OR AFRICAN AMERICAN	73	492
TCGA-68-A59J-01A-21R- A26W-07	Tumor	LUSC	R0	Stage IB	MX	N0	T2a	Yes	Alive	FEMALE	WHITE	74	448
TCGA-6A-AB49-01A-12R-A405-07	Tumor	LUSC	RX	Stage IB	MX	N0	T2	Yes	Dead	FEMALE	BLACK OR AFRICAN AMERICAN	73	

TCGA-70-6722-01A-11R-1820-07	Tumor	LUSC	RX	Stage IIIA	M0	N1	T3	No	Alive	MALE	ASIAN	47	367
TCGA-70-6723-01A-11R-1820-07	Tumor	LUSC	RX	Stage IIA	M0	N0	T3	No	Alive	MALE	ASIAN	65	375
TCGA-77-6842-01A-11R-1949-07	Tumor	LUSC	R0	Stage IIB	M0	N1	T2	Yes	Dead	MALE	WHITE	79	899
TCGA-77-6843-01A-11R-1949-07	Tumor	LUSC	R0	Stage IIA	M0	N1	T1	Yes	Dead	MALE	WHITE	74	2224
TCGA-77-6844-01A-11R-1949-07	Tumor	LUSC	R0	Stage IIIA	M0	N1	T3	Yes	Dead	MALE	WHITE	74	2284
TCGA-77-6845-01A-11R-1949-07	Tumor	LUSC	R0	Stage IIB	M0	N0	T3	Yes	Dead	MALE	WHITE	69	708
TCGA-77-7138-01A-41R-2045-07	Tumor	LUSC	R0	Stage IB	M0	N0	T2	Yes	Dead	MALE	Not Available	67	340

TCGA-77-7138-11A-01R-2045-07	Normal	LUSC	R0	Stage IB	M0	N0	T2	Yes	Dead	MALE	Not Available	67	340
TCGA-77-7139-01A-11R-2045-07	Tumor	LUSC	R0	Stage IIB	M0	N1	T2	Yes	Alive	MALE	WHITE	56	4261
TCGA-77-7140-01A-41R-2045-07	Tumor	LUSC	R0	Stage IIB	M0	N1	T2	No	Dead	FEMALE	WHITE	69	632
TCGA-77-7141-01A-11R-2045-07	Tumor	LUSC	R0	Stage IB	M0	N0	T2	Yes	Alive	MALE	WHITE	64	15
TCGA-77-7142-01A-11R-2045-07	Tumor	LUSC	R0	Stage IB	M0	N0	T2	Yes	Alive	FEMALE	WHITE	59	2227
TCGA-77-7142-11A-01R-2045-07	Normal	LUSC	R0	Stage IB	M0	N0	T2	Yes	Alive	FEMALE	WHITE	59	2227
TCGA-77-7335-01A-11R-2045-07	Tumor	LUSC	R0	Stage IIIB	M0	N2	T4	Yes	Dead	FEMALE	WHITE	62	2133
TCGA-77-7335-11A-01R-2045-07	Normal	LUSC	R0	Stage IIIB	M0	N2	T4	Yes	Dead	FEMALE	WHITE	62	2133

TCGA-77-7337-01A-21R-2045-07	Tumor	LUSC	R0	Stage IIB	M0	N1	T2	Yes	Dead	MALE	WHITE	65	3253
TCGA-77-7337-11A-01R-2045-07	Normal	LUSC	R0	Stage IIB	M0	N1	T2	Yes	Dead	MALE	WHITE	65	3253
TCGA-77-7338-01A-11R-2045-07	Tumor	LUSC	R1	Stage IB	M0	N0	T2	Yes	Dead	MALE	WHITE	64	5
TCGA-77-7338-11A-01R-2045-07	Normal	LUSC	R1	Stage IB	M0	N0	T2	Yes	Dead	MALE	WHITE	64	5
TCGA-77-7463-01A-11R-2045-07	Tumor	LUSC	R0	Stage IB	M0	N0	T2	Yes	Dead	MALE	WHITE	75	1423
TCGA-77-7465-01A-11R-2045-07	Tumor	LUSC	R0	Stage IIA	M0	N1	T2a	Yes	Alive	MALE	WHITE	58	990
TCGA-77-8007-01A-11R-2187-07	Tumor	LUSC	R0	Stage IIB	M0	N1	T2	Yes	Dead	MALE	Not Available	68	198
TCGA-77-8007-11A-01R-2187-07	Normal	LUSC	R0	Stage IIB	M0	N1	T2	Yes	Dead	MALE	Not Available	68	198

TCGA-77-8008-01A-21R-2187-07	Tumor	LUSC	R0	Stage IB	M0	N0	T2	Yes	Dead	MALE	Not Available	68	2639
TCGA-77-8008-11A-01R-2187-07	Normal	LUSC	R0	Stage IB	M0	N0	T2	Yes	Dead	MALE	Not Available	68	2639
TCGA-77-8009-01A-11R-2187-07	Tumor	LUSC	R0	Stage IIB	M0	N1	T2	Yes	Alive	MALE	Not Available	68	1695
TCGA-77-8128-01A-11R-2247-07	Tumor	LUSC	R0	Stage IIIA	M0	N2	T2	Yes	Dead	MALE	Not Evaluated	60	1150
TCGA-77-8130-01A-11R-2247-07	Tumor	LUSC	R0	Stage IIB	M0	N1	T2	Yes	Alive	MALE	Not Evaluated	69	4765
TCGA-77-8131-01A-11R-2247-07	Tumor	LUSC	R0	Stage IB	M0	NX	T2	Yes	Dead	MALE	Not Evaluated	72	383
TCGA-77-8133-01A-12R-2247-07	Tumor	LUSC	R0	Stage IIA	M0	N1	T1	Yes	Dead	MALE	Not Evaluated	74	1640
TCGA-77-8136-01A-11R-2247-07	Tumor	LUSC	R0	Stage IIB	M0	N1	T2	Yes	Dead	FEMALE	Not Evaluated	74	1189

TCGA-77-8138-01A-11R-2247-07	Tumor	LUSC	R0	Stage IB	M0	N0	T2	Yes	Dead	MALE	Not Evaluated	74	539
TCGA-77-8139-01A-11R-2247-07	Tumor	LUSC	R0	Stage IIB	M0	N0	T3	Yes	Alive	MALE	Not Evaluated	72	3166
TCGA-77-8140-01A-11R-2247-07	Tumor	LUSC	R0	Stage IIB	M0	N1	T2	Yes	Dead	FEMALE	Not Evaluated	66	351
TCGA-77-8143-01A-11R-2247-07	Tumor	LUSC	R0	Stage IIIA	M0	N2	T2	Yes	Dead	MALE	Not Evaluated	76	803
TCGA-77-8144-01A-11R-2247-07	Tumor	LUSC	R0	Stage IB	M0	N0	T2	Yes	Alive	MALE	Not Evaluated	70	833
TCGA-77-8145-01A-11R-2247-07	Tumor	LUSC	R1	Stage IIIB	M0	N1	T4	Yes	Dead	MALE	Not Evaluated	73	212
TCGA-77-8146-01A-11R-2247-07	Tumor	LUSC	R0	Stage IA	M0	N0	T1	Yes	Alive	MALE	Not Evaluated	72	3189
TCGA-77-8148-01A-11R-2247-07	Tumor	LUSC	R0	Stage IIIA	M0	N1	T3	Yes	Alive	MALE	Not Evaluated	68	2023

TCGA-77-8150-01A-11R-2247-07	Tumor	LUSC	R0	Stage IIIA	M0	N1	T3	Yes	Dead	MALE	Not Evaluated	64	1655
TCGA-77-8153-01A-11R-2403-07	Tumor	LUSC	R0	Stage IB	M0	N0	T2	Yes	Alive	FEMALE	Not Evaluated	77	1992
TCGA-77-8154-01A-11R-2247-07	Tumor	LUSC	R0	Stage IA	M0	N0	T1	Yes	Alive	MALE	Not Evaluated	67	1841
TCGA-77-8156-01A-11R-2247-07	Tumor	LUSC	R0	Stage IB	M0	N0	T2a	Yes	Alive	MALE	Not Evaluated	60	1106
TCGA-77-A5FZ-01A-31R-A27Q-07	Tumor	LUSC	R0	Stage IIIB	M0	N0	T4	Yes	Dead	MALE	Not Evaluated	64	3838
TCGA-77-A5G1-01A-11R-A27Q-07	Tumor	LUSC	R0	Stage IIIA	M0	N1	T3	Yes	Alive	MALE	Not Evaluated	75	4026
TCGA-77-A5G3-01A-31R-A27Q-07	Tumor	LUSC	R0	Stage IIB	M0	N1	T2	Yes	Alive	MALE	Not Evaluated	63	4570
TCGA-77-A5G6-01A-11R-A27Q-07	Tumor	LUSC	R0	Stage IIIA	M0	N2	T2	Yes	Dead	MALE	Not Evaluated	66	678

TCGA-77-A5G7-01B-11R-A27Q-07	Tumor	LUSC	R0	Stage IA	M0	N0	T1	Yes	Dead	MALE	Not Evaluated	63	180
TCGA-77-A5G8-01B-11R-A27Q-07	Tumor	LUSC	R0	Stage IIB	M0	N0	T3	Yes	Alive	MALE	Not Evaluated	70	1884
TCGA-77-A5GA-01A-11R-A27Q-07	Tumor	LUSC	R0	Stage IB	M0	N0	T2a	Yes	Alive	MALE	Not Evaluated	76	1280
TCGA-77-A5GB-01B-11R-A27Q-07	Tumor	LUSC	R0	Stage IB	M0	NX	T2a	Yes	Dead	MALE	Not Evaluated	90	937
TCGA-77-A5GF-01A-21R-A27Q-07	Tumor	LUSC	R0	Stage IIA	M0	N1	T2a	Yes	Alive	MALE	Not Evaluated	70	840
TCGA-77-A5GH-01A-11R-A27Q-07	Tumor	LUSC	R0	Stage IB	M0	N0	T2a	Yes	Alive	MALE	Not Evaluated	81	1182
TCGA-79-5596-01A-31R-1949-07	Tumor	LUSC	R0	Stage IIIA	M0	N1	T3	Yes	Alive	MALE	Not Available	Not Available	
TCGA-85-6175-01A-11R-1820-07	Tumor	LUSC	R0	Stage IIB	M0	N0	T3	No	Alive	FEMALE	WHITE	63	294

TCGA-85-6560-01A-11R-1820-07	Tumor	LUSC	R0	Stage IIA	M0	N1	T1b	Yes	Alive	MALE	WHITE	59	1259
TCGA-85-6561-01A-11R-1820-07	Tumor	LUSC	R0	Stage IB	M0	NX	T2a	Yes	Alive	MALE	WHITE	66	1224
TCGA-85-6798-01A-11R-1949-07	Tumor	LUSC	R0	Stage IIIA	M0	N1	T3	Yes	Alive	MALE	WHITE	57	195
TCGA-85-7696-01A-11R-2125-07	Tumor	LUSC	R0	Stage IA	M0	N0	T1	Yes	Alive	MALE	WHITE	64	1111
TCGA-85-7697-01A-11R-2125-07	Tumor	LUSC	R0	Stage IIB	M0	N0	T3	Yes	Alive	MALE	WHITE	49	1063
TCGA-85-7698-01A-11R-2125-07	Tumor	LUSC	R0	Stage IA	M0	N0	T1b	Yes	Alive	MALE	WHITE	48	952
TCGA-85-7699-01A-11R-2125-07	Tumor	LUSC	R0	Stage IIIA	M0	N0	T4	Yes	Alive	MALE	WHITE	73	1001
TCGA-85-7710-01A-11R-2125-07	Tumor	LUSC	Not Available	Stage IA	M0	N0	T1b	Yes	Alive	FEMALE	WHITE	59	42

TCGA-85-7710-11A-01R-2125-07	Normal	LUSC	Not Available	Stage IA	M0	N0	T1b	Yes	Alive	FEMALE	WHITE	59	42
TCGA-85-7843-01A-11R-2125-07	Tumor	LUSC	R0	Stage IIA	M0	N1	T2a	Yes	Alive	MALE	WHITE	50	35
TCGA-85-7844-01A-11R-2125-07	Tumor	LUSC	R0	Stage IB	M0	N0	T2a	Yes	Alive	MALE	WHITE	71	911
TCGA-85-7950-01A-11R-2187-07	Tumor	LUSC	RX	Stage IB	M0	N0	T2a	Yes	Alive	MALE	WHITE	46	576
TCGA-85-8048-01A-11R-2247-07	Tumor	LUSC	R0	Stage IA	M0	N0	T1	Yes	Alive	MALE	WHITE	62	765
TCGA-85-8049-01A-11R-2247-07	Tumor	LUSC	R0	Stage IB	M0	N0	T2a	No	Alive	MALE	WHITE	57	579
TCGA-85-8052-01A-11R-2247-07	Tumor	LUSC	R0	Stage IIB	M0	N0	T3	Yes	Alive	MALE	WHITE	53	734
TCGA-85-8070-01A-11R-2247-07	Tumor	LUSC	R0	Stage IB	M0	N0	T2	Yes	Alive	MALE	WHITE	71	960

TCGA-85-8071-01A-11R-2247-07	Tumor	LUSC	R0	Stage IIA	M0	N1	T1a	Yes	Alive	MALE	WHITE	52	815
TCGA-85-8072-01A-31R-2247-07	Tumor	LUSC	R0	Stage IA	M0	N0	T1a	Yes	Alive	MALE	WHITE	60	932
TCGA-85-8276-01A-11R-2296-07	Tumor	LUSC	R0	Stage IIA	M0	N1	T1b	Yes	Alive	MALE	WHITE	62	1050
TCGA-85-8277-01A-11R-2296-07	Tumor	LUSC	R0	Stage IIIA	M0	N1	T3	No	Alive	MALE	WHITE	70	307
TCGA-85-8287-01A-11R-2296-07	Tumor	LUSC	R0	Stage IA	M0	N0	T1b	Yes	Alive	MALE	WHITE	72	23
TCGA-85-8288-01A-11R-2296-07	Tumor	LUSC	R0	Stage IIA	M0	N1	T1b	Yes	Alive	MALE	WHITE	70	402
TCGA-85-8350-01A-11R-2296-07	Tumor	LUSC	R0	Stage IA	M0	N0	T1b	Yes	Alive	MALE	WHITE	61	683
TCGA-85-8351-01A-11R-2296-07	Tumor	LUSC	R0	Stage IIA	M0	N1	T2a	Yes	Alive	MALE	WHITE	72	510

TCGA-85-8352-01A-31R-2326-07	Tumor	LUSC	R0	Stage IIIA	M0	N1	T3	Yes	Alive	MALE	WHITE	67	161
TCGA-85-8353-01A-21R-2296-07	Tumor	LUSC	R0	Stage IIIA	M0	N1	T3	Yes	Alive	MALE	WHITE	72	94
TCGA-85-8354-01A-31R-2326-07	Tumor	LUSC	R0	Stage IB	M0	N0	T2a	Yes	Alive	MALE	WHITE	53	995
TCGA-85-8355-01A-11R-2296-07	Tumor	LUSC	R0	Stage IA	M0	N0	T1a	No	Alive	MALE	WHITE	63	61
TCGA-85-8479-01A-11R-2326-07	Tumor	LUSC	R0	Stage IA	M0	N0	T1a	Yes	Alive	MALE	WHITE	66	468
TCGA-85-8481-01A-11R-2326-07	Tumor	LUSC	R0	Stage IIB	M0	N0	T3	Yes	Alive	MALE	WHITE	70	236
TCGA-85-8580-01A-31R-2403-07	Tumor	LUSC	R0	Stage IB	M0	N0	T2a	Yes	Alive	FEMALE	WHITE	52	1113
TCGA-85-8582-01A-21R-2403-07	Tumor	LUSC	R0	Stage IA	M0	N0	T1a	Yes	Alive	MALE	WHITE	49	1160

TCGA-85-8584-01A-11R-2403-07	Tumor	LUSC	R0	Stage IIA	M0	N1	T2a	Yes	Alive	MALE	WHITE	71	383
TCGA-85-8664-01A-11R-2403-07	Tumor	LUSC	R0	Stage IIB	M0	N1	T2b	Yes	Alive	MALE	WHITE	73	550
TCGA-85-8666-01A-11R-2403-07	Tumor	LUSC	R0	Stage IB	M0	N0	T2a	Yes	Alive	MALE	WHITE	65	687
TCGA-85-A4CL-01A-41R-A26W-07	Tumor	LUSC	R0	Stage IA	M0	N0	T1b	Yes	Alive	MALE	WHITE	65	921
TCGA-85-A4CN-01A-11R-A24H-07	Tumor	LUSC	R0	Stage IIB	M0	N1	T2b	Yes	Alive	FEMALE	WHITE	56	1038
TCGA-85-A4JB-01A-51R- A262-07	Tumor	LUSC	R0	Stage IIB	M0	N0	T3	Yes	Alive	MALE	WHITE	74	942
TCGA-85-A4JC-01A-11R- A24Z-07	Tumor	LUSC	R0	Stage IIA	M0	N0	T2b	Yes	Alive	MALE	WHITE	84	604
TCGA-85-A4PA-01A-11R- A24Z-07	Tumor	LUSC	R0	Stage IB	M0	N0	T2a	Yes	Alive	MALE	WHITE	61	741

TCGA-85-A4QQ-01A-41R-A262-07	Tumor	LUSC	R0	Stage IB	M0	N0	T2a	Yes	Alive	MALE	WHITE	68	927
TCGA-85-A4QR-01A-11R-A24Z-07	Tumor	LUSC	R0	Stage IB	M0	N0	T2a	Yes	Alive	MALE	WHITE	67	600
TCGA-85-A50M-01A-21R-A262-07	Tumor	LUSC	R0	Stage IIA	M0	N0	T2b	Yes	Alive	MALE	ASIAN	47	826
TCGA-85-A50Z-01A-21R- A262-07	Tumor	LUSC	R0	Stage IIA	M0	N0	T2b	Yes	Alive	MALE	WHITE	57	493
TCGA-85-A510-01A-11R- A26W-07	Tumor	LUSC	R0	Stage IIB	M0	N1	T2b	Yes	Alive	FEMALE	WHITE	74	482
TCGA-85-A511-01A-21R- A262-07	Tumor	LUSC	R0	Stage IIB	M0	N1	T2b	Yes	Alive	MALE	WHITE	62	455
TCGA-85-A512-01A-11R- A26W-07	Tumor	LUSC	R0	Stage IIA	M0	N1	T1b	Yes	Alive	MALE	WHITE	46	465
TCGA-85-A513-01A-12R- A26W-07	Tumor	LUSC	R0	Stage IA	M0	NX	T1a	No	Alive	FEMALE	ASIAN	60	910

TCGA-85-A53L-01A-21R- A26W-07	Tumor	LUSC	R0	Stage IIA	M0	N0	T2b	Yes	Alive	MALE	ASIAN	63	377
TCGA-85-A5B5-01A-21R-A26W-07	Tumor	LUSC	R0	Stage IA	M0	N0	T1b	Yes	Alive	MALE	WHITE	58	111
TCGA-90-6837-01A-11R-1949-07	Tumor	LUSC	R0	Stage IIB	MX	N0	T3	Yes	Alive	MALE	WHITE	64	758
TCGA-90-6837-11A-01R-1949-07	Normal	LUSC	R0	Stage IIB	MX	N0	T3	Yes	Alive	MALE	WHITE	64	758
TCGA-90-7766-01A-21R-2125-07	Tumor	LUSC	R0	Stage IA	MX	N0	T1b	Yes	Alive	FEMALE	WHITE	66	289
TCGA-90-7767-01A-11R-2125-07	Tumor	LUSC	R0	Stage IIB	MX	N1	T2b	Yes	Alive	MALE	WHITE	56	89
TCGA-90-7767-11A-01R-2125-07	Normal	LUSC	R0	Stage IIB	MX	N1	T2b	Yes	Alive	MALE	WHITE	56	89
TCGA-90-7769-01A-11R-2125-07	Tumor	LUSC	R0	Stage IIB	MX	N1	T2b	Yes	Alive	MALE	WHITE	55	358

TCGA-90-7964-01A-21R-2187-07	Tumor	LUSC	R0	Stage IB	MX	N0	T2a	Yes	Alive	MALE	WHITE	70	428
TCGA-90-A4ED-01A-31R-A24Z-07	Tumor	LUSC	Not Evaluated	Stage IB	MX	N0	T2a	Yes	Alive	MALE	WHITE	69	615
TCGA-90-A4EE-01A-11R-A24Z-07	Tumor	LUSC	Not Evaluated	Stage IIA	MX	N1	T2a	Yes	Alive	MALE	WHITE	53	688
TCGA-90-A59Q-01A-11R-A26W-07	Tumor	LUSC	R0	Stage IIA	MX	N1	T2a	Yes	Alive	FEMALE	WHITE	61	322
TCGA-92-7340-01A-21R-2045-07	Tumor	LUSC	R1	Stage IIA	MX	N1	T2a	Yes	Alive	FEMALE	WHITE	45	82
TCGA-92-7340-11A-01R-2045-07	Normal	LUSC	R1	Stage IIA	MX	N1	T2a	Yes	Alive	FEMALE	WHITE	45	82
TCGA-92-7341-01A-31R-2045-07	Tumor	LUSC	R0	Stage IB	MX	N0	T2a	Yes	Alive	MALE	WHITE	71	106
TCGA-92-8063-01A-11R-2247-07	Tumor	LUSC	RX	Stage IIIA	MX	N2	T2b	Yes	Alive	MALE	WHITE	52	122

TCGA-92-8064-01A-11R-2247-07	Tumor	LUSC	RX	Discrepancy	MX	N0	T2b	Yes	Alive	MALE	WHITE	58	160
TCGA-92-8065-01A-11R-2247-07	Tumor	LUSC	RX	Stage IIB	MX	N0	T3	Yes	Alive	FEMALE	WHITE	74	70
TCGA-94-7033-01A-11R-1949-07	Tumor	LUSC	R0	Stage IB	MX	N0	T2	Yes	Alive	MALE	WHITE	73	640
TCGA-94-7557-01A-11R-2125-07	Tumor	LUSC	Not Available	Stage IB	M0	N0	T2	Yes	Dead	MALE	BLACK OR AFRICAN AMERICAN	73	5
TCGA-94-7943-01A-11R-2187-07	Tumor	LUSC	R0	Stage IA	MX	NX	T1b	Yes	Alive	MALE	WHITE	80	559
TCGA-94-8035-01A-11R-2247-07	Tumor	LUSC	R0	Stage IIB	MX	N0	T3	Yes	Alive	MALE	WHITE	64	122
TCGA-94-8490-01A-11R-2326-07	Tumor	LUSC	R0	Stage IIB	M0	N0	T3	Yes	Alive	MALE	WHITE	70	153
TCGA-94-8491-01A-11R-2326-07	Tumor	LUSC	Not Available	Stage IIA	M0	N1	T2a	Yes	Alive	MALE	WHITE	73	810

TCGA-94-A4VJ-01A-11R- A24Z-07	Tumor	LUSC	Not Available	Stage IA	M0	N0	T1b	Yes	Alive	FEMALE	WHITE	71	430
TCGA-94-A5I4- 01A-11R- A26W-07	Tumor	LUSC	RX	Stage IIA	MX	N1	T2a	Yes	Alive	MALE	WHITE	61	491
TCGA-94-A5I6-01A-21R-A27Q-07	Tumor	LUSC	RX	Stage IIB	M0	N0	T3	Yes	Alive	MALE	WHITE	62	538
TCGA-96-7544-01A-11R-2045-07	Tumor	LUSC	Not Available	Stage IIB	MX	N1	T2	Yes	Alive	MALE	WHITE	83	2160
TCGA-96-7545-01A-21R-2045-07	Tumor	LUSC	Not Available	Stage IA	MX	N0	T1	Yes	Alive	MALE	WHITE	73	1736
TCGA-96-8169-01A-11R-2296-07	Tumor	LUSC	Not Available	Stage IA	M0	N0	T1a	Yes	Alive	FEMALE	WHITE	67	557
TCGA-96-8170-01A-11R-2296-07	Tumor	LUSC	Not Available	Stage IIA	M0	N1	T1a	Yes	Alive	FEMALE	WHITE	75	531
TCGA-96-A4JK-01A-11R- A24Z-07	Tumor	LUSC	R0	Stage IIA	M0	N1	T2a	Yes	Alive	MALE	WHITE	65	589

TCGA-96-A4JL-01A-11R- A24Z-07	Tumor	LUSC	R0	Stage IIA	M0	N1	T2a	No	Alive	FEMALE	ASIAN	78	842
TCGA-98-7454-01A-11R-2045-07	Tumor	LUSC	R0	Stage IB	M0	N0	T2a	Yes	Alive	MALE	WHITE	73	652
TCGA-98-8020-01A-11R-2247-07	Tumor	LUSC	R0	Stage IIIA	M0	N2	T2	No	Dead	FEMALE	BLACK OR AFRICAN AMERICAN	56	84
TCGA-98-8021-01A-11R-2247-07	Tumor	LUSC	R0	Stage IA	M0	N0	T1a	Yes	Alive	FEMALE	WHITE	75	937
TCGA-98-8022-01A-11R-2247-07	Tumor	LUSC	R0	Stage IA	M0	N0	T1a	Yes	Alive	MALE	WHITE	61	933
TCGA-98-8023-01A-11R-2247-07	Tumor	LUSC	R0	Stage IIIA	M0	N1	T3	Yes	Alive	MALE	WHITE	70	649
TCGA-98-A538-01A-11R- A262-07	Tumor	LUSC	R0	Stage IIB	M0	N0	T3	Yes	Alive	MALE	WHITE	67	826

TCGA-98-A539-01A-31R- A262-07	Tumor	LUSC	R0	Stage IIB	M0	N0	T3	Yes	Alive	MALE	WHITE	63	173
TCGA-98-A53A-01A-11R- A262-07	Tumor	LUSC	R0	Stage IB	M0	N0	T2a	Yes	Alive	MALE	BLACK OR AFRICAN AMERICAN	70	552
TCGA-98-A53B-01A-11R- A262-07	Tumor	LUSC	R0	Stage IB	M0	N0	T2a	Yes	Dead	MALE	WHITE	69	61
TCGA-98-A53C-01A-11R-A262-07	Tumor	LUSC	R0	Stage IA	M0	N0	T1a	Yes	Alive	FEMALE	WHITE	77	822
TCGA-98-A53D-01A-32R-A262-07	Tumor	LUSC	R0	Stage IIB	M0	N0	T3	Yes	Alive	MALE	WHITE	68	645
TCGA-98-A53H-01A-12R-A262-07	Tumor	LUSC	R0	Stage IA	M0	N0	T1a	Yes	Alive	FEMALE	WHITE	76	618
TCGA-98-A53I- 01A-31R-A262-07	Tumor	LUSC	R0	Stage IIA	M0	N1	T2a	Yes	Alive	MALE	WHITE	64	565

TCGA-98-A53J-01A-11R- A26W-07	Tumor	LUSC	R0	Stage IB	M0	N0	T2a	Yes	Alive	MALE	WHITE	77	630
TCGA-J1-A4AH-01A-31R-A24H-07	Tumor	LUSC	R0	Stage IIA	MX	N0	T2b	Yes	Alive	MALE	WHITE	70	581
TCGA-L3-A4E7-01A-11R- A24Z-07	Tumor	LUSC	R0	Stage IB	M0	N0	T2a	Yes	Alive	MALE	WHITE	71	392
TCGA-L3-A524-01A-11R- A262-07	Tumor	LUSC	R0	Stage IIB	M0	N0	T3	Yes	Alive	FEMALE	WHITE	45	490
TCGA-LA-A446-01A-21R- A24Z-07	Tumor	LUSC	R0	Stage IA	MX	N0	T1b	Yes	Alive	MALE	WHITE	68	401
TCGA-LA-A7SW-01A-11R-A405-07	Tumor	LUSC	R0	Stage IIIA	MX	N1	T3	Yes	Alive	MALE	BLACK OR AFRICAN AMERICAN	71	408
TCGA-MF-A522-01A-11R- A262-07	Tumor	LUSC	Not Available	Stage IB	MX	N0	T2a	Yes	Alive	MALE	WHITE	54	360
TCGA-NC-A5HD-01A-11R-A26W-07	Tumor	LUSC	R0	Stage IIB	M0	N0	T3	Yes	Dead	MALE	WHITE	79	2

TCGA-NC-A5HE-01A-11R-A26W-07	Tumor	LUSC	R0	Stage IIB	M0	N1	T2	Yes	Alive	MALE	WHITE	60	2336
TCGA-NC-A5HF-01A-11R-A26W-07	Tumor	LUSC	R0	Stage IIIB	MX	N0	T4	Yes	Dead	MALE	WHITE	74	138
TCGA-NC-A5HG-01A-11R-A26W-07	Tumor	LUSC	R0	Stage IIIA	M0	N2	T2	Yes	Alive	MALE	WHITE	59	1963
TCGA-NC-A5HH-01A-11R-A26W-07	Tumor	LUSC	R0	Stage IA	M0	N0	T1	Yes	Alive	MALE	WHITE	53	37
TCGA-NC-A5HI-01A-11R- A26W-07	Tumor	LUSC	R0	Stage IB	M0	N0	T2	Yes	Alive	FEMALE	WHITE	68	1743
TCGA-NC-A5HJ-01A-11R- A26W-07	Tumor	LUSC	R0	Stage IIB	M0	N0	T3	Yes	Dead	MALE	WHITE	59	418
TCGA-NC-A5HK-01A-11R-A26W-07	Tumor	LUSC	R0	Stage IIB	M0	N0	T3	Yes	Alive	FEMALE	WHITE	58	128
TCGA-NC-A5HL-01A-11R-A26W-07	Tumor	LUSC	R0	Stage IIA	M0	N0	T2b	Yes	Dead	MALE	WHITE	73	88

TCGA-NC-A5HM-01A-12R-A26W-07	Tumor	LUSC	R0	Stage IB	M0	N0	T2a	Yes	Alive	MALE	WHITE	76	1212
TCGA-NC-A5HN-01A-11R-A26W-07	Tumor	LUSC	R0	Stage IIA	M0	N1	T2a	Yes	Alive	MALE	WHITE	77	1499
TCGA-NC-A5HO-01A-11R-A26W-07	Tumor	LUSC	Not Evaluated	Stage IIIA	M0	N1	T3	Yes	Alive	FEMALE	WHITE	70	1336
TCGA-NC-A5HP-01A-11R-A26W-07	Tumor	LUSC	R0	Stage IV	M1b	N0	T2a	Yes	Alive	MALE	WHITE	69	770
TCGA-NC-A5HQ-01A-11R-A26W-07	Tumor	LUSC	R0	Stage IIIA	M0	N2	T3	Yes	Dead	MALE	WHITE	70	448
TCGA-NC-A5HR-01A-21R-A26W-07	Tumor	LUSC	R0	Stage IIA	M0	N1	T2a	Yes	Alive	FEMALE	WHITE	75	1244
TCGA-NC-A5HT-01A-11R-A26W-07	Tumor	LUSC	R0	Stage IIIA	M0	N1	T3	Yes	Alive	MALE	WHITE	69	804

TCGA-NK-A5CR-01A-11R-A26W-07	Tumor	LUSC	Not Evaluated	Stage IB	MX	N0	T2	Yes	Alive	MALE	Unknown	77	2542
TCGA-NK-A5CT-01A-31R-A26W-07	Tumor	LUSC	Not Evaluated	Stage IA	M0	N0	T1	Discrepancy	Alive	MALE	WHITE	70	1997
TCGA-NK-A5CX-01A-11R-A26W-07	Tumor	LUSC	Not Evaluated	Stage IIA	MX	N0	T2b	Yes	Alive	MALE	WHITE	73	111
TCGA-NK-A5D1-01A-11R-A26W-07	Tumor	LUSC	Not Evaluated	Stage IIA	M0	N1	T2a	Discrepancy	Alive	MALE	WHITE	57	511
TCGA-NK-A7XE-01A-12R-A405-07	Tumor	LUSC	Not Available	Stage IIIB	M0	N2	T4	Yes	Alive	MALE	BLACK OR AFRICAN AMERICAN	66	13
TCGA-O2-A52N-01A-11R-A26W-07	Tumor	LUSC	Not Available	Stage I	MX	N0	T2	Yes	Dead	MALE	WHITE	78	1006
TCGA-O2-A52Q-01A-11R-A26W-07	Tumor	LUSC	Not Evaluated	Stage III	MX	N1	T3	Yes	Dead	FEMALE	WHITE	44	113
TCGA-O2-A52S-01A-11R- A262-07	Tumor	LUSC	Not Evaluated	Stage III	MX	N2	T4	Yes	Dead	FEMALE	WHITE	57	387

TCGA-O2-A52V-01A-31R- A262-07	Tumor	LUSC	Not Evaluated	Stage II	MX	N0	T3	Yes	Dead	FEMALE	BLACK OR AFRICAN AMERICAN	75	1335
TCGA-O2-A52W-01A-11R-A26W-07	Tumor	LUSC	Not Evaluated	Stage I	MX	N0	T2	Yes	Dead	MALE	BLACK OR AFRICAN AMERICAN	63	261
TCGA-O2-A5IB-01A-11R- A27Q-07	Tumor	LUSC	Not Evaluated	Stage III	MX	N1	T3	Yes	Dead	FEMALE	WHITE	71	340
TCGA-XC-AA0X-01A-32R-A405-07	Tumor	LUSC	R0	Stage IA	M0	N0	T1a	Yes	Dead	FEMALE	BLACK OR AFRICAN AMERICAN	77	6

Table S2. Clinicopathological Characteristics in 90 Patients with LUAD and 60 Patients with LUSC.

Characteristics		LUAD Patients (n=90)	LUSC Patients (n=60)
Age (years)	<=65	49	22
	>65	41	38
Gender	Male	50	45
	Female	40	15
Smoker	Yes	52	45
	No	38	15
T stage	T1	31	8
	T2	47	40
	T3	9	10
	T4	3	2
N stage	N0	54	39
	N1	25	18
	N2	11	3
	N3	0	0
M stage	M0	90	57
	M1	0	3
Pathologic stage	I	44	31
	II	28	18
	III	15	8
	IV	3	3
Differentiation	Well	25	24
	Moderate	42	32
	Poor	33	4
PSRC1 Expression			
tumor tissue	No or low expression	54	39
	high expression	36	21

PSRC1, Proline and serine rich coiled-coil 1; LUAD, lung adenocarcinoma. LUSC, lung squamous cell carcinoma
